# Glycosidase-activated prodrugs of a cytotoxic iron chelator for targeted cancer therapy†

**DOI:** 10.1039/d5md00232j

**Published:** 2025-06-19

**Authors:** Debashish Tomar, Axel Steinbrueck, Adam C. Sedgwick, Matthew S. Levine, Jonathan L. Sessler, Nils Metzler-Nolte

**Affiliations:** a Faculty of Chemistry and Biochemistry, Inorganic Chemistry I – Bioinorganic chemistry, Ruhr-University Bochum Universitaetsstrasse 150 44801 Bochum Germany nils.metzler-nolte@ruhr-uni-bochum.de; b Department of Chemistry, King's College London 7 Trinity Street London SE1 1DB UK; c Department of Chemistry, University of Texas at Austin 105 E 24th street A5300 Austin TX 78712-1224 USA

## Abstract

New glycoside-prodrugs based on the iron chelator deferasirox were designed. Selective enzymatic activation by glycosidases was observed within 24 hours, accompanied by cancer cell-selective cytotoxicity. Notably, derivative 3a, bearing a β-d-galactose moiety, showed promising selective activity against galactosidase overexpressing OvCar-3 cells (IC_50_ 9.1 ± 1.6 μM) while maintaining low activity against fibroblast control GM5756 cells (IC_50_ > 100 μM).

## Abstract 2

New glycoside-conjugated prodrugs based on the iron chelator deferasirox were designed and their selective cleavage by glycosidases as well as cancer cell line-specific toxicity was demonstrated.

## Graphical abstract

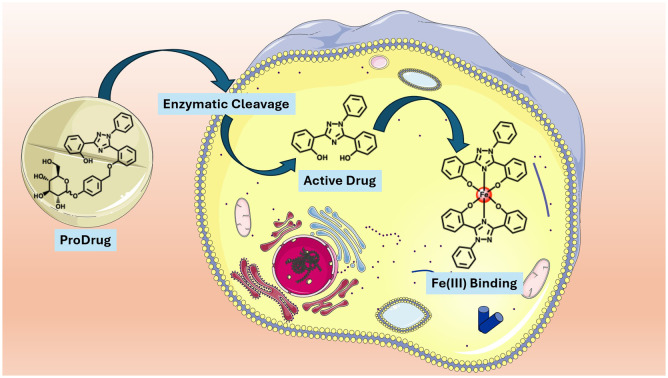

Cancer continues to pose a significant global health challenge, with 19.3 million new cases and 9.6 million deaths reported in 2020, according to the World Health Organization (WHO).^1^ Current treatment modalities, such as chemotherapy and radiation therapy, are often limited by severe off-target effects as well as the emergence of resistance mechanisms in cancer cells.^2–4^ These challenges necessitate the development of new therapies that selectively target malignant cells while preserving healthy tissue.

A promising new treatment approach is the intentional complexation of bio-relevant transition metals by selective chelators.^5–8^ Within this arena, specifically the Fe(iii)-chelator deferasirox has shown promising results by suppressing the growth of cancer cells *in vitro*, *in vivo*, and in individual clinical cases.^9–12^ In our previous work we were able to show that strategic derivatization of deferasirox enhances the cytotoxic profile of this chelator.^13,14^ However, the selectivity of deferasirox derivatives for cancer cells remains limited and improved targeting strategies are urgently required.

Prodrug therapy has emerged as a promising strategy to enhance the specificity and safety of cancer treatments.^15,16^ For instance, cancer cells exhibit increased expression of specific glucose transporters—a hallmark of the “Warburg effect”—and overexpression of specific hydrolase enzymes, which degrade carbohydrates.^17^ These characteristics provide an opportunity to design glycosidase-activated prodrugs that selectively target cancer cells while sparing healthy tissues.^18^

Carbohydrate-based prodrugs have been extensively studied for their ability to enhance pharmacokinetic properties, including increased water solubility, reduced toxicity, and improved biocompatibility.^19–21^ Glycosylation of cytotoxic agents such as glufosfamide, chlorambucil, docetaxel, and paclitaxel has provided agents with reduced toxicity toward noncancerous cells that retain therapeutic efficacy.^22^ Building on this approach, novel glucose-conjugates have been developed to selectively target cancer cells by exploiting the overexpression of glucose transporters—a hallmark of many tumours.^23^ For instance, reported platinum-glucose conjugates have been shown to offer dual benefits: preferential accumulation in cancer cells and significantly reduced toxicity in healthy cells.^24^

In this study, we developed two variants of glycoside-prodrugs targeting the enzymatic expression of β-glucosidase and β-galactosidase, respectively, that utilize scaffold 1, a derivative of the clinical Fe(iii) chelator deferasirox,^25,26^ as the cytotoxic moiety (Fig. 1).^11,27^ Chelator 1 is particularly well-suited for this application due to its favourable anti-proliferative activity profile combined with its intrinsic fluorescence as an aggregation-induced emission (AIE) fluorophore.^10,28–31^

**Fig. 1:**
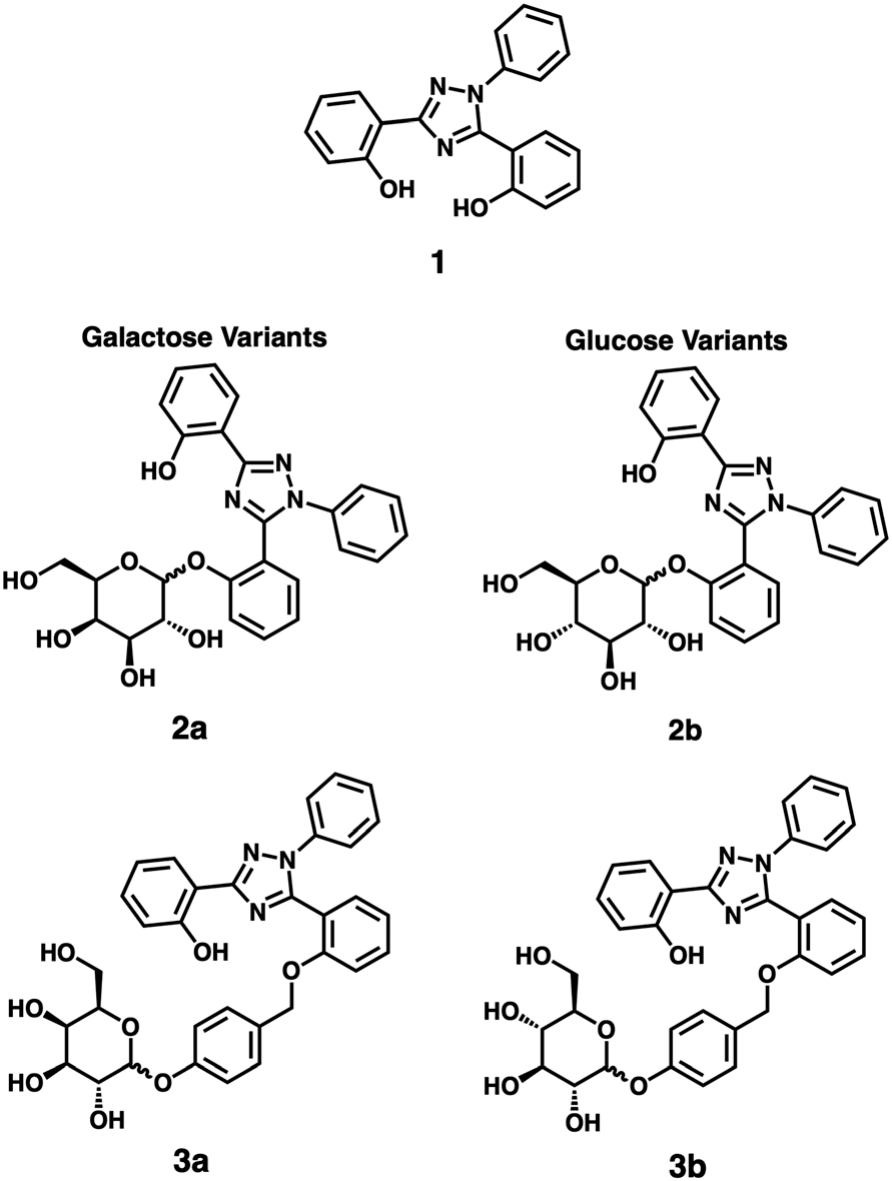
Structures of 1, 2a, 2b, 3a, and 3b.

Our first prodrug designs featured a direct glycosidic bond between the phenolic binding site of the iron chelator and the respective sugar moiety (compounds 2a/b in Fig. 1). These prodrugs showed good aqueous solubility and effective suppression of chelator-based cytotoxicity until cleavage of the sugar moiety (*vide infra*). However, the slow enzymatic cleavage and resulting poor *in vitro* activation of 2a/b prompted us to create a second generation of prodrugs, which employed a well-established self-immolating phenolic linker motif to reduce steric hindrance around the anomeric carbon and thereby enhance enzymatic cleavage rates (compounds 3a/b in Fig. 1, see also Scheme S2 in the ESI† for the reported putative mechanism of the self-immolative linker motif).^32–35^ All prodrugs were evaluated for their stability, enzyme selectivity, and specific cytotoxicity in two selected cancer cell lines as discussed below.

The complete synthesis and characterization of all prodrugs is detailed in the ESI† (Scheme S1; see Scheme 1 for an overview of the syntheses; herein all galactose-bearing compounds are labelled with “a” and the glucose variants with “b”). The cytotoxic iron chelator 1 was prepared following reported literature procedures.^36^ For the synthesis of 2a and 2b, chelator 1 was conjugated to the acetyl-protected sugars using sodium hydride as a base, followed by deprotection of the acetate groups with sodium methoxide to give the free alcohols.^37^ For derivatives 3a and 3b, 4-hydroxybenzaldehyde was initially attached to the acetyl-protected sugar using silver oxide.^38^ The resulting aldehyde was subsequently reduced to the corresponding alcohol with sodium borohydride, which was then brominated with phosphorus tribromide. The sugar bearing the self-immolating linker motif was then attached using a method analogous to that used to prepare 2a and 2b.

**Scheme 1:**
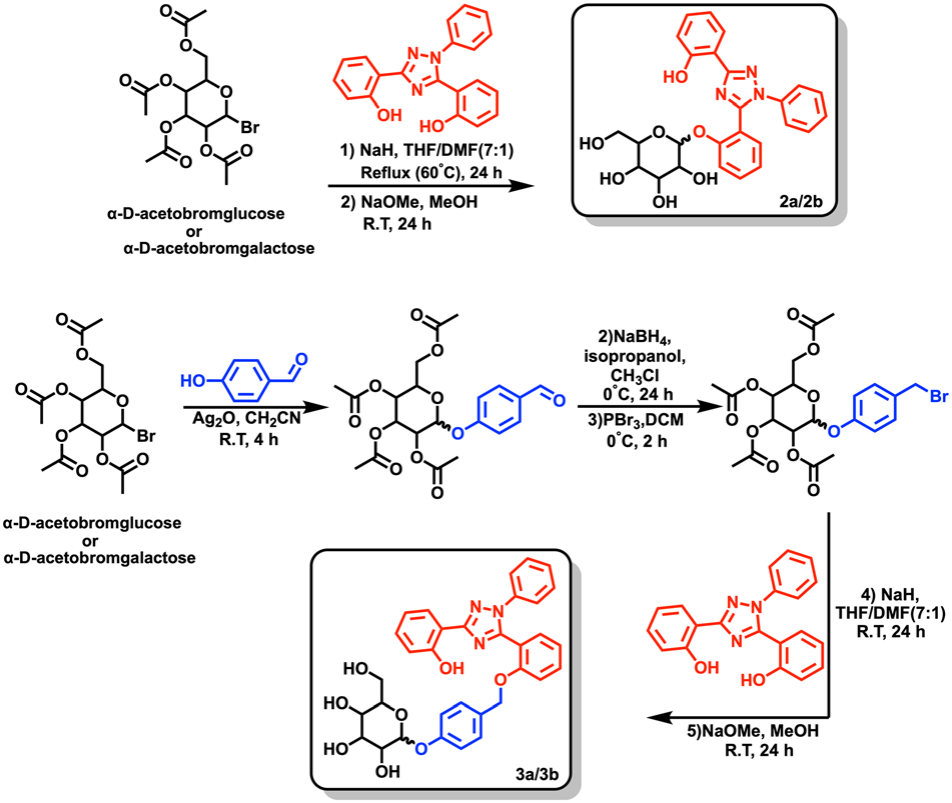
Synthetic overview of the preparation of prodrugs 2a, 2b, 3a, and 3b.

With the prodrugs in hand, binding studies were carried out using UV-vis spectroscopy; this was done to confirm the Fe(iii) binding ability of all new compounds. Initially, 1 was dissolved in the presence of excess FeCl_3_, which produced the previously reported and characteristic absorption band at 510 nm, indicating successful iron chelation by 1. To evaluate whether 2a, 2b, 3a, and 3b were chelating Fe(iii), each prodrug was similarly dissolved in a solution containing excess FeCl_3_. The results showed no relevant absorbance at 510 nm for any of the prodrugs (Fig. 2), confirming that the characteristic Fe(iii) binding *via* the O–N–O donor set of the chelator was efficiently suppressed in the prodrug form.^39^

**Fig. 2:**
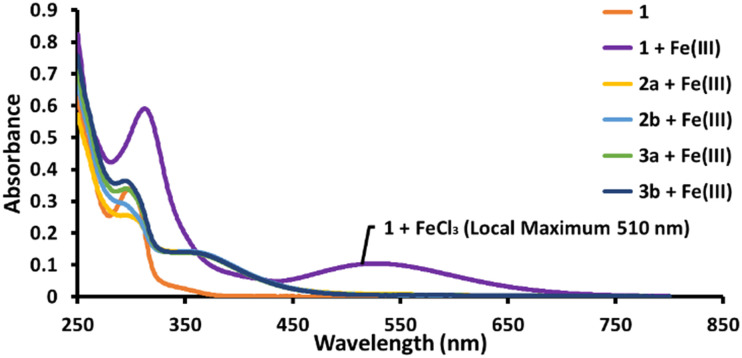
UV-vis absorption spectra for prodrugs 2a/b and 3a/b as well as 1 at a concentration of 30 μM with an excess of FeCl_3_ as Fe(iii) source (50 μM) in methanol.

To test the desired, selective enzymatic cleavage and further evaluate compound stability, solutions of each prodrug were prepared in phosphate-buffered saline (PBS), and either β-galactosidase or β-glucosidase were added. After incubation times of 24 h and 48 h, samples from each mixture were analyzed by HPLC to gain insight into the relative amounts of both the remaining prodrug and the liberated 1. Results are shown in Fig. 3.

**Fig. 3:**
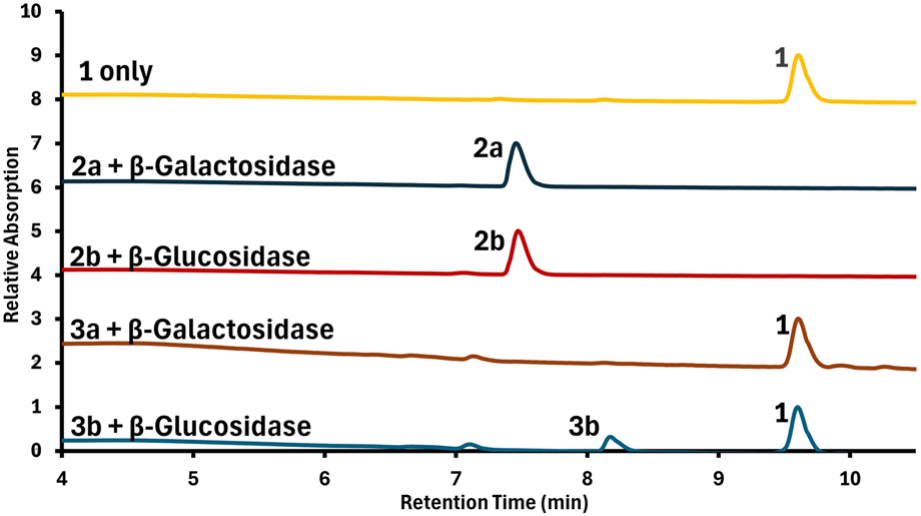
HPLC traces after 48 h of enzyme incubation for the prodrugs of this study as indicated at their respective peaks. The identity of the employed enzyme is indicated on the left of the chromatogram and the identity of each observed compound is shown above the respective peak. Chromatograms are shown with an offset of 2 along the ordinate to facilitate comparison.

For 2a and 2b, no release of 1 was observed at either 24 or 48 hours, indicating a lack of enzymatic activation. By contrast, 3a and 3b showed significant release of 1 at 24 hours, which further increased at 48 hours. Additional selectivity control experiments were conducted, in which either no enzyme or an “incorrect” enzyme such as an esterase was used (see Fig. S13–S24 in the ESI†). These results further confirm that the prodrugs are stable in PBS for up to 48 hours and only the presence of the matching glycosidase enzyme leads to effective release of the cytotoxic chelator.

The AIE fluorescence properties of 1 were used as a means of supporting the above conclusion. The emission of all glycosylated prodrugs is suppressed. Thus, solutions of each prodrug were prepared in water, and three enzyme units of β-galactosidase or β-glucosidase, as appropriate, were added to the prodrugs. Each mixture was then analysed by fluorescence spectroscopy after 24 h of incubation. The results are shown in Fig. 4. For 2a and 2b, no fluorescence emission was observed either at 0 h or at 24 h. This finding is consistent with a lack of enzymatic activation. In contrast, 3a showed a readily discernible release of 1 after 24 h, which was evidenced by the emergence of the emission band at 480 nm characteristic of 1. For 3b, the emission spectrum after 24 h showed a less pronounced emission that was blue-shifted with a maximum at 420 nm (see Fig. S29 in the ESI†). Nonetheless, HPLC analysis revealed the successful cleavage of 1 from 3b in this sample (see Fig. S30 in the ESI†). We thus suggest that the presence of the β-glucosidase enzyme could suppress the emissive properties of 1.

**Fig. 4:**
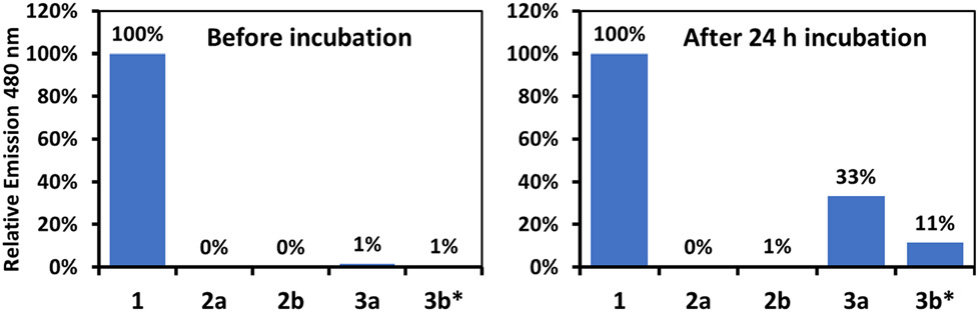
Relative emission intensity at 480 nm (*for 3b at 420 nm) of evaluated compounds at 20 μM before and after incubation with 3 U of the appropriate enzyme.

Next, the cytotoxicity of the prodrugs was assessed in the human lung cancer (A549) and human ovarian cancer (OvCar-3) cell lines: overexpressing β-glucosidase, and, overexpressing both β-glucosidase and β-galactosidase, respectively.^40,41^ For this, a standard MTT assay was employed to generate the proliferation profiles (*cf.* ESI† Fig. S1–S5) and allowed us to calculate the IC_50_ values for each compound. GM5756 fibroblast cells were included as non-cancerous control. The results are summarized in Table 1.

**Table 1:** IC_50_ for 1, 2a, 2b, 3a, and 3b against cancerous A549 and OvCar-3 cells as well as GM 5756 fibroblast cells after 72 h of incubation determined by MTT assay. Results are shown as the mean value and standard deviation of three independent experiments. In each cell line, a paired difference test (“*t*-test”) was performed

Comp.	A549	OvCar-3	GM5756
1	3.1 ± 0.4 μM	3.9 ± 1.6 μM	2.7 ± 0.1 μM
2a	74.4 ± 14.1 μM^a^	67.6 ± 13.0 μM^a^	>100 μM^a^
2b	>100 μM^a^	76.5 ± 0.8 μM^a^	>100 μM^a^
3a	44.1 ± 2.4 μM^a^	9.1 ± 1.6 μM	>100 μM^a^
3b	7.4 ± 1.5 μM	3.7 ± 0.3 μM	15.3 ± 2.3 μM^a^

a Indicates significantly decrease activity of the prodrug with respect to 1 with *p* < 0.001.

The effective cytotoxicity of 1, which served as positive control for this study, was first confirmed in A549 and OvCar-3 cancer cells, yielding IC_50_ values of 3.1 ± 0.4 μM and 3.9 ± 1.6 μM, respectively. In the non-cancerous fibroblast cell line GM5756 an IC_50_ value of 2.7 ± 0.1 μM was observed, confirming the lack of cell selectivity for the free ligand 1. These values are in good agreement with literature precedent for this chelator.^13^ Subsequently, the prodrugs 2a and 2b were assessed. Both 2a and 2b exhibited limited activity against the evaluated cell lines with IC_50_ values >20 times higher than those of the free chelator 1. The second set of prodrugs, containing the additional linker (*i.e.*, 3a and 3b), showed significantly improved IC_50_ values. Specifically, the glucose-derivative 3b demonstrated good cytotoxicity against both cancer cell lines with IC_50_ values of 7.4 ± 1.5 μM in A549 cells and 3.7 ± 0.3 μM in OvCar-3 cells, while a comparatively higher IC_50_ value was observed for the non-cancerous fibroblast cells GM5756, namely 15.3 ± 2.3 μM. Notably, 3a exhibited low activity against A549 (IC_50_ = 44.1 ± 2.4 μM) and GM5756, while exerting high cytotoxicity against galactosidase overexpressing OvCar-3 cells (IC_50_ = 9.1 ± 1.6 μM). These findings provide support for the design expectation that appropriately engineered prodrugs will prove amenable to targeted enzymatic cleavage by specific types of cancer cells. Consistent with this thinking, the glucosidase-activated derivative 3b proved active against both cancer cell lines, while 3a only showed good activity against the galactosidase-overexpressing OvCar-3 cell line.

This study highlights the potential of glycol-conjugation and glycosidase-activated prodrugs in advancing targeted cancer therapies based on Fe(iii) chelators. Our first-generation prodrugs (2a and 2b) demonstrated effective suppression of the inherent cytotoxicity of 1 combined with good stability in aqueous media. However, these compounds exhibited only limited enzymatic cleavage capacity and consequently also only minimal cytotoxicity *in vitro* when compared to the free chelator 1. To overcome this limitation, a second-generation of prodrugs (3a and 3b) was designed that contained a self-immolating linker expected to enhance enzymatic cleavage efficiency. UV-vis spectroscopy confirmed that all prodrugs suppressed effective iron binding of the chelator in their prodrug form, while enzymatic cleavage studies demonstrated significant release of 1 only from the linker-based prodrugs (*i.e.*, 3a and 3b). Cytotoxicity assays revealed improved IC_50_ values for the second-generation prodrugs and more importantly confirmed selective enzymatic activation and targeted cytotoxicity. Notably, A549 cells, which do not overexpress galactosidase, were significantly less sensitive to 3a when compared to OvCar-3 cells, underscoring the desired cell specificity. Likewise, 3b showed cytotoxicity in the both cancerous cell lines (A549 and OvCar-3) comparable to the free chelator 1, while being less active against non-cancerous fibroblast cells. These findings highlight the potential of glycosidase-activated prodrugs as a promising and selective therapeutic approach that may allow the Fe(iii) chelators to be developed further as effective cancer treatments.

## Conflicts of interest

The authors declare that they have no known competing financial interests or personal relationships that could have appeared to influence the work reported in this paper.

## Supplementary Material

MD-016-D5MD00232J-s001

## Data Availability

The authors confirm that all data are available as ESI.† Furthermore, additional data and original files are available from the authors upon reasonable request.
