# Supplementary material for: Glycosidase-activated prodrugs of a cytotoxic iron chelator for targeted cancer therapy
Source: RSC Med Chem. 2025 Jun 19;16(8):3507–11. doi: 10.1039/d5md00232j (PMC12177571; doi:10.1039/d5md00232j)
Supplement: MD-016-D5MD00232J-s001 [file MD-016-D5MD00232J-s001.pdf]

Supplementary Information (SI) for RSC Medicinal Chemistry.
This journal is © The Royal Society of Chemistry 2025

Supporting Information for MD-RES-03-2025-000232

Glycosidase-Activated Prodrugs of a Cytotoxic Iron Chelator for Targeted Cancer Therapy

Debashish Tomar[a], Axel Steinbrueck[a,c], Adam C. Sedgwick[b], Matthew S. Levine[c], Jonathan L. Sessler[c], and Nils Metzler-Nolte[*,a]

[a] Faculty of Chemistry and Biochemistry, Ruhr-University Bochum, Universitätsstraße 150, 44801, Bochum, Germany.
[b] Department of Chemistry, King's College London, 7 Trinity Street, London, SE1 1DB, United Kingdom.

[c] Department of Chemistry, University of Texas at Austin, 105 E 24th street A5300, Austin, TX 78712-1224, USA.

Email corresponding author:
nils.metzler-nolte@rub.de

Table of Contents

1. *Materials, Methods, and Instruments* 4
2. *Synthesis* 6
3. *^{1}H-NMR, ^{13}C-NMR, and HRMS* 23
4. *Cell-Viability Studies* 42
5. *UV-vis Spectroscopy* 45
6. *Enzyme Study (HPLC)* 48
7. *Fluorescence Study* 55
8. *Reference*...57

Common abbreviations used:

TLC	thin-layer chromatography
NMR	nuclear magnetic resonance
ESI-MS	electrospray ionization mass spectrometry
HPLC	high pressure liquid chromatography
TFA	trifluoroacetic acid
MTT	1-ethyl-3-(3-dimethylaminopropyl)carbodiimide hydrochloride
DMSO	dimethyl sulfoxide
PBS	phosphate-buffered saline
r.t	room temperature
THF	tetrahydrofuran
DMF	N, N-dimethylformamid
EtOAc	ethyl acetate
MeOH	methanol
DCM	dichloromethane

1. Materials, Methods, and Instruments

Chemicals. All materials were obtained from reputable commercial sources at the highest purity available and used without further purification. Reactions were carried out under air or under inert conditions, as indicated in the procedures. Flash column chromatography was performed on BUCHI Pure C-815 sorbent silica gel (40-63 µm, CHROMABOND). Analytical thin layer chromatography (TLC) analyses were carried out on aluminum-backed silica gel plates (200 µm, Sorbent Technologies).

NMR. The reported ^{1}H NMR and ^{13}C NMR spectra were measured on a Bruker spectrometer at the Ruhr University Bochum with $CDCl_3$, CD_2Cl_2, DMSO-d_6, and MeOD-d_4 as the solvents. Chemical shifts are reported relative to the residual solvent proton signals. The NMR spectra were analyzed using the software *MestReNova v.12.0.4-22023* (Mestrelab Research S.L.). All deuterated solvents were purchased from Deutero GmbH (Kastellaun, Germany).

HR-MS. Data-independent MS^E measurements were performed with a Vion IMS QToF (Waters) with an ESI source in positive sensitivity mode. Masses were detected with 0.2 s per scan and leucine enkephalin being injected as a reference mass every minute. Used parameters: capillary voltage 3.0 kV, sample cone voltage 40 V, source offset voltage 80 V, cone gas flow 50 L/h, desolvation gas flow 800 L/h, source temperature 120°C, desolvation temperature 350°C, collision gas N_2.

HPLC. HPLC was performed using a Knauer Smartline setup with a two-wavelength detector and a dynamic mixing chamber with reversed-phase chromatography columns. As eluents, MiliQ- water with 0.1% TFA (solvent A) and acetonitrile with 0.1% TFA (solvent B) were used. For the analytical HPLC, a Macherey-Nagel Nucleodur C4 Pyramid (5 µm; 125 x 4.6 mm) with a flow of 1 ml/min was used. For semi-preparative HPLC, a Macherey-Nagel Nucleodur 100-5 C18ec (5 µm; 125 x 10 mm) with a flow of 5 mL/min was used. The gradient was individually adjusted to achieve an optimal separation for every compound as indicated in the respective procedures.

UV-vis spectroscopy. UV-vis spectra were taken on a Jasco V-670 spectrophotometer. A baseline correction was performed with the corresponding blank before each spectrum was taken.

Fluorescent spectroscopy. Fluorescence spectroscopic measurements were made using a Jasco FP-8300 spectrofluorometer. The emission and excitation slit widths were fixed at 5 nm unless otherwise specified.

Cell culture. Ovcar-3 cells were maintained in RPMI-1640 medium (Invitrogen), A549 cells were maintained in high glucose (4.5 g/L) Dulbecco's Modified Eagle's medium (Invitrogen. Alle employed culture media were supplemented with 10% fetal bovine serum (Gibco), 2% penicillin/streptomycin (Sigma Aldrich), 1%GlutaMAX™-I (100x, Gibco), and 1% sodium pyruvate (100 mM, Gibco).
Cells were kept under a humidified atmosphere of 10% CO_2 and 90% air at 37°C. Cells were split when they reached 90% confluency. All employed cell lines were sourced from the German Collection of Microorganisms and Cell Cultures (Leibnitz Institute, DSMZ).

Cell viability studies and MTT assays. Cells were harvested and plated on a 96-well plate in 100 µL growth medium at a density of 1500 cells per well. The cells were left to grow for 24 h at 37°C under a humidified atmosphere with 10% CO_2. The next day, 100 µL of growth medium containing appropriate concentrations of the investigated compound were added to each well. As a control, the first row of wells was treated with 100 µL medium containing only the vehicle (1% DMSO); all wells contained a final concentration of 0.5% DMSO for the duration of the treatment. After incubation with the compound for 72 h, 50 µL of MTT solution (2.5 mg/mL 3-(4,5-dimethylthiazol-2-yl)-2,5-diphenyltetrazolium bromide dye in PBS buffer) was added to each well. After an additional incubation for 2 h, the supernatant was aspired off and 200 µL of DMSO was added to each well to solubilize the precipitated formazan dye. The produced formazan dye was quantified by measuring the optical density of the solution at 550 nm with a Tecan Infinite M Nano+ plate reader. A baseline correction was performed by subtracting a reference absorbance at 620 nm for each well. From the mean of the corrected absorbances for each set of equivalent wells, a dose-response curve was produced, which was then normalized to the wells containing untreated cells to allow for plate-to-plate comparison. The resulting dose-response curves were subjected to non-linear regression analysis performed in Microsoft Excel (version 16.64) to determine IC_{50} via the Hill-Langmuir model. Data is shown as the mean of three replicate experiments and error bars represent the standard deviation at each datum point.

2. Synthesis

2.1. Summary Synthesis Scheme

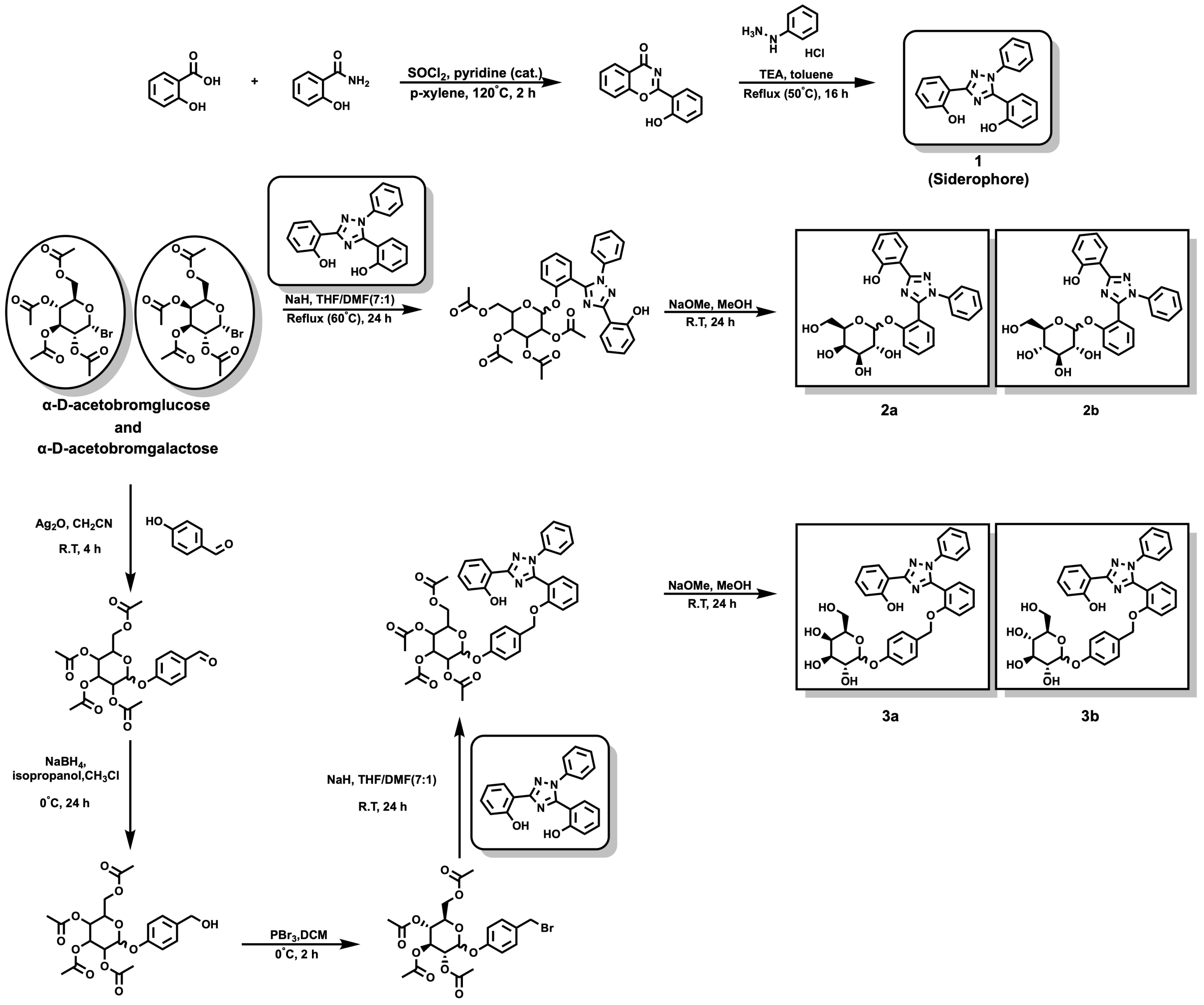

Scheme S1: Summary scheme of all syntheses relevant to the present study.

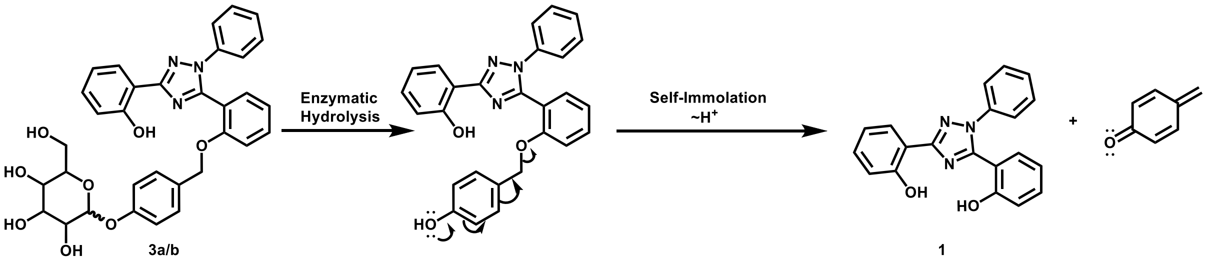

Scheme S2: Putative mechanism for the liberation **1** from the linker-based prodrugs **3a** and **3b**, respectively.

2.2. Oxazinone Intermediate

2-(2-hydroxyphenyl)-4a,8a-dihydro-4*H*-benzo[*e*][1,3]oxazin-4-one

This synthesis was adopted from the literature.[1]

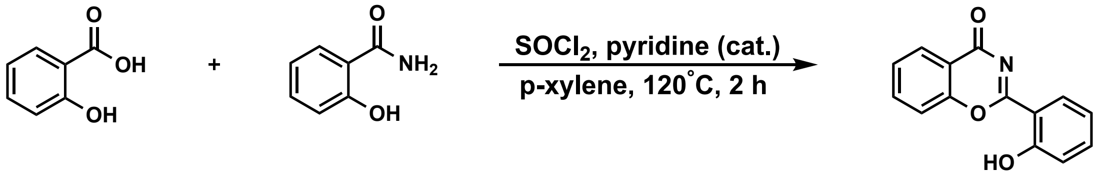

In a 500 ml round bottom flask equipped with a reflux condenser, salicylic acid (20.00 g, 114.8 mmol, 1.1 equiv.), salicylamide (18.05 g, 131.64 mmol, 1.0 equiv.), and pyridine (0.5 ml) were dissolved in p-xylene (100 ml) and the solution was heated to 80°C. $SOCl_2$ (6.68 ml, 92.15 mmol, 0.7 equiv.) was added dropwise over the course of 5 min. The reaction mixture was heated further to 120°C, stirred for 1 h, and then another aliquot of $SOCl_2$ (6.68 ml, 92.15 mmol, 0.7 equiv.) was added dropwise over the course of 5 min. The reaction was stirred for another 1 h at 120°C. After cooling, the volatiles were removed from the reaction mixture under reduced pressure, and to the residue was added ethanol (50 mL) and acetic acid (1 mL). The resulting suspension was cooled to 4°C in a refrigerator for 10 min and the precipitate was filtered off, washed with cold ethanol (three times, 50 mL each), and dried under vacuum to yield the product as a yellow-green powder (23 g, 73%).

^{1}H-NMR (300 MHz, $CDCl_3$):

δ 8.22-8.19 (dd, 1 H, Ar*H*), 8.12-8.09 (dd, 1 H, Ar*H*), 7.83-7.77 (ddd, 1 H, Ar*H*), 7.56-7.49 (m, 3 H, Ar*H*), 7.09-7.06 (dd, 1 H, Ar*H*), 7.02-6.96 (ddd, 1 H, Ar*H*)

---

^{13}C-NMR (75 MHz, $CDCl_3$):

δ 165.3, 164.1, 163.2, 154.2, 136.9, 135.8, 128.7, 128.0, 127.3, 119.5, 118.9, 118.3, 117.0, 111.3

2.3. 1

2-(5-(2-hydroxyphenyl)-1-phenyl-1H-1,2,4-triazol-3-yl)phenol

This synthesis was adopted from the literature.[1]

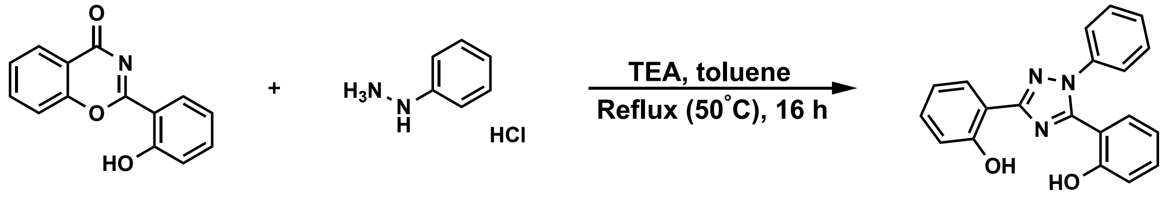

In a 500 mL round bottom flask, phenylhydrazine hydrochloride (14.50 g, 100.32 mmol, 1.2 equiv.), and triethylamine (8.40 g, 83.60 mmol, 1 equiv.) were added to toluene (150 mL) and heated to 50°C under stirring. 2-(2-hydroxyphenyl)-4H-benzo[e][1,3]oxazin-4-one (20.00 g, 83.60 mmol, 1.0 equiv.) was added at once through a solid addition funnel and the reaction was stirred at 50°C for 4 h, then slowly cooled to room temperature under stirring, and then continued to stir for another 12 h at room temperature (r.t). After the reaction was finished, the solvent was removed under reduced pressure. The crude solid product was dispensed in ethyl acetate (50 mL) and washed twice with 0.1 M HCl solution(100 mL each). The organic phase was concentrated under reduced pressure, and the residue was washed first with methanol (four times with 50 mL each) and then with cold acetone (one time, 20 mL; careful in order not to dissolve the product). After drying under vacuum, the product **1** was obtained as a pale-yellow powder (10.88 g, 40%).

^{1}H-NMR (300 MHz, $CDCL_3$):
δ 8.15-8.13 (dd, 1 H, Ar*H*), 7.60-7.58 (m, 3 H, Ar*H*), 7.53-7.50 (m, 2 H, Ar*H*), 7.40-7.30 (m, 2 H, Ar*H*), 7.15-7.12 (dd, 1 H, Ar*H*), 7.09-7.02 (m, 2 H, Ar*H*), 6.93-6.91 (dd, 1 H, Ar*H*), 6.65-6.61 (m, 1 H, Ar*H*).

^{13}C-NMR (75 MHz, $CDCl_3$):
δ 159.2, 158.3, 156.6, 152.1, 138.3, 132.9, 131.8, 130.4, 130.1, 127.7, 125.5, 126.5, 119.9, 118.9, 118.4, 117.2, 113.5, 110.2.

HR-MS (ESI): $C_{20}H_{15}N_3O_2$

Calculated ($[M+H]^+$): 330.1242

Found: 330.1232

2.4. Galactose intermediate 1

(2R,3S,4S,5R)-2-(acetoxymethyl)-6-(2-(3-(2-hydroxyphenyl)-1-phenyl-1H-1,2,4-triazol-5-yl)phenoxy)tetrahydro-2H-pyran-3,4,5-triyl triacetate

This synthesis was adopted from the literature.[2]

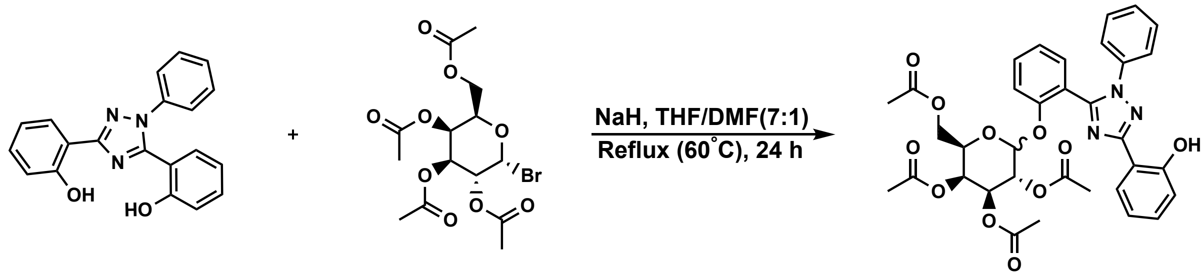

In a 50 mL schlenk flask, sodium hydride (163.38 mg, 4.86 mmol, 2.0 equiv.) was dissoled in THF (14 mL) at 0°C. Compound **1** (881.1 mg, 2.67 mmol, 1.1 equiv.) was dissolved in THF/DMF (7/3 v/v, 20 mL) and added dropwise to the mixture over the course of 10 min. After warming the mixture to room temperature, acetobromo-α-D-glucose (1.00 g, 2.43 mmol, 1.0 equiv.) dissolved in THF (14 mL) was added dropwise over the course of 10 min. The reaction mixture was then refluxed overnight. The crude reaction mixture was washed with a saturated, aqueous NH_4Cl solution (three times) and once with brine. The organic phase was then concentrated under reduced pressure and the crude intermediate was purified via flash column chromatography on silica gel (50:50 V/V% n-heptane:EtOAc) to yield a viscous, yellow liquid containing the **acetyl-protected glucose intermediate 1** (400 mg, 25%).

^{1}H-NMR (300 MHz, DCM):

δ 8.17 (s, 1 H, Ar*H*), 7.62-7.60 (m, 1 H, Ar*H*), 7.53 (m, 1 H, Ar*H*), 7.42 (s, 5 H, Ar*H*), 7.35-7.25 (m, 2 H, Ar*H*), 7.12-7.0 (m, 3 H, Ar*H*), 5.39-5.36 (m, 2 H, C-*H*), 5.12 (s, 1 H, C-*H*), 4.97 (m, 2 H, C-*H*),4.12-4.05(m,2 H, C-H), 2.15 (s, 3 H, C*H₃*), 2.03 (s, 3 H, C*H₃*) 2.0 (s, 3 H, C*H₃*) 1.91 (s, 3 H, C*H₃*)

---

^{13}C-NMR (75 MHz, DCM):

δ 170.3, 169.1, 161.4, 158.4, 157.6, 150.2, 149.3, 138.0, 132.2, 131.7, 131.6, 131.2, 130.3, 129.8, 129.3, 127.8, 127.2, 126.7, 126.4, 124.8, 124.3, 123.8, 121.4, 119.9, 119.0, 118.3, 117.6, 114.3, 32.3, 29.5, 23.1, 21.6, 14.3

2.5. 2a

(2R,3R,4S,5R)-2-(hydroxymethyl)-6-(2-(3-(2-hydroxyphenyl)-1-phenyl-1H-1,2,4-triazol-5 yl)phenoxy)tetrahydro-2H-pyran-3,4,5-triol

This synthesis was adopted from the literature.[2]

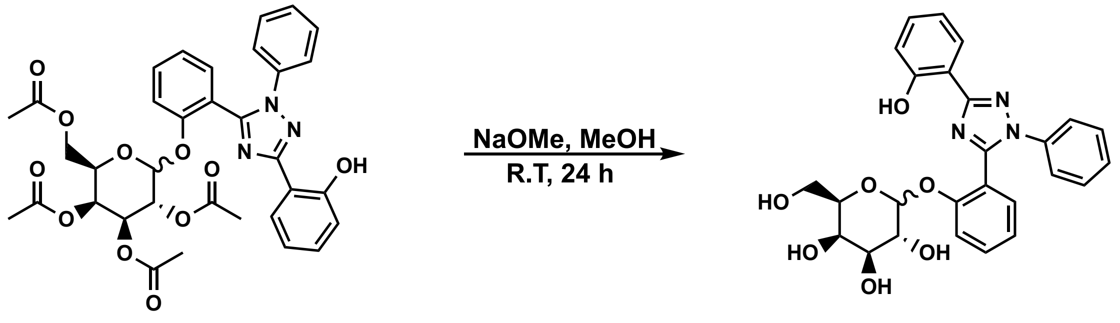

In 20 mL flask, **galactose intermediate 1** (370 mg, 0.56 mmol, 1 equiv.) was dissolved in methanol (10 mL). Then, sodium methoxide (154 mg, 2.80 mmol, 5 equiv.) was added and the reaction was stirred at r.t. for 24 h. The solvent was then removed under reduced pressure and the crude product was purified via flash chromatography on silica gel (90/10 V/V% EtOAc:MeOH) to yield (70 mg, 26%) the product as a fluffy white powder.

^{1}H-NMR (300 MHz, MeOD-d$_4$):
δ 8.11-8.08 (dd, 1 H, Ar*H*), 7.54-7.45 (m, 3 H, Ar*H*), 7.44-7.39 (m, 3 H, Ar*H*), 7.38-7.30 (m, 3 H, Ar*H*), 7.12-7.07 (ddd, 1 H, Ar*H*), 7.01-6.94 (m, 2 H, Ar*H*), 4.91-4.89 (d, 1 H, C-H), 4.14-4.07 (m, 1 H, C-*H*), 3.85-3.83 (dd, 1 H, C-*H*), 3.70-3.68 (m, 2 H, C-*H*), 3.65-3.59 (m, 2 H, C-*H*), 3.54-3.49 (m, 1 H, C-*H*)

---

^{13}C-NMR (75 MHz, MeOD-d$_4$):
δ 161.9, 158.0, 157.2, 138.9, 133.5, 132.2, 130.3, 130.0, 128.1, 125.9, 123.6, 120.6, 119.4, 118.0, 117.9, 115.2, 103.6, 77.09, 74.85, 72.0, 70.0, 62.2, 61.5.

HR-MS (ESI): $C_{26}H_{25}N_3O_7$

Calculated ([M+Na]$^+$): 514.1590

Found: 514.1581

2.6. Glucose intermediate 1

(2R,3S,4S,5R)-2-(acetoxymethyl)-6-(2-(3-(2-hydroxyphenyl)-1-phenyl-1H-1,2,4-triazol-5-yl)phenoxy)tetrahydro-2H-pyran-3,4,5-triyl triacetate

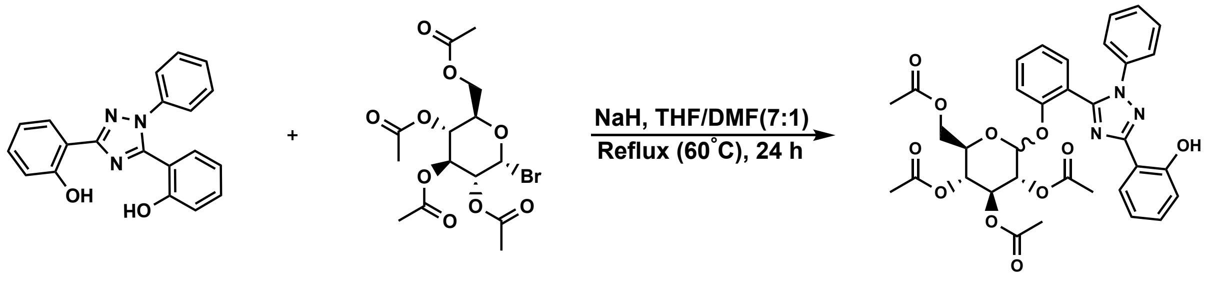

In a 50 mL schlenk flask, sodium hydride (82.0 mg, 2.43 mmol, 2 equiv.) was dissoled in THF (7 mL) at 0°C. **1** (362.29 mg, 1.21 mmol, 1.0 equiv.) was dissolved in THF/DMF (7/3 v/v, 10 mL) and added dropwise to the mixture over the course of 10 min. After warming the mixture to room temperature, acetobromo-α-D-glucose (500 mg, 1.21 mmol, 1 equiv.) dissolved in THF (7 mL) was added dropwise over the course of 10 min. The reaction mixture was then refluxed overnight. The crude reaction mixture was washed with saturated NH_4Cl aqueous solution (three times) and once with brine. The organic phase was then concentrated under reduced pressure and the crude intermediate was purified via flash column chromatography on silica gel (50:50 V/V% n-heptane:EtOAc) to yield a viscous, yellow liquid of the acetyl-protected glucose intermediate 1 (45 mg, 5%).

^{1}H-NMR (300 MHz, DCM):
δ 8.17 (s, 1 H, Ar*H*), 7.62-7.60 (m, 1 H, Ar*H*), 7.53 (m, 1 H, Ar*H*), 7.42 (s, 5 H, Ar*H*), 7.35-7.25 (m, 2 H, Ar*H*), 7.12-7.0 (m, 3 H, Ar*H*), 5.39-5.36 (m, 2 H, C-*H*), 5.12 (s, 1 H, C-*H*), 4.97 (m, 2 H, C-*H*), 2.15 (s, 3 H, CH_3), 2.03 (s, 3 H, CH_3) 2.0 (s, 3 H, CH_3) 1.91 (s, 3 H, CH_3)

---

^{13}C-NMR (75 MHz, DCM):
δ 170.3, 169.1, 161.4, 158.4, 157.6, 150.2, 149.3, 138.0, 132.2, 131.7, 131.6, 131.2, 130.3, 129.8, 129.3, 127.8, 127.2, 126.7, 126.4, 124.8, 124.3, 123.8, 121.4, 119.9, 119.0, 118.3, 117.6, 114.3, 32.3, 29.5, 23.1, 21.6, 14.3

2.7. 2b

(2R,3S,4S,5R)-2-(hydroxymethyl)-6-(2-(3-(2-hydroxyphenyl)-1-phenyl-1H-1,2,4-triazol-5 yl)phenoxy)tetrahydro-2H-pyran-3,4,5-triol

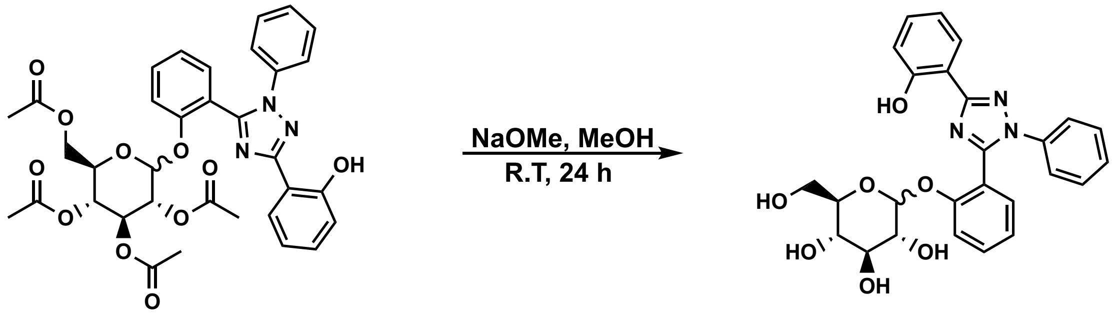

In 20 mL flask, **glucose intermediate 1** (30.0 mg, 0.04 mmol, 1 equiv.) was dissolved in methanol (4 mL). Then, sodium methoxide (10.80 mg, 0.20 mmol, 5 equiv.) was added and the reaction was stirred at r.t. for 24 h. The solvent was then removed under reduced pressure and the crude product was purified via flash chromatography on silica gel (90/10 V/V% EtOAc:MeOH) to yield (5 mg, 25%) the product as a fluffy white powder.

^{1}H-NMR (300 MHz, MeOD-d$_4$):
δ 8.11-8.08 (dd, 1 H, Ar*H*), 7.55-7.52 (m, 1 H, Ar*H*), 7.50-7.47 (m, 2 H, Ar*H*), 7.46-7.37 (m, 5 H, Ar*H*), 7.33-7.31 (m, 1 H, Ar*H*), 7.15-7.10 (m, 1 H, Ar*H*), 7.01-6.95 (m, 2 H, Ar*H*), 4.95-4.92 (d, 1 H, C-*H*), 3.86-3.81 (dd, 1 H, C-*H*), 3.61-3.55 (m, 1 H, C-*H*), 3.41-3.35 (m, 2 H, C-*H*), 3.26-3.19 (m, 2 H, C-*H*)

---

^{13}C-NMR (75 MHz, MeOD-d$_4$):
δ 158.0, 156.8, 139.1, 133.6, 132.39, 132.31, 130.38, 130.30, 130.0, 128.1, 125.7, 125.6, 123.6, 120.6, 119.2, 118.0, 117.5, 102.4, 78.2, 77.9, 74.5, 71.3, 62.6

HR-MS (ESI): $C_{26}H_{25}N_3O_7$

Calculated ($[M+Na]^+$): 514.1590

Found: 514.1580

2.8. Galactose linker intermediate 1
(2*R*,3*S*,4*S*,5*R*)-2-(acetoxymethyl)-6-(4-formylphenoxy)tetrahydro-2*H*-pyran-3,4,5-triyl triacetate

This synthesis was adopted from the literature.[3]

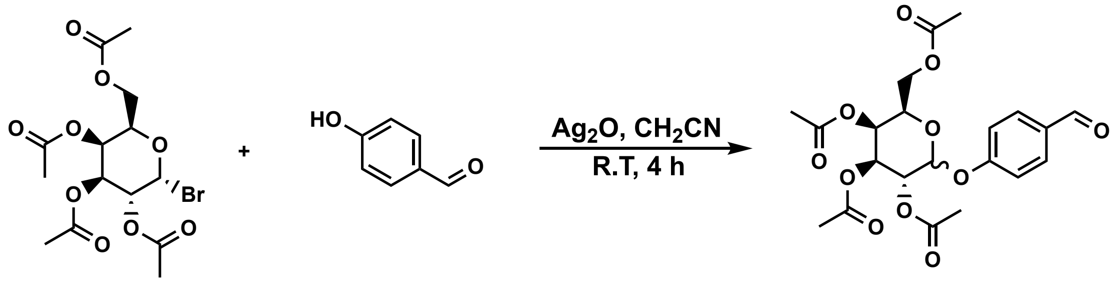

In a 100 mL Schlenk flask, 4-hydroxybenzaldehyde (326.6 mg, 2.67 mmol, 1.1 equiv.) was dissolved in dry CH_3CN (30 ml). To this stirring solution, acetobromo-α-D-galactose (1.00 g, 2.40 mmol, 1 equiv.), which had been dissolved in dry CH_3CN (40 ml) containing dry Ag_2O (1.10 g, 4.86 mmol, 2 equiv.), was added. After stirring the reaction for 4 h at room temperature, the mixture was filtered to remove the Ag salts, and the solvent was removed under reduced pressure. The crude product was purified via flash chromatography on silica gel (70/30 V/V% *n*- heptane : EtOAc) to yield (824.2 mg, 75%) the product as a white solid.

^{1}H-NMR (300 MHz, CDCL$_3$):

δ 9.91 (s, 1 H, RCO-*H*), 7.85-7.82 (d, 2 H, Ar*H*), 7.11-7.09 (d, 2 H, Ar*H*), 5.54-5.46 (m, 2 H, C-*H*), 5.18-5.10 (m, 2 H, C-*H*), 4.22-4.11 (m, 3 H, C-*H*), 2.17 (s, 3 H, C*H*$_3$), 2.05 (s, 6 H, C*H*$_3$), 2.0 (s, 3 H, C*H*$_3$)

^{13}C-NMR (75 MHz, CDCL$_3$):

δ 190.8, 170.4, 170.3, 170.2, 169.4, 161.4, 131.9, 116.9, 98.7, 71.4, 70.8, 68.5, 66.9, 61.5, 60.5, 21.1, 20.8, 20.8, 20.7, 20.7, 14.3.

2.9. Galactose linker intermediate 2
(2R,3S,4S,5R)-2-(acetoxymethyl)-6-(4-(hydroxymethyl)phenoxy)tetrahydro-2H-pyran-3,4,5-triyl triacetate

This synthesis was adopted from the literature.[3]

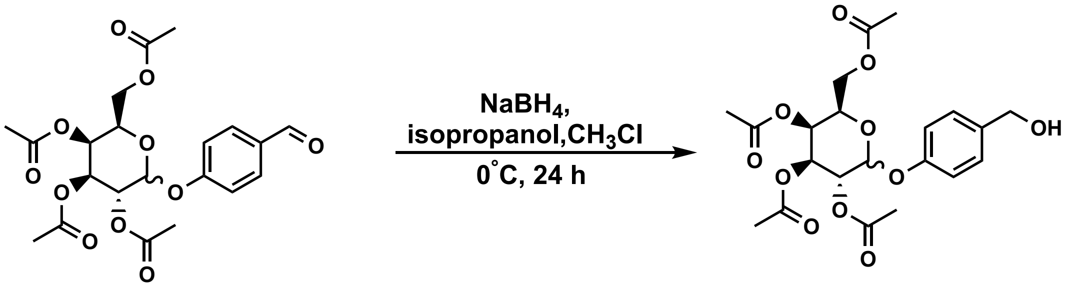

In 100 mL Schlenk flask, **galactose linker intermediate 1** (855 mg, 1.89 mmol, 1 equiv.) was dissolved in dry $CHCl_3$ (40 mL) and dry isopropanol (10 mL). With the help of a solid addition funnel, $NaBH_4$ (121.50 mg, 3.21 mmol, 1.7 equiv.) was added and the reaction was stirred overnight at 4°C. The crude reaction mixture was washed with water (three times), and the organic phase was removed under reduced pressure. The product (350 mg, 40%) was obtained as a white solid.

^{1}H-NMR (300 MHz, CDCL$_3$):

δ 7.32-7.28 (d, 2 H, Ar*H*), 7.01-6.97 (d, 2 H, Ar*H*), 5.49-5.44 (m, 2 H, C-*H*), 5.14-5.01 (m, 2 H, C-*H*), 4.64 (s, 2 H, C-*H*), 4.24-4.05 (m, 3 H, C-*H*), 2.18 (s, 3 H, C*H3*), 2.06, 2.05 (d, 6 H, C*H3*) 2.01 (s, 3 H, C*H3*)

---

^{13}C-NMR (75 MHz, CDCL$_3$):

δ 170.7, 170.4, 169.5, 169.4, 156.5, 149.7, 136.1, 130.1, 128.6, 117.2, 99.3, 72.8, 72.1, 71.3, 68.4, 64.9, 62.0, 20.8, 20.76, 20.75, 20.73.

2.10. Galactose linker intermediate 3

(2*R*,3*S*,4*S*,5*R*)-2-(acetoxymethyl)-6-(4-(bromomethyl)phenoxy)tetrahydro-2*H*-pyran- 3,4,5-triyl triacetate

This synthesis was adopted from the literature.[3]

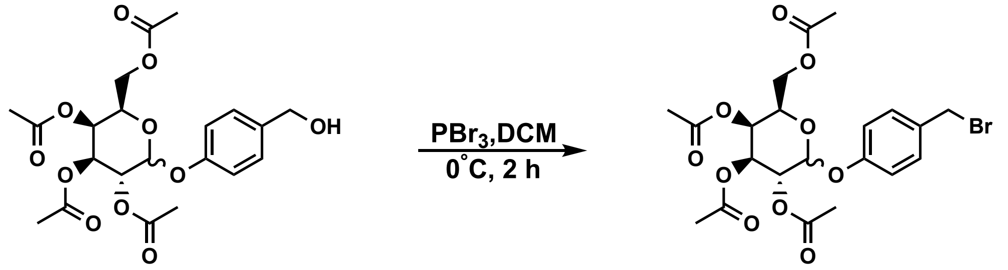

In 50 mL Schlenk flask, **galactose linker intermediate 2** (350 mg, 0.77 mmol, 1 equiv.) was dissolved in dry DCM (20 mL). The reaction mixture was then brought to 0°C and PBr_3 (230 mg, 0.85 mmol, 1.1 equiv.) was added and stirred for 2 h. The crude reaction mixture was washed with saturated $NaHCO_3$ aqueous solution (three times) and brine once. The organic phase was concentrated under reduced pressure and dried under vacuum. The product (175 mg, 43%) was obtained as a white oil.

^{1}H-NMR (300 MHz, $CDCL_3$):

δ 7.33-7.31 (d, 2 H, Ar*H*), 6.97-6.94 (d, 2 H, Ar*H*), 5.50-5.44 (m, 2 H, C-*H*), 5.12-5.02 (m, 2 H, C-*H*), 4.47 (s, 2 H, C-*H*), 4.25-4.06 (m, 3 H, C-*H*), 2.17 (s, 3 H, C*H₃*), 2.05 (d, 6 H, C*H₃*), 2.00 (s, 3 H, C*H₃*)

---

^{13}C-NMR (75 MHz, $CDCL_3$):

δ 170.5, 170.3, 170.2, 169.5, 156.9, 132.9, 130.6, 130.1, 129.8, 117.2, 99.5, 71.2, 70.9, 68.7, 67.0, 61.5, 33.2, 21.1, 20.8, 20.8, 20.7.

2.11. Galactose linker intermediate 4
(2R,3S,4S,5R)-2-(acetoxymethyl)-6-(4-((2-(3-(2-hydroxyphenyl)-1-phenyl-1H-1,2,4- triazol-5-yl)phenoxy)methyl)phenoxy)tetrahydro-2H-pyran-3,4,5-triyl triacetate

This synthesis was adopted from the literature.[3]

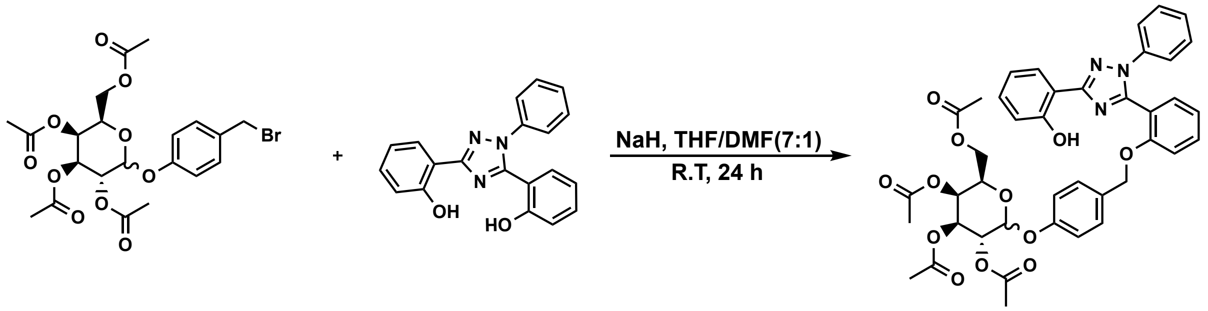

In a 50 mL Schlenk flask, sodium hydride (14.50 mg, 0.66 mmol, 2 equiv.) was dissolved in THF (1 mL) at 0°C. To the mixture was added dropwise over the course of 10 min **1** (108.7 mg, 0.33 mmol, 1 equiv.), which had been dissolved prior in THF/DMF (7/3 v/v, 1.5 mL). After warming the mixture to room temperature, **galactose linker intermediate 3** (175 mg, 0.33 mmol, 1 equiv.) dissolved in THF (2 mL) was added dropwise over the course of 10 min. The reaction mixture was then stirred at r.t. for 42 h. The crude reaction mixture was washed with saturated NH_4Cl aqueous solution (three times) and brine once, the organic phase was then concentrated under reduced pressure, and the crude intermediate was purified via flash column chromatography on silica gel (50:50 V/V% *n*-heptane : EtOAc) to yield a white solid containing the acetyl-protected **galactose linker intermediate 4** (116 mg, 45%).

^{1}H-NMR (300 MHz, CDCL$_3$):

δ 8.20-8.17 (dd, 1 H, Ar*H*), 7.63-7.60 (dd, 1 H, Ar*H*), 7.47-7.42 (dd, 1 H, Ar*H*), 7.36-7.31 (m, 2 H, Ar*H*), 7.29-7.28 (m, 4 H, Ar*H*), 7.14-7.05 (m, 2 H, Ar*H*), 7.00-6.95 (m, 1 H, Ar*H*), 6.91-6.85 (m, 5 H, Ar*H*), 5.49-5.44 (m, 2 H, C-*H*), 5.12-4.98 (m, 2 H, C-*H*), 4.67 (s, 2 H, C-*H*), 4.20-4.11 (m, 3 H, C-*H*), 2.18 (s, 3 H, C*H$_3$*), 2.06 (s, 6 H, C*H$_3$*), 2.03 (d, 3 H, C*H$_3$*), 2.01 (s, 3 H, C*H$_3$*)

---

^{13}C-NMR (75 MHz, CDCL$_3$):

δ 170.5, 170.4, 170.3, 169.5, 160.8, 157.2, 156.7, 156.2, 151.1, 138.4, 132.5, 131.8, 131.4, 131.0, 129.1, 128.8, 128.3, 127.1, 123.4, 121.6, 119.6, 117.4, 116.9, 114.1, 113.1, 99.6, 71.1, 70.9, 70.0, 68.7, 67.0, 61.5, 60.5, 32.0, 29.2, 22.8, 21.2, 20.9, 20.8, 20.7, 14.3.

2.12. 3a
(2R,3R,4S,5R)-2-(hydroxymethyl)-6-(4-((2-(3-(2-hydroxyphenyl)-1-phenyl-1H-1,2,4- triazol-5-yl)phenoxy)methyl)phenoxy)tetrahydro-2H-pyran-3,4,5-triol

This synthesis was adopted from the literature.[3]

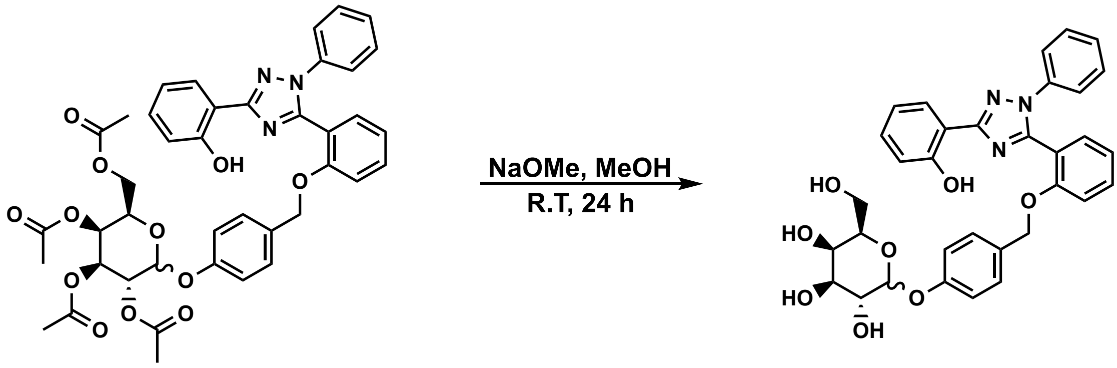

In a 20 mL round bottom flask, **galactose linker intermediate 4** (20.0 mg, 0.02 mmol, 1 equiv.) was dissolved in MeOH (4 mL). Then, sodium methoxide (3.20 mg, 0.06 mmol, 3 equiv.) was added and the reaction was stirred at 0°C for 24 h. The solvent was then removed under reduced pressure. The crude product was purified via flash chromatography on silica (90/10 V/V% EtOAc : MeOH) to yield (5 mg, 45%) the product as a fluffy white powder.

^{1}H-NMR (300 MHz, MeOD-*d*$_4$):
δ 8.09-8.06 (dd, 1 H, Ar*H*), 7.61-7.58 (dd, 1 H, Ar*H*), 7.51-7.45 (m, 1 H, Ar*H*), 7.39-7.29 (m, 4 H, Ar*H*), 7.26-7.23 (m, 2 H, Ar*H*), 7.15-7.09 (m, 1 H, Ar*H*), 7.05-6.95 (m, 5 H, Ar*H*), 6.92-6.89 (m, 2 H, Ar*H*), 4.72 (s, 2 H, C-*H*),4.13-4.06 (m, 1 H, C-*H*), 3.91-3.86 (dd, 1 H, C-*H*), 3.72-3.66 (m, 1 H, C-*H*), 3.45-3.41 (m, 4 H, C-*H*),

^{13}C-NMR (75 MHz, MeOD-*d*$_4$):
δ 172.3, 161.9, 158.8, 158.1, 157.6, 153.1, 139.3, 133.7, 132.5, 132.3, 131.4, 130.2, 129.7, 128.0, 124.6, 122.2, 120.6, 118.6, 118.1, 117.5, 115.1, 114.1, 102.2, 78.1, 77.9, 74.9, 71.3, 71.0, 62.5, 61.5.

HR-MS (ESI): $C_{33}H_{31}N_3O_8$

Calculated ([M+Na]$^+$): 620.1984

Found: 620.2008

2.13. Glucose linker intermediate 1
(2R,3R,4S,5R)-2-(acetoxymethyl)-6-(4-formylphenoxy)tetrahydro-2H-pyran-3,4,5-triyltriacetate

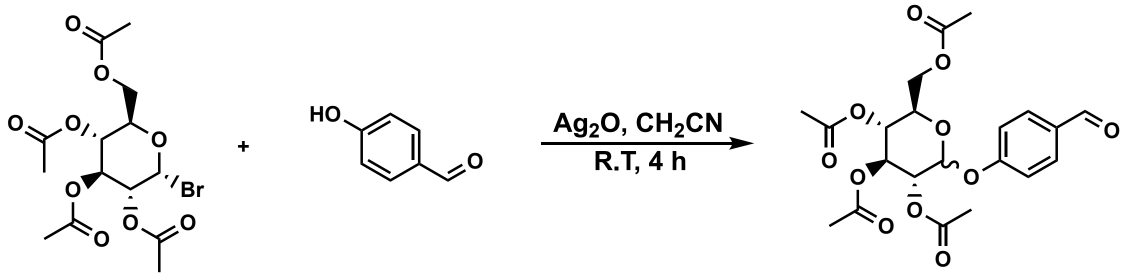

In a 100 mL Schlenk flask, 4-hydroxybenzaldehyde (326.60 mg, 2.67 mmol, 1.1 equiv.) was dissolved in dry CH_3CN (30 ml). To this stirring solution, acetobromo-α-D-glucose (1.00 g, 2.40 mmol, 1 equiv.) dissolved in dry CH_3CN (40 ml) containing dry Ag_2O (1.10 g, 4.86 mmol, 2 equiv.) was added. After stirring the reaction contents for 4 h at room temperature, the mixture was filtered to remove the Ag salts, and the solvent was removed under reduced pressure. The crude product was purified via flash chromatography on silica gel (70/30 V/V% *n*-heptane : EtOAc) to yield (587 mg, 53%) the product as a white solid.

^{1}H-NMR (300 MHz, CDCL$_3$):

δ 9.92 (s, 1 H, RCO-*H*), 7.86-7.84 (d, 2 H, Ar*H*), 7.11-7.08 (d, 2 H, Ar*H*), 5.33-5.30 (m, 2 H, C-*H*), 5.23-5.14 (m, 2 H, C-*H*), 4.32-4.26 (m, 1 H, C-*H*), 4.20-4.15 (m, 1 H, C-*H*), 3.95-3.89 (m, 1 H, C-*H*), 2.07 (s, 3 H, C*H$_3$*), 2.06 (s, 6 H, C*H$_3$*), 2.04 (s, 3 H, C*H$_3$*)

---

^{13}C-NMR (75 MHz, CDCL$_3$):

δ 190.8, 170.4, 170.3, 170.2, 169.4, 161.4, 131.9, 116.9, 98.7, 71.4, 70.8, 68.5, 66.9, 61.5, 60.5, 21.1, 20.8, 20.8, 20.7, 20.7, 14.3.

2.14. Glucose linker intermediate 2
(2R,3R,4S,5R)-2-(acetoxymethyl)-6-(4-(hydroxymethyl)phenoxy)tetrahydro-2H-pyran-3,4,5-triyl triacetate

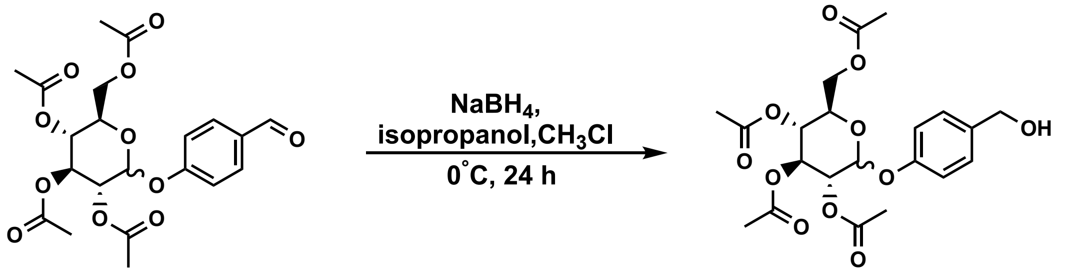

In a 100 mL Schlenk flask, **glucose linker intermediate 1** (580 mg, 1.28 mmol, 1 equiv.) was dissolved in dry $CHCl_3$ (40 mL) and dry isopropanol (10 mL). With the help of a solid addition funnel, $NaBH_4$ (82.00 mg, 2.17 mmol, 1.7 equiv.) was added and the reaction was stirred overnight at 4°C. The crude reaction mixture was washed with water (three times), and the organic phase was concentrated under reduced pressure. The product (437.60 mg, 75%) was obtained as a white solid.

^{1}H-NMR (300 MHz, CDCL$_3$):

δ 7.31-7.28 (d, 2 H, Ar*H*), 6.99-6.96 (d, 2 H, Ar*H*), 5.30-5.26 (m, 2 H, C-*H*), 5.19-5.13 (m, 1 H, C-*H*), 5.08-5.04 (m, 1 H, C-*H*), 4.64 (s, 2 H, C-*H*), 4.31-4.25 (m, 1 H, C-*H*), 4.19-4.14 (m, 1 H, C-*H*), 3.88-3.82 (m, 1 H, C-*H*), 2.07 (s, 3 H, C*H$_3$*), 2.05 (d, 3 H, C*H$_3$*), 2.04 (d, 3 H, C*H$_3$*), 2.03 (s, 3 H, C*H$_3$*)

^{13}C-NMR (75 MHz, CDCL$_3$):

δ 170.7 170.4, 169.5, 169.4, 156.5, 149.7, 136.1, 130.1, 128.6, 117.2, 99.3, 72.8, 72.1, 71.3, 68.4, 64.9, 62.0, 20.8, 20.76, 20.75, 20.73.

2.15. Glucose linker intermediate 3

(2R,3R,4S,5R)-2-(acetoxymethyl)-6-(4-(bromomethyl)phenoxy)tetrahydro-2H-pyran- 3,4,5-triyl triacetate

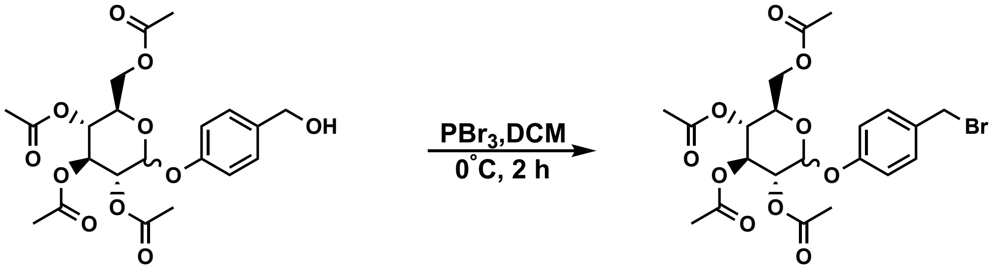

In a 50 mL Schlenk flask, **glucose linker intermediate 2** (430 mg, 0.946 mmol, 1 equiv.) was dissolved in dry DCM (20 mL). The reaction mixture was then cooled to 0°C and PBr_3 (281 mg, 1.04 mmol, 1.1 equiv.) was added and stirred for 2 h. The crude reaction mixture was washed with saturated aqueous $NaHCO_3$ solution (3 times) and brine once. The organic phase was concentrated under reduced pressure. The product (453 mg, 95%) was obtained as a white oil.

^{1}H-NMR (300 MHz, $CDCL_3$):

δ 7.34-7.31 (d, 2 H, Ar*H*), 6.96-6.94 (d, 2 H, Ar*H*), 5.30-5.26 (m, 2 H, C-*H*), 5.20-5.13 (m, 1 H, C-*H*), 5.10-5.07 (m, 1 H, C-*H*) 4.47 (s, 2 H, C-*H*), 4.31-4.25 (m, 1 H, C-*H*), 4.19-4.14 (m, 1 H, C-*H*), 3.89-3.83 (m, 1 H, C-*H*), 2.07 (s, 3 H, C*H₃*), 2.05 (d, 6 H, C*H₃*), 2.03 (s, 3 H, C*H₃*)

---

^{13}C-NMR (75 MHz, $CDCL_3$):

δ 170.5, 170.2, 169.4, 169.2, 156.7, 132.8, 130.4, 130.0, 117.1, 98.8, 89.8, 72.6, 72.1, 71.1, 68.2, 61.9, 33.1, 20.7, 20.6.

2.16. Glucose linker intermediate 4

(2R,3R,4S,5R)-2-(acetoxymethyl)-6-(4-((2-(3-(2-hydroxyphenyl)-1-phenyl-1H-1,2,4- triazol-5-yl)phenoxy)methyl)phenoxy)tetrahydro-2H-pyran-3,4,5-triyl triacetate

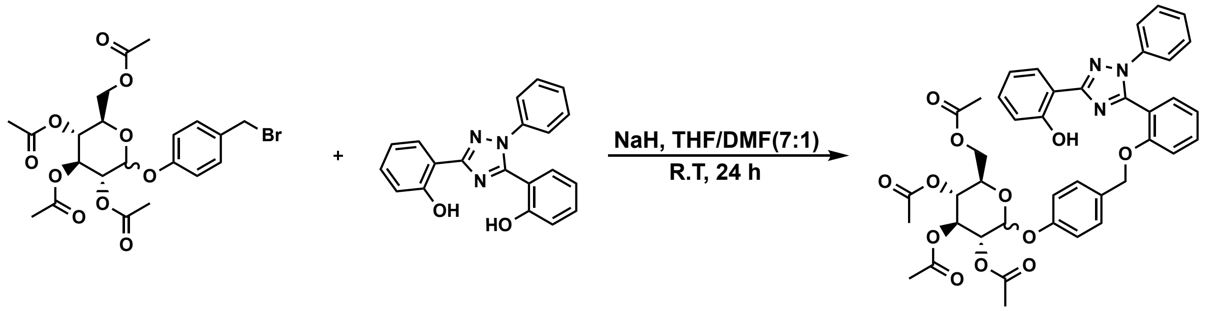

In a 50 mL Schlenk flask, sodium hydride (32 mg, 0.94 mmol, 1.1 equiv.) was dissolved in THF (2 mL) at 0°C. To the mixture, **1** (283.2 mg, 0.86 mmol, 1 equiv.) dissolved in THF/DMF (7/3 v/v, 3 mL) was added dropwise over the course of 10 min. After warming the mixture to room temperature, **glucose linker intermediate 3** (450 mg, 0.86 mmol, 1 equiv.) dissolved in THF (4 mL) was added dropwise for 10 min. The reaction mixture was then stirred at r.t. for 42 h. The crude reaction mixture was washed with saturated aqueous NH_4Cl solution (three times) and brine once. The organic phase was then concentrated under reduced pressure, and the crude intermediate was purified via flash column chromatography on silica gel (50:50 V/V% *n*-heptane : EtOAc) to yield a white solid containing the acetyl-protected **glucose linker intermediate 4** (363 mg, 55%).

^{1}H-NMR (300 MHz, $CDCL_3$):

δ 8.16-8.13 (dd, 1 H, Ar*H*), 7.59-7.56 (dd, 1 H, Ar*H*), 7.43-7.38 (m, 1 H, Ar*H*), 7.33-7.28 (m, 2 H, Ar*H*), 7.26-7.23 (m, 4 H, Ar*H*), 7.10-7.02 (m, 2 H, Ar*H*), 6,97-6.91 (m, 1 H, Ar*H*), 6.88-6.81 (m, 5 H, Ar*H*), 5.27-5.23 (m, 2 H, C-*H*), 5.13-5.00 (m, 2 H, C-*H*), 4.63 (s, 2 H, C-*H*), 4.29-4.23 (m, 1 H, C-*H*), 4.15-4.10 (m, 1 H, C-*H*) 3.84-43.78 (m, 1 H, C-*H*) 2.03 (s, 3 H, CH_3), 2.02 (s, 3 H, CH_3), 2.01 (s, 6 H, CH_3)

---

^{13}C-NMR (75 MHz, $CDCL_3$):

δ 170.6, 170.3, 169.5, 169.4, 160.9, 157.1, 156.5, 156.0, 151.1, 138.3, 132.4, 131.7, 131.2, 131.0, 129.0, 128.6, 128.2, 126.9, 123.3, 121.4, 119.5, 117.8, 117.3, 116.8, 114.1, 113.0, 99.0, 72.7, 72.1, 71.2, 69.8, 68.3, 65.8, 61.9, 50.7, 21.1, 20.7, 20.7, 20.6.

2.17. 3b

(2R,3S,4S,5R)-2-(hydroxymethyl)-6-(4-((2-(3-(2-hydroxyphenyl)-1-phenyl-1H-1,2,4- triazol-5-yl)phenoxy)methyl)phenoxy)tetrahydro-2H-pyran-3,4,5-triol

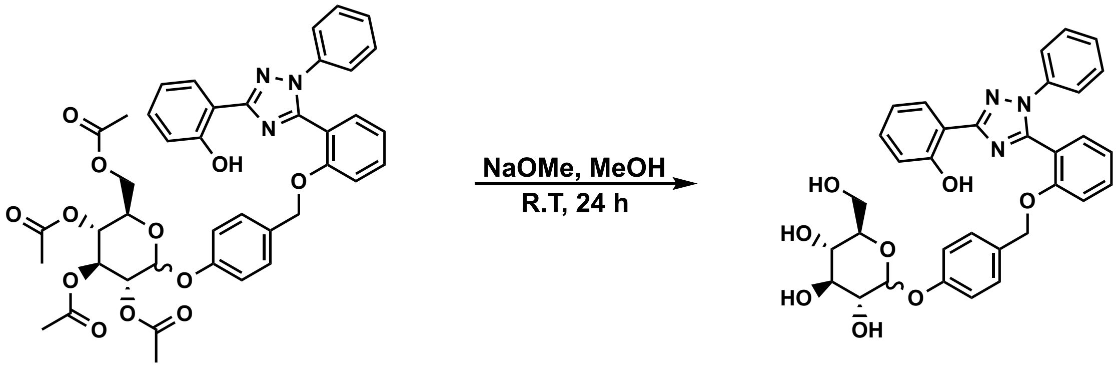

In 20 mL flask, **glucose linker intermediate 4** (360 mg, 0.47 mmol, 1 equiv.) was dissolved in methanol (20 mL). Then, sodium methoxide (101.50 mg, 1.88 mmol, 4 equiv.) was added and the reaction was stirred at 0°C for 24 h. The solvent was then removed under reduced pressure. The crude product was purified via flash chromatography on silica gel (90/10 V/V% EtOAc:MeOH) to yield (56 mg, 20%) of the product as a fluffy white powder.

^{1}H-NMR (300 MHz, MeOD-*d*$_4$):

δ 8.09-8.06 (dd, 1 H, Ar*H*), 7.61-7.58 (dd, 1 H, Ar*H*), 7.51-7.45 (m, 1 H, Ar*H*), 7.39-7.29 (m, 4 H, Ar*H*), 7.26-7.23 (m, 2 H, Ar*H*), 7.15-7.09 (m, 1 H, Ar*H*), 7.05-6.95 (m, 5 H, Ar*H*), 6.92-6.89 (m, 2 H, Ar*H*), 4.72 (s, 2 H, C-*H*),4.13-4.06 (m, 1 H, C-*H*), 3.91-3.86 (dd, 1 H, C-*H*), 3.72-3.66 (m, 1 H, C-*H*), 3.45-3.41 (m, 4 H, C-*H*),

^{13}C-NMR (75 MHz, MeOD-*d*$_4$):

δ 172.99, 161.93, 158.84, 158.12, 157,57, 153.07, 139.34, 133.69, 132.52, 132.31, 131.42 130.23, 129.96, 129.67, 127.99, 124.61, 122.20, 120.63, 118.57, 118.07, 117.55, 115.11, 114.15, 102.18, 78.11, 77.94, 74.88, 71.35, 70.95, 62.49, 61.53, 20.86, 14.46

HR-MS (ESI): $C_{33}H_{31}N_3O_8$

Calculated ([M+Na]$^+$): 620.1984

Found: 620.2022

3. ^{1}H-NMR, ^{13}C-NMR, and HRMS

3.1. **Oxazinone Intermediate**

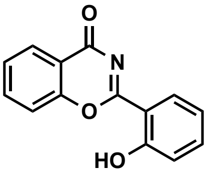

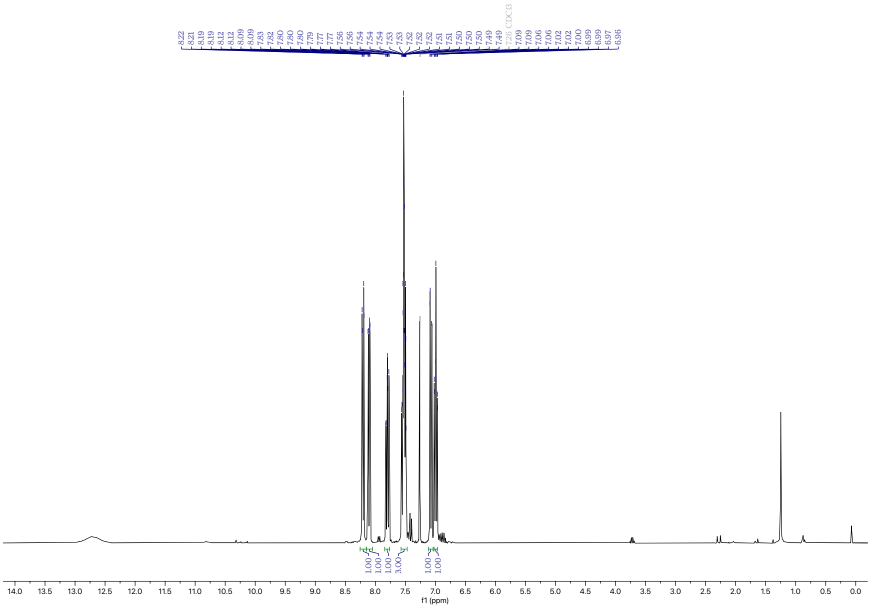

^{1}H-NMR spectrum of **Oxazinone Intermediate** (300 MHz, $CDCl_3$)

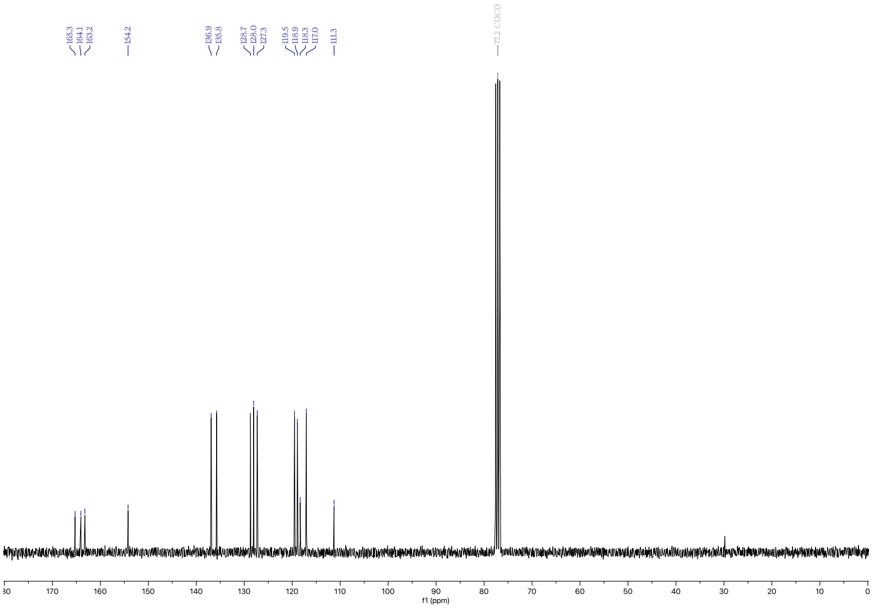

^{13}C-NMR spectrum of **Oxazinone Intermediate** (75 MHz, $CDCl_3$)

3.2. **1**

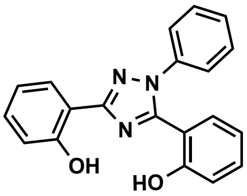

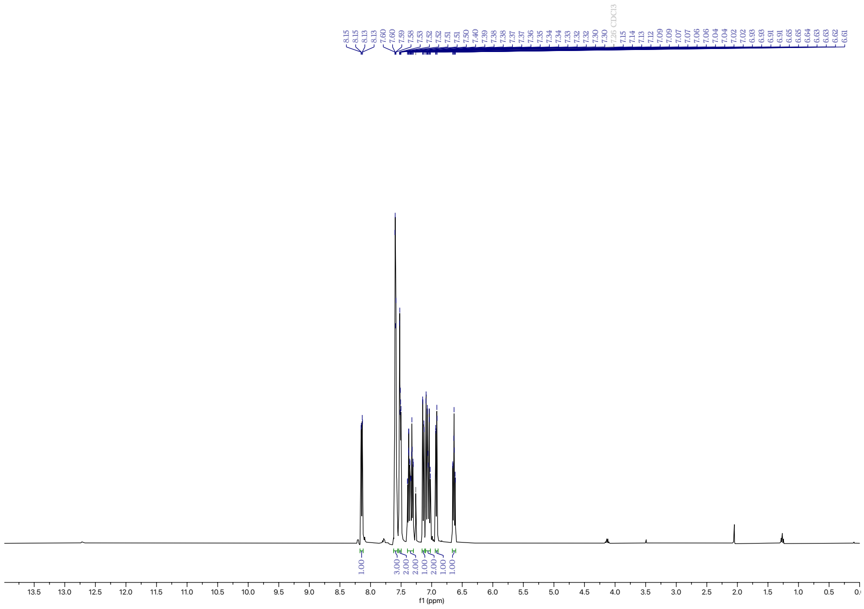

^{1}H-NMR spectrum of **1** (300 MHz, $CDCl_3$)

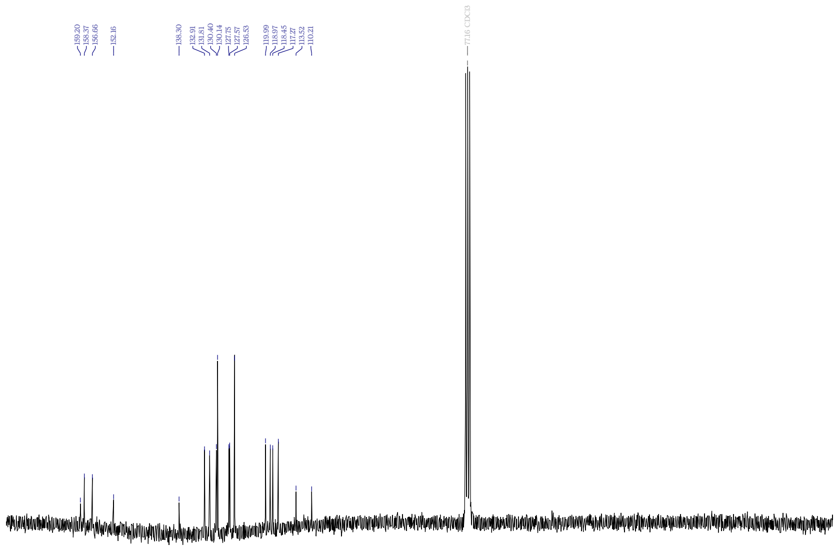

^{13}C-NMR spectrum of **1** (75 MHz, $CDCl_3$)

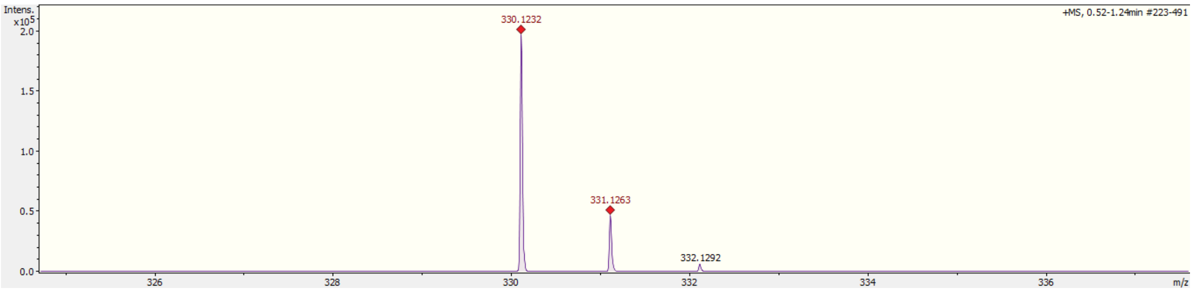

HRMS (ESI) analysis of **1**

3.3. **Galactose intermediate 1**

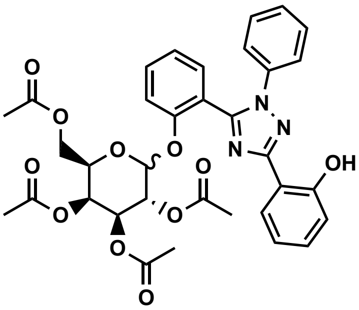

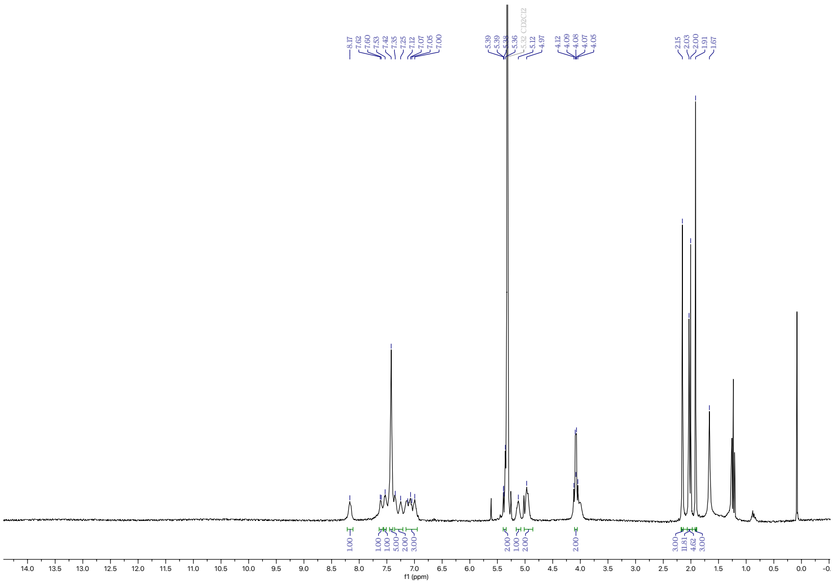

^{1}H-NMR spectrum of **Galactose intermediate 1** (300 MHz, CD_2Cl_2

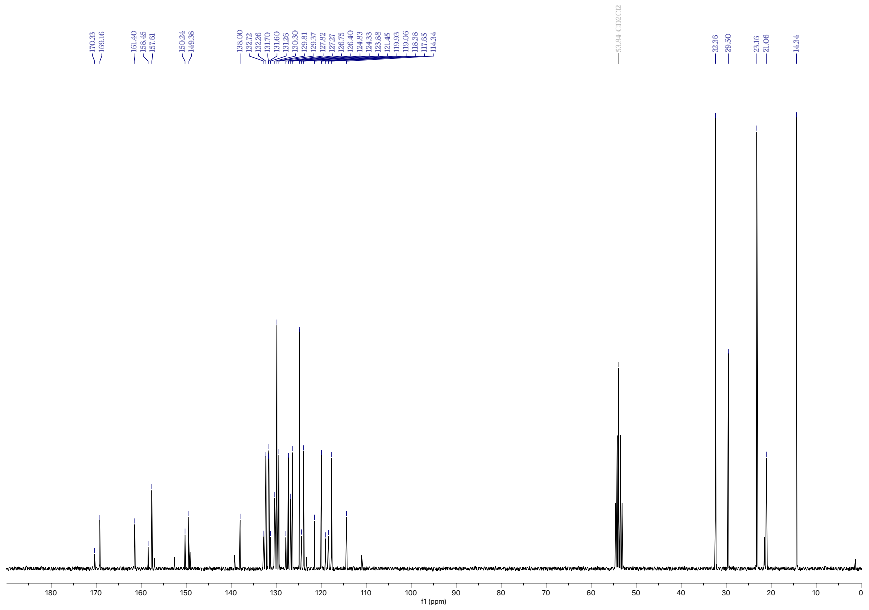

^{13}C-NMR spectrum of **Galactose intermediate 1** (75 MHz, CD_2Cl_2)

3.4. **2a**

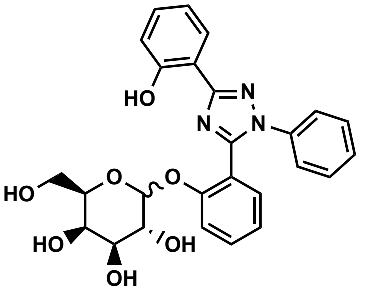

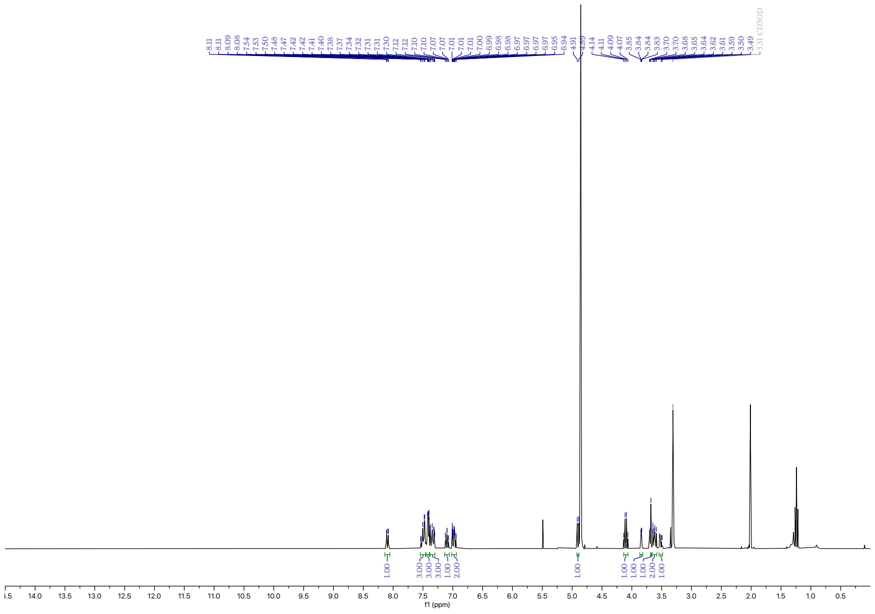

^{1}H-NMR spectrum of **2a** (300 MHz, MeOD-d_4)

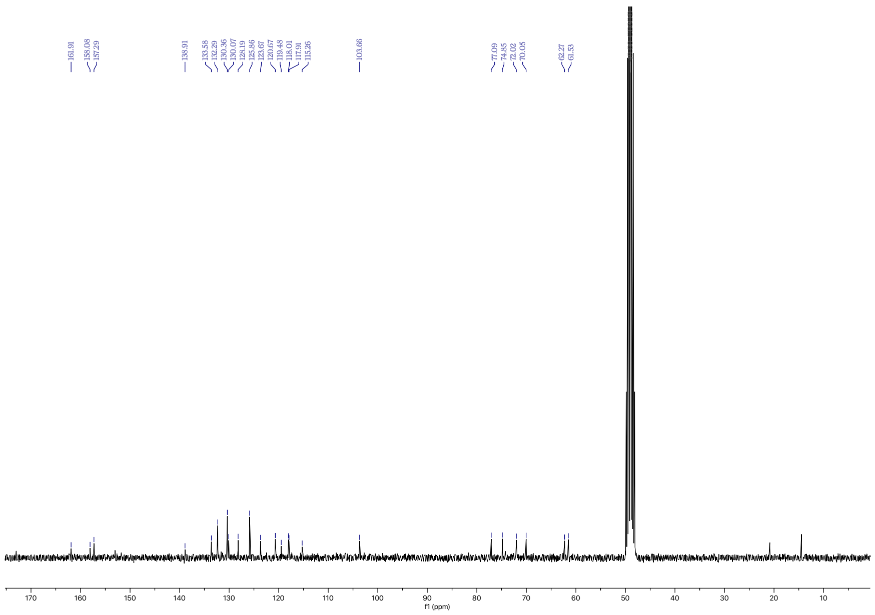

^{13}C-NMR spectrum of **2a** (75 MHz, MeOD-d$_4$)

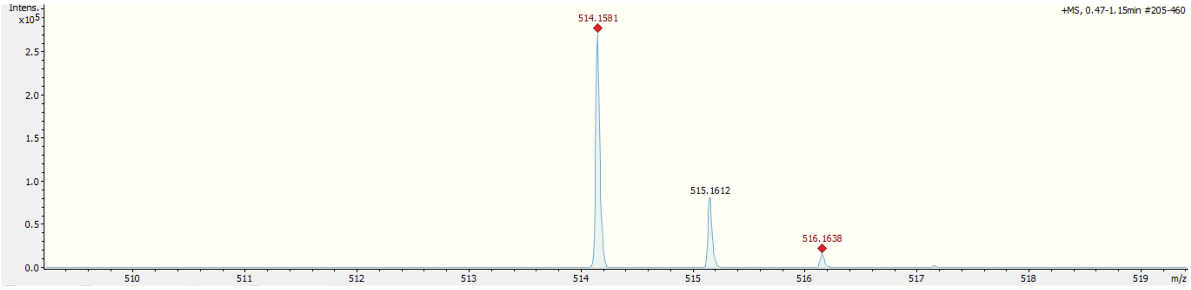

HRMS (ESI) analysis of **2a**

3.5. **Glucose intermediate 1**

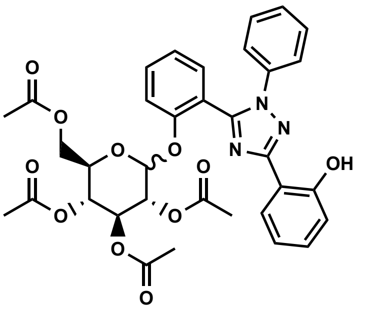

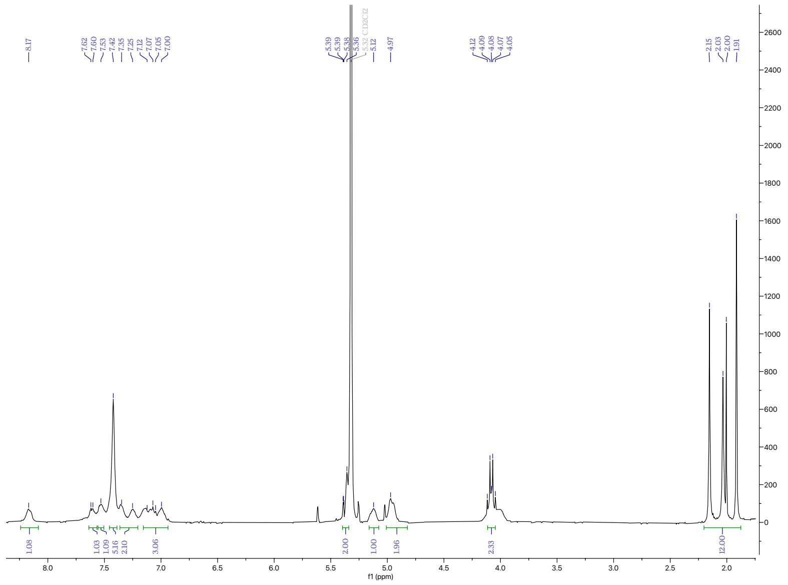

^{1}H-NMR spectrum of **Glucose intermediate 1** (300 MHz, CD_2Cl_2)

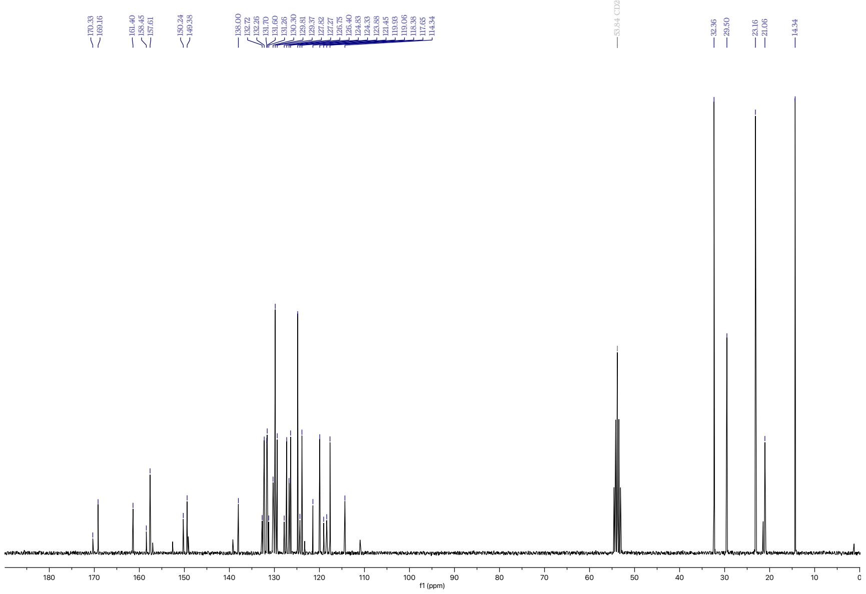

^{13}C-NMR spectrum of **Glucose intermediate 1** (75 MHz, CD_2Cl_2)

3.6. **2b**

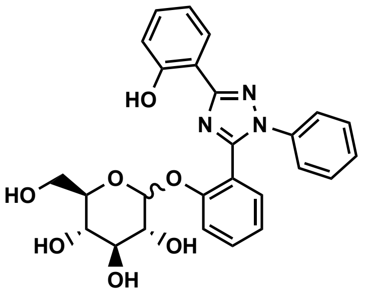

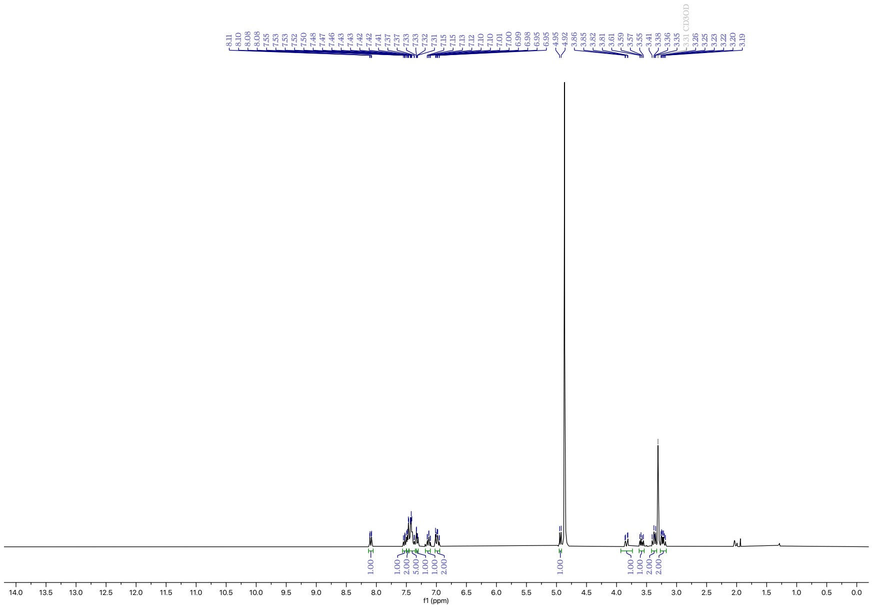

^{1}H-NMR spectrum of **2b** (300 MHz, MeOD-d$_4$)

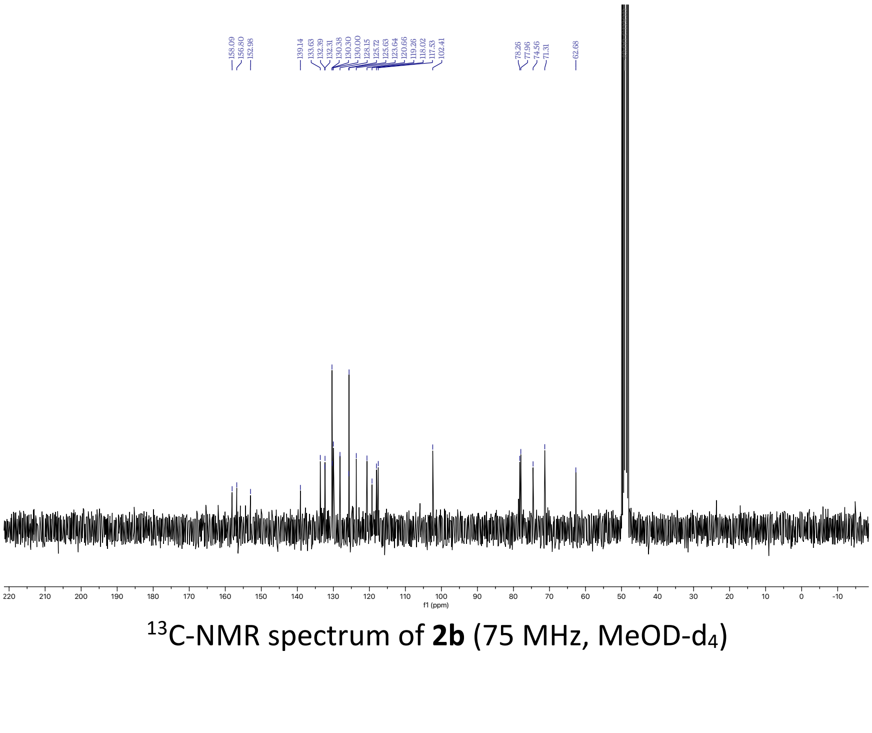

^{13}C-NMR spectrum of **2b** (75 MHz, MeOD-d$_4$)

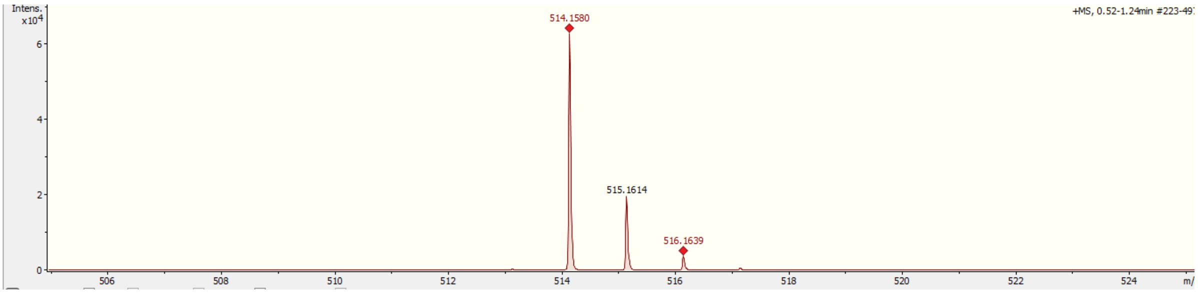

HRMS (ESI) analysis of **2b**

3.7. **Galactose linker intermediate 1**

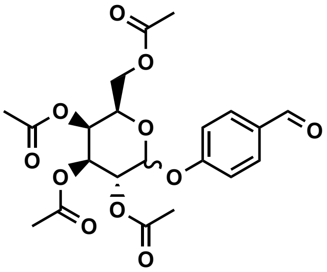

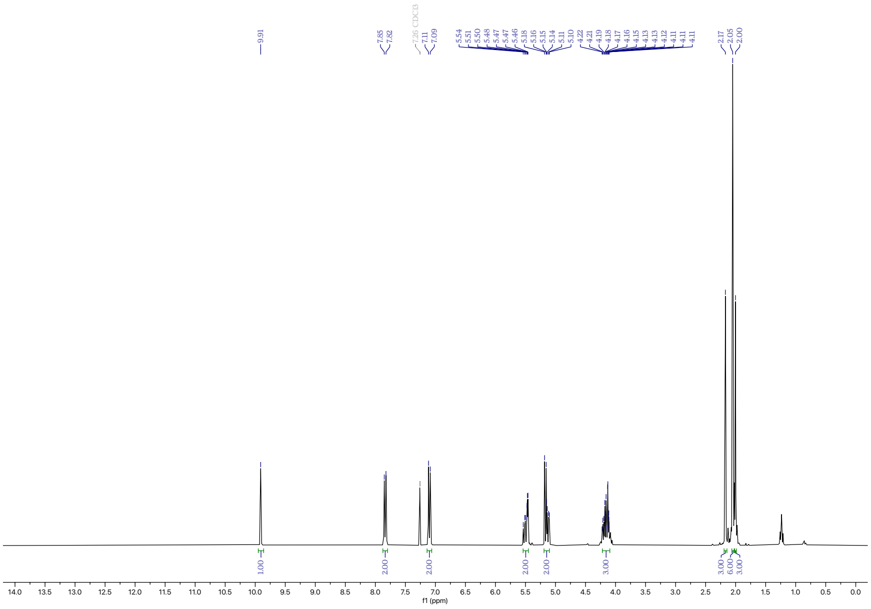

^{1}H-NMR spectrum of **Galactose linker intermediate 1** (300 MHz, $CDCl_3$)

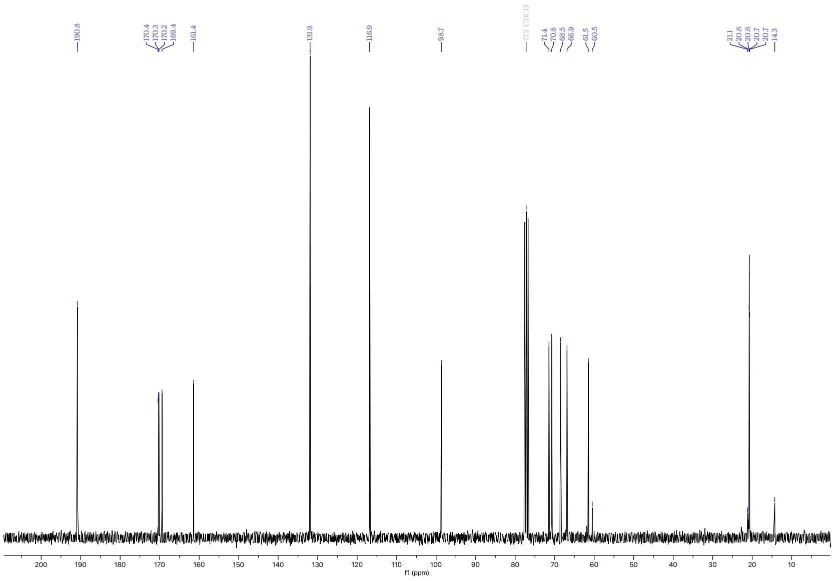

^{13}C-NMR spectrum of **Galactose linker intermediate 1** (75 MHz, $CDCl_3$)

3.8. **Galactose linker intermediate 2**

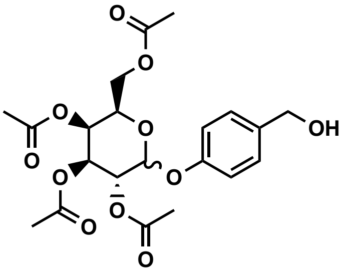

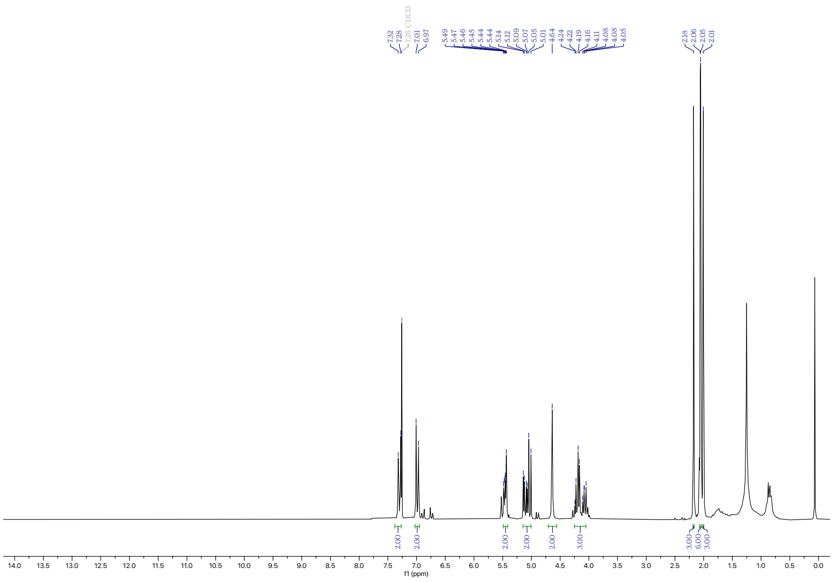

^{1}H-NMR spectrum of **Galactose linker intermediate 2** (300 MHz, $CDCl_3$)

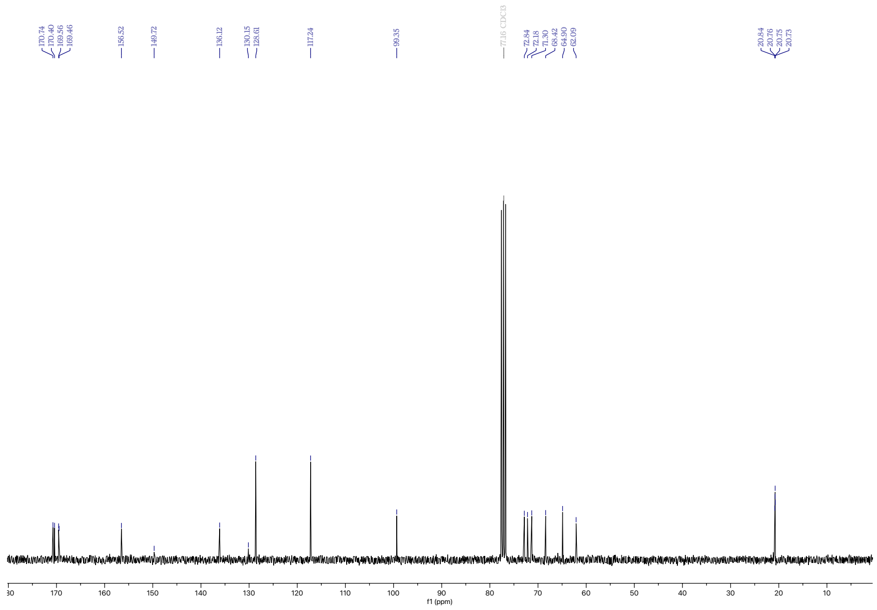

^{13}C-NMR spectrum of **Galactose linker intermediate 2** (75 MHz, $CDCl_3$)

3.9. **Galactose linker intermediate 3**

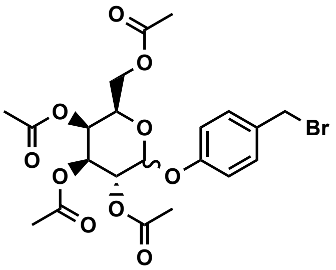

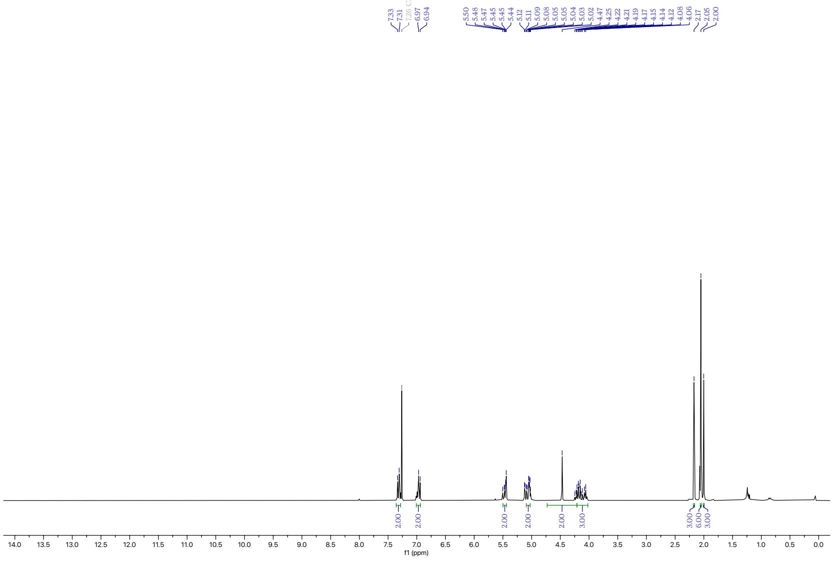

^{1}H-NMR spectrum of **Galactose linker intermediate 3** (300 MHz, $CDCl_3$)

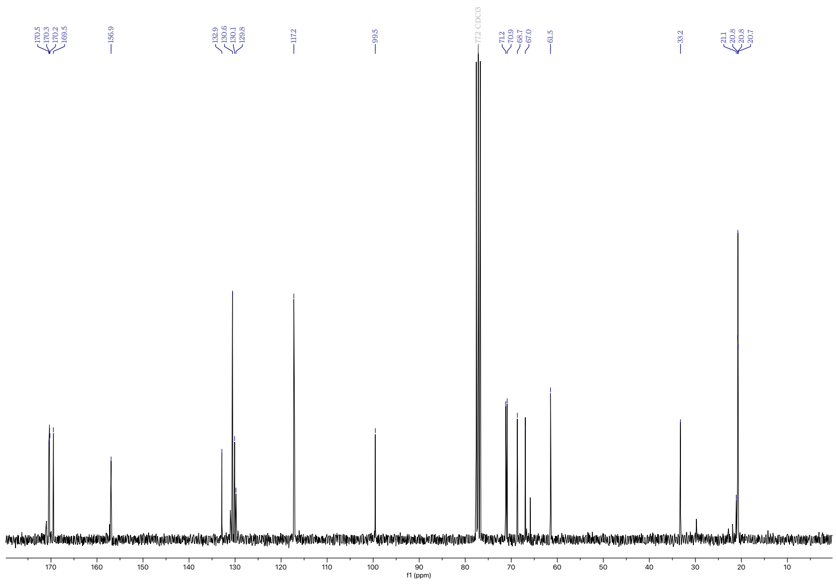

^{13}C-NMR spectrum of **Galactose linker intermediate 3** (75 MHz, $CDCl_3$)

3.10. Galactose linker intermediate 4

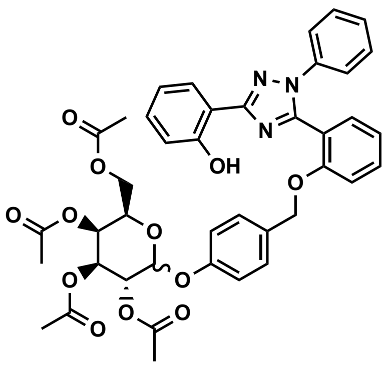

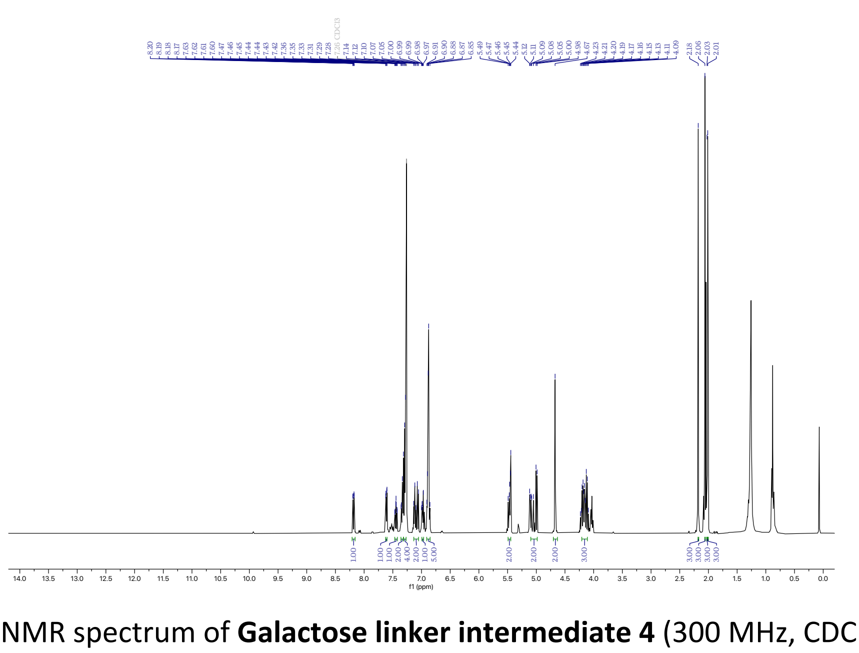

^{1}H-NMR spectrum of **Galactose linker intermediate 4** (300 MHz, $CDCl_3$)

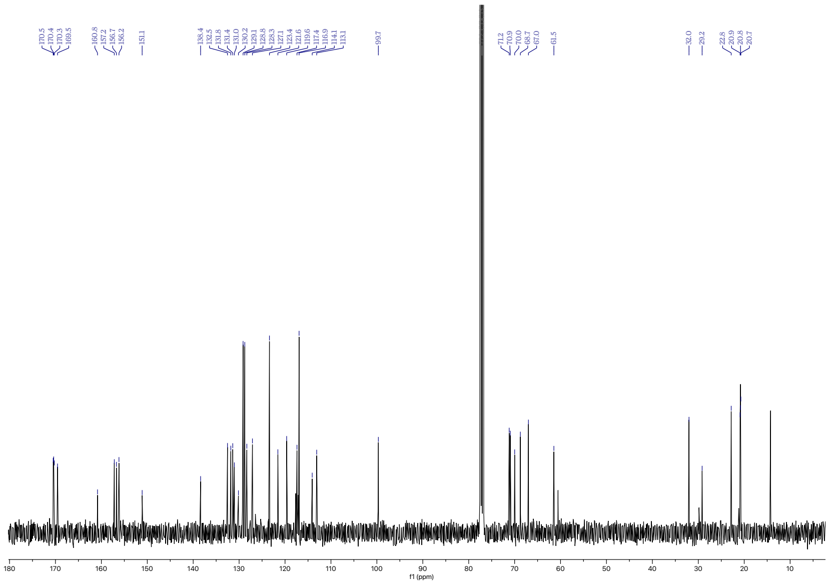

^{13}C-NMR spectrum of **Galactose linker intermediate 4** (75 MHz, $CDCl_3$)

3.11. **3a**

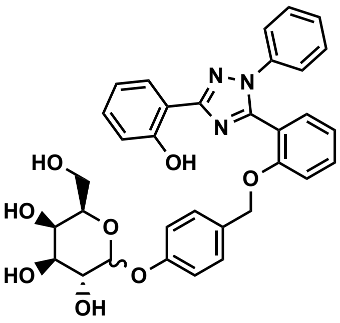

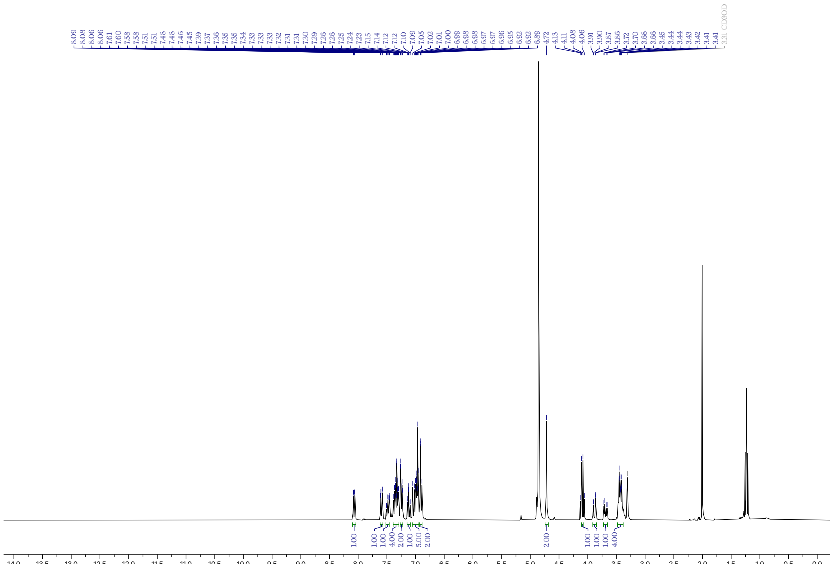

^{1}H-NMR spectrum of **3a** (300 MHz, MeOD-d_4)

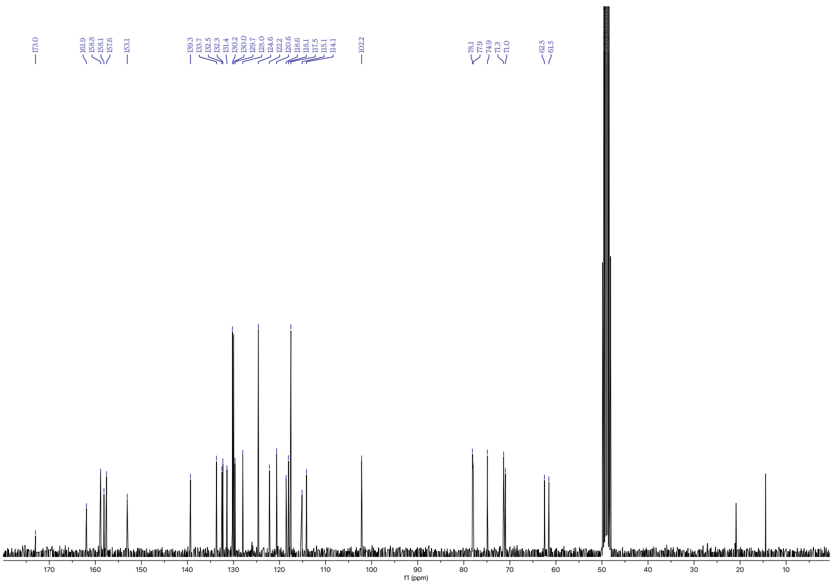

^{13}C-NMR spectrum of **3a** (75 MHz, MeOD-d$_4$)

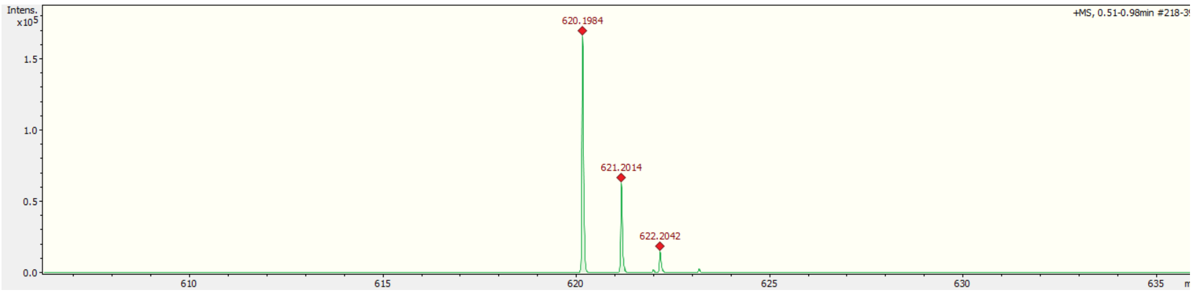

HRMS (ESI) analysis of **3a**

3.12. **Glucose linker intermediate 1**

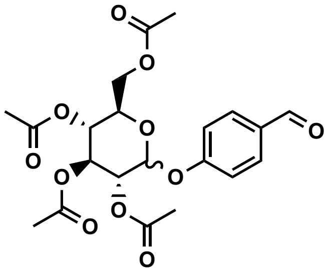

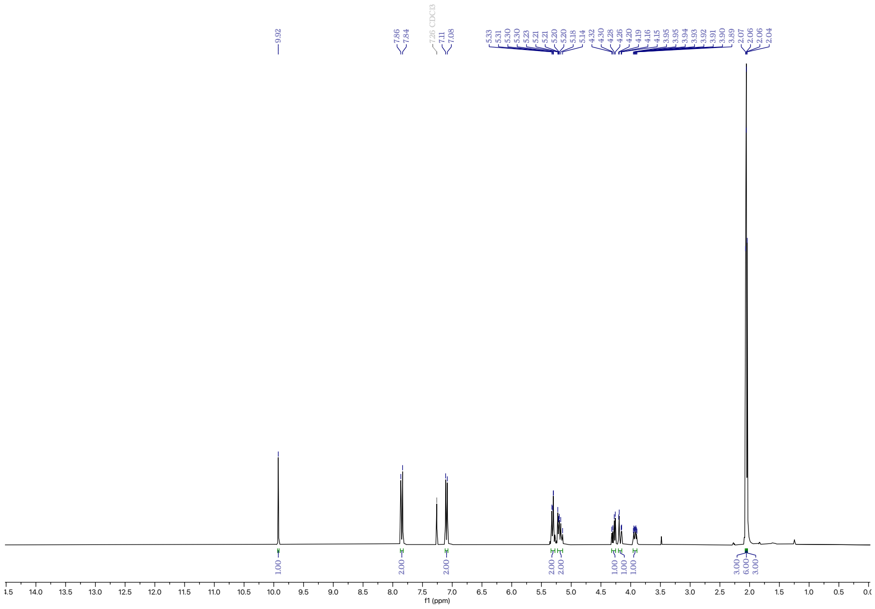

^{1}H-NMR spectrum of **Glucose linker intermediate 1** (300 MHz, $CDCl_3$)

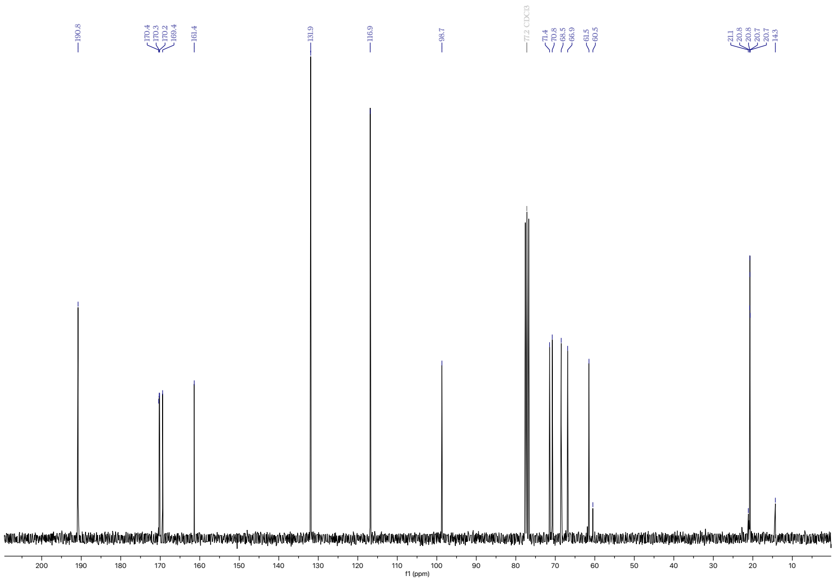

^{13}C-NMR spectrum of **Glucose linker intermediate 1** (75 MHz, $CDCl_3$)

3.13. **Glucose linker intermediate 2**

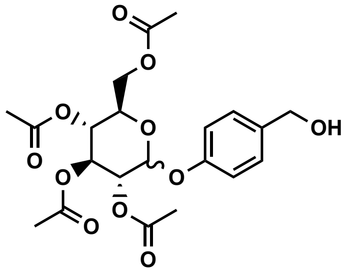

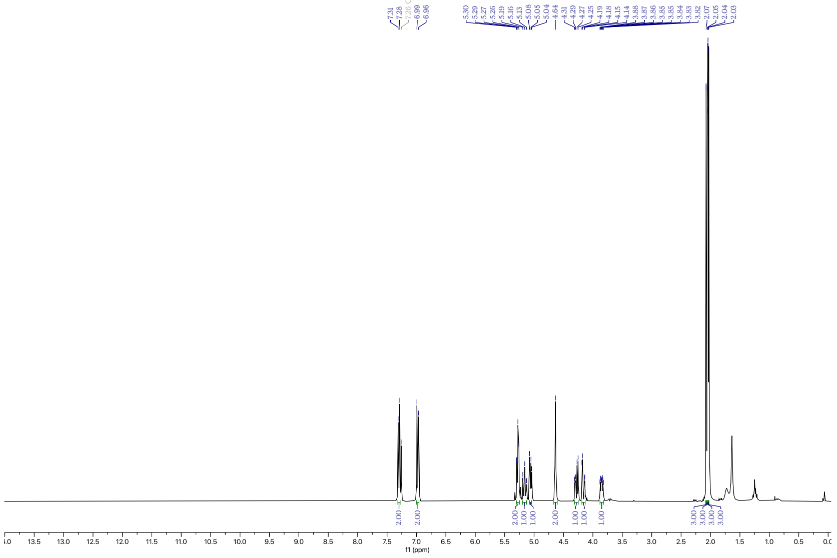

^{1}H-NMR spectrum of **Glucose linker intermediate 2** (300 MHz, $CDCl_3$)

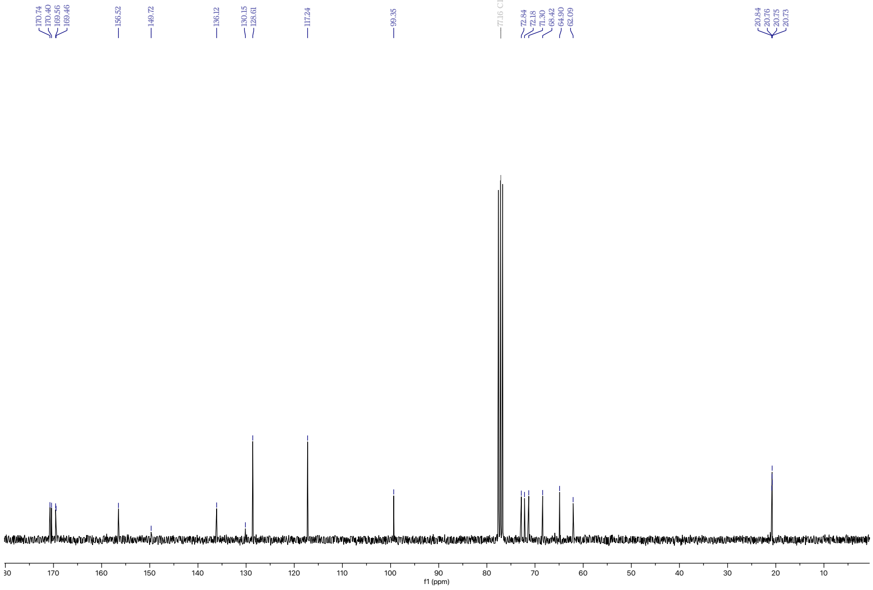

^{13}C-NMR spectrum of **Glucose linker intermediate 2** (75 MHz, $CDCl_3$)

3.14. **Glucose linker intermediate 3**

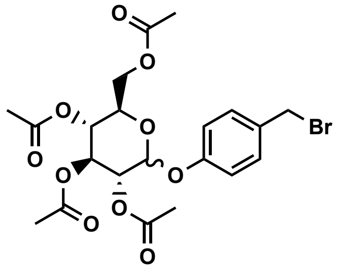

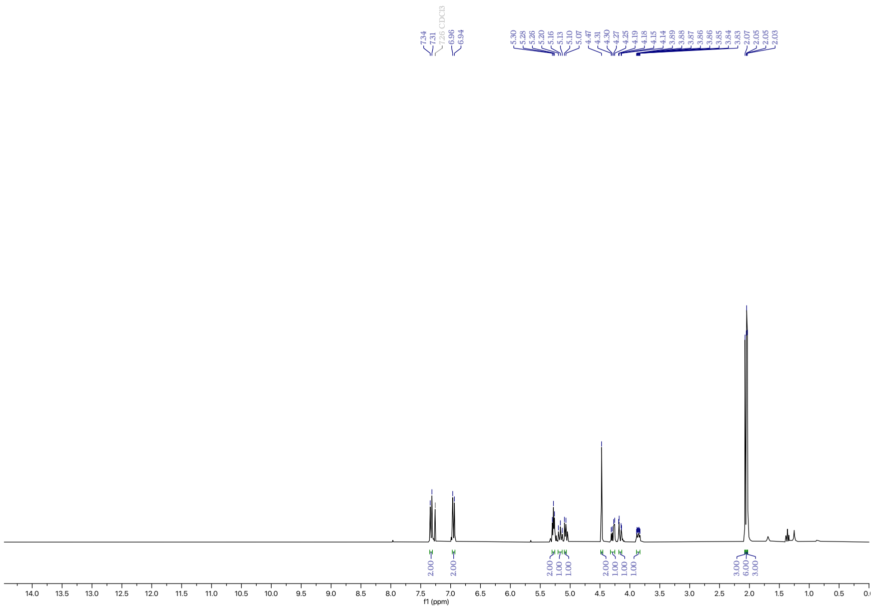

^{1}H-NMR spectrum of **Glucose linker intermediate 3** (300 MHz, $CDCl_3$)

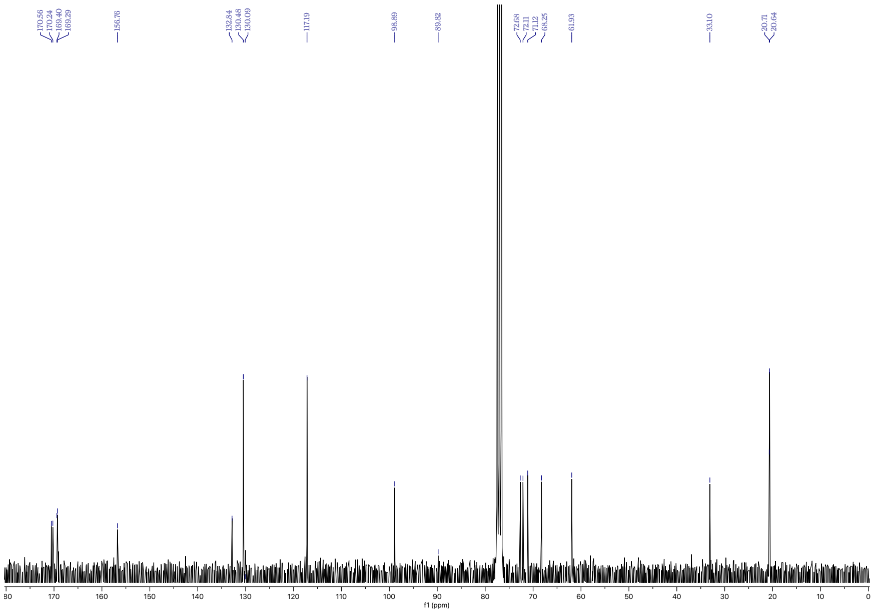

^{13}C-NMR spectrum of **Glucose linker intermediate 3** (75 MHz, $CDCl_3$)

3.15. **Glucose linker intermediate 4**

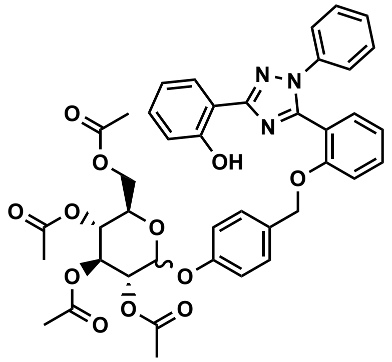

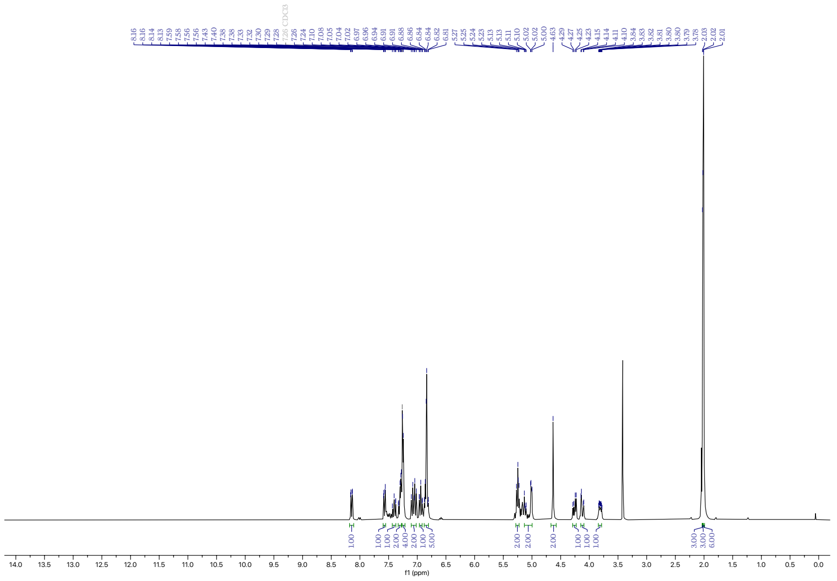

^{1}H-NMR spectrum of **Glucose linker intermediate 4** (300 MHz, $CDCl_3$)

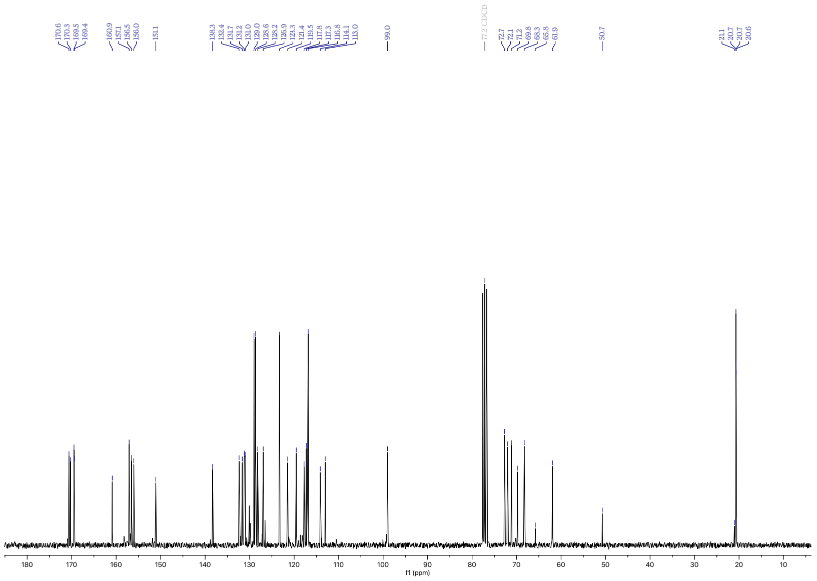

^{13}C-NMR spectrum of **Glucose linker intermediate 4** (75 MHz, $CDCl_3$)

3.16. **3b**

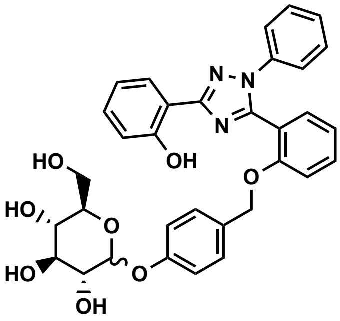

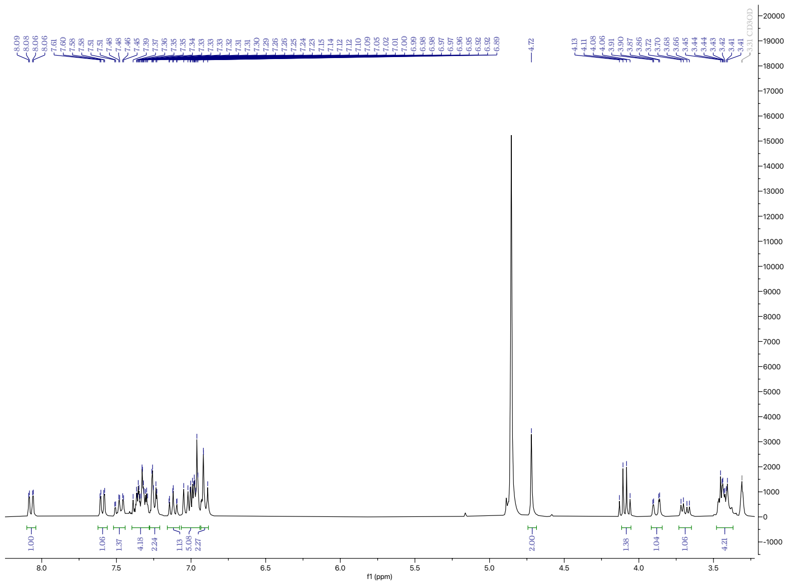

^{1}H-NMR spectrum of **3b** (300 MHz, MeOD-d$_4$)

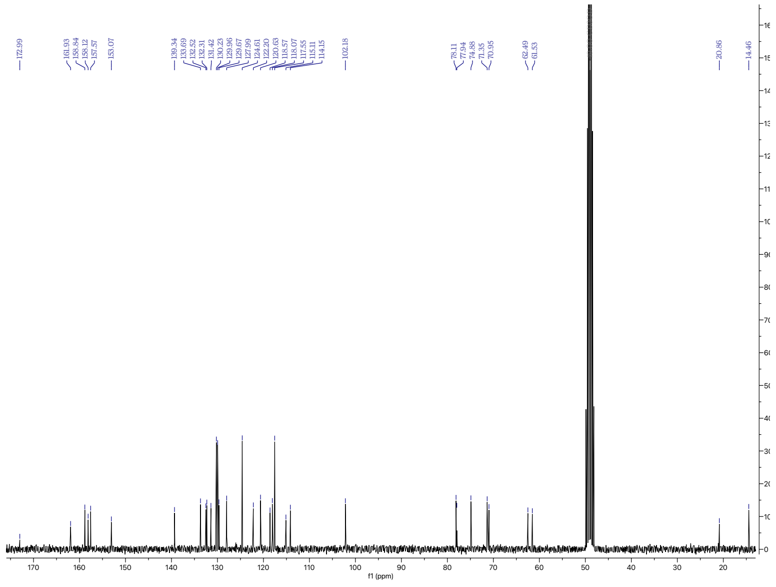

^{13}C-NMR spectrum of **3b** (75 MHz, MeOD-d$_4$)

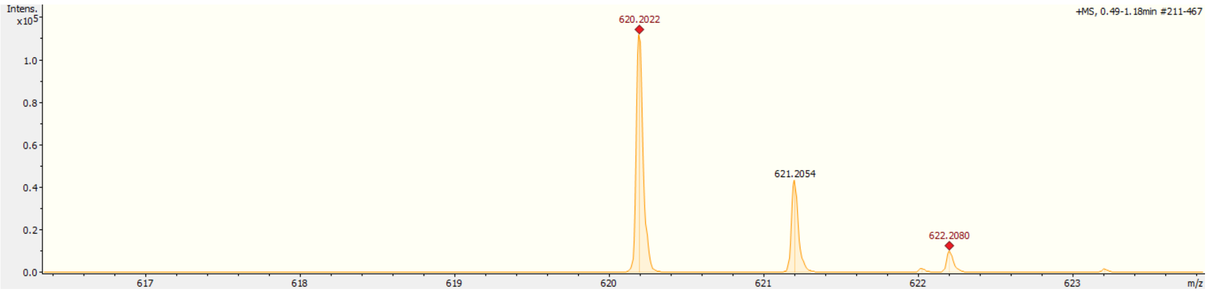

HRMS (ESI) analysis of **3b**

4. Cell-Viability Studies

Compound	A549	OvCar-3	GM-5756
**1**	3.1 ± 0.4 µM	3.9 ± 1.6 µM	2.7 ± 0.1 µM
**2a**	74.4 ± 14.1 µM	67.6 ± 13.0 µM	>100 µM
**2b**	>100 µM	76.5 ± 0.8 µM	>100 µM
**3a**	44.1 ± 2.4 µM	9.1 ± 1.6 µM	>100 µM
**3b**	7.4 ± 1.5 µM	3.7 ± 0.3 µM	15.3 ± 2.3 µM

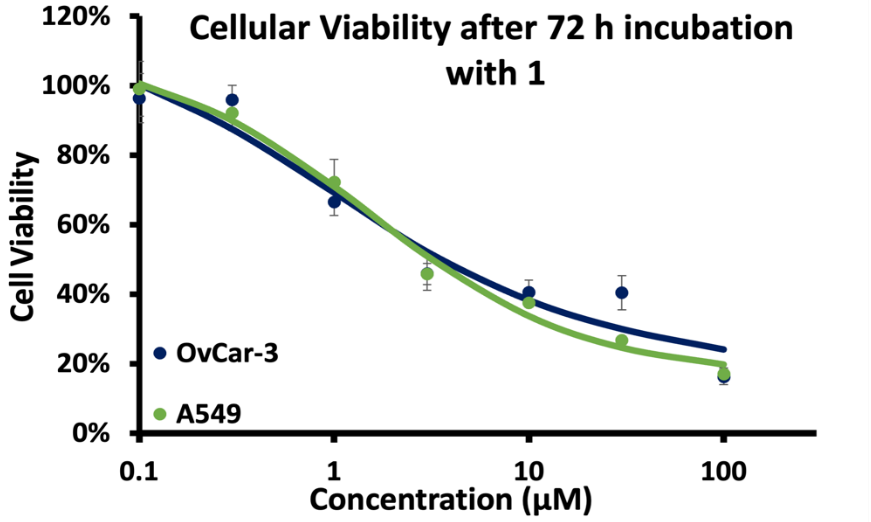

Figure S1: Proliferation profiles of OvCar-3 and A549 cells after 72 h incubation with **1** produced via MTT assay.

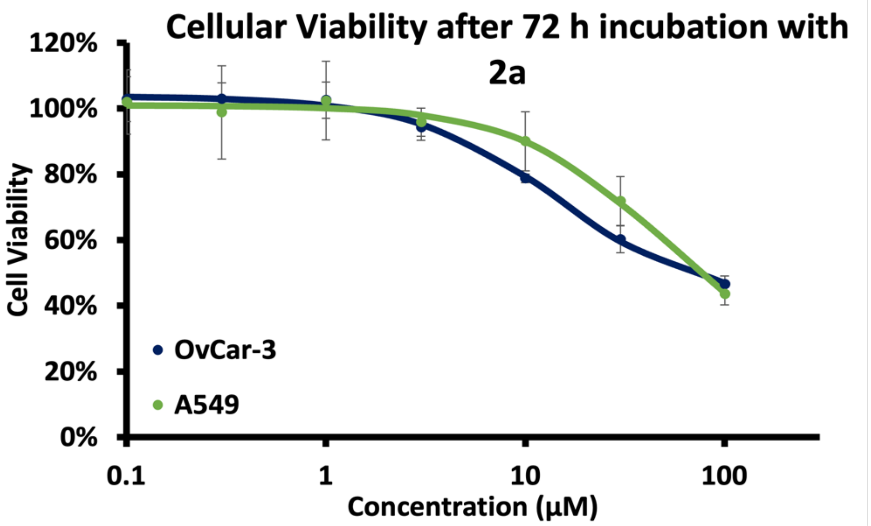

Figure S2: Proliferation profiles of OvCar-3 and A549 cells after 72 h incubation with **2a** produced via MTT assay.

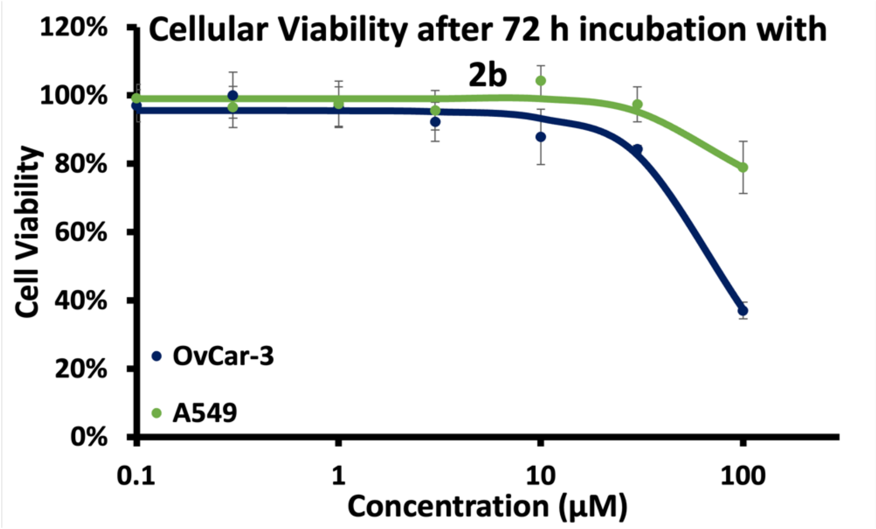

Figure S3: Proliferation profiles of OvCar-3 and A549 cells after 72 h incubation with **2b** produced via MTT assay.

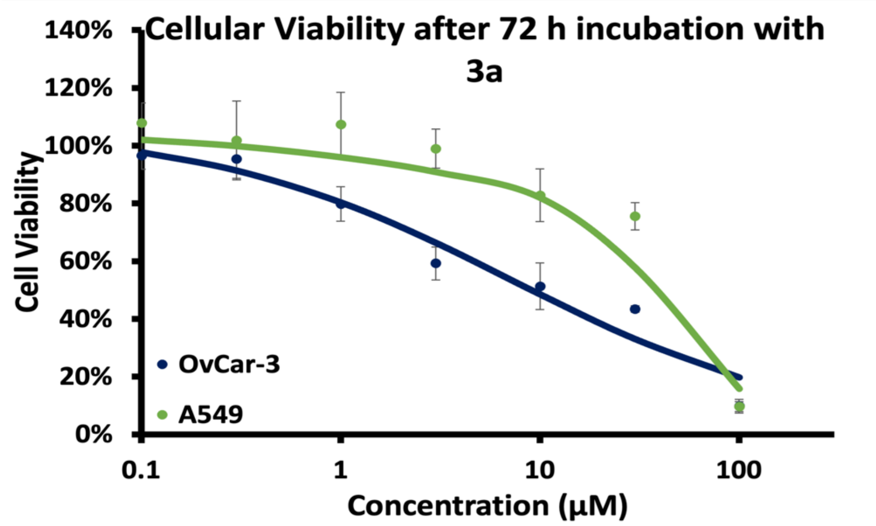

Figure S4: Proliferation profiles of OvCar-3 and A549 cells after 72 h incubation with **3a** produced via MTT assay.

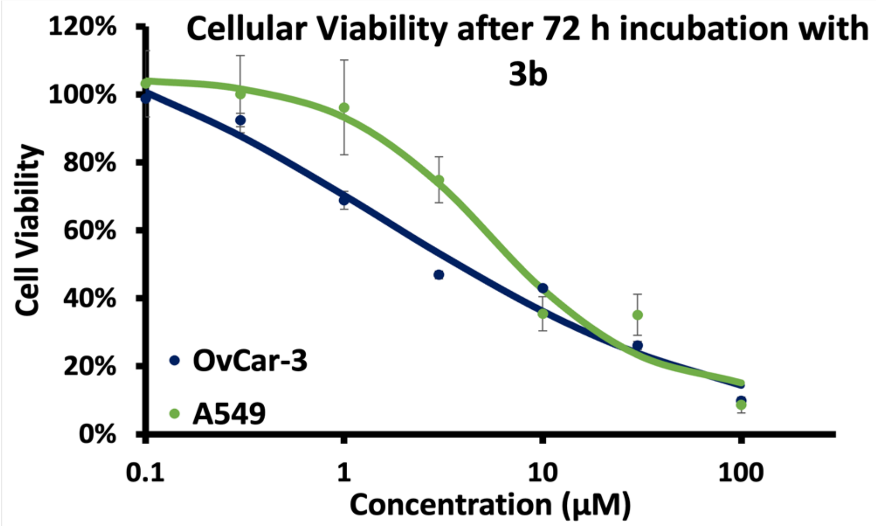

Figure S5: Proliferation profiles of OvCar-3 and A549 cells after 72 h incubation with **3b** produced via MTT assay.

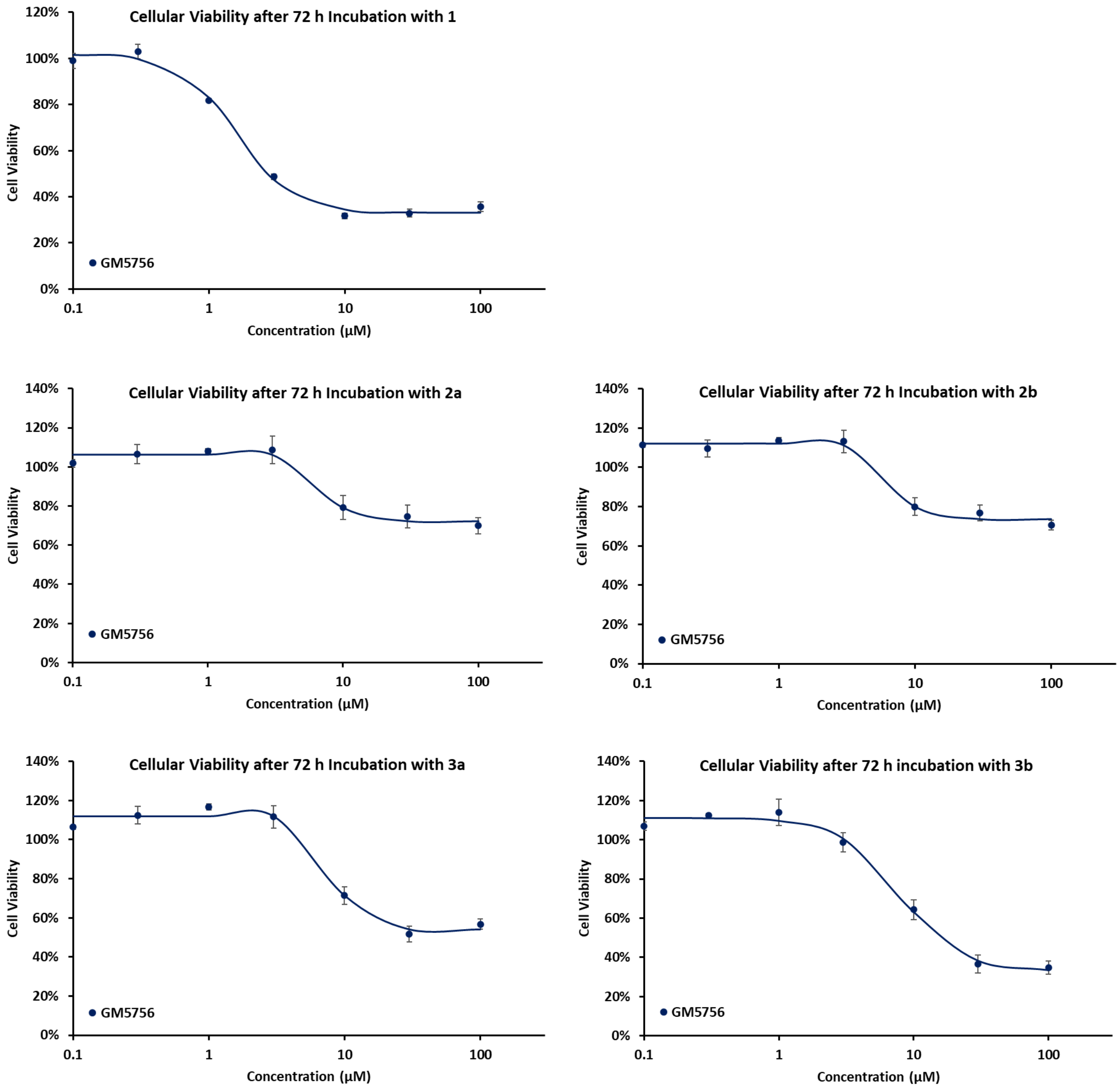

Figure S6: Proliferation profiles of GM5756 cells after 72 h incubation with the incubated compound produced via MTT assay.

5. UV-vis Spectroscopy

UV-vis spectra were recorded in methanol containing the indicated ligand or prodrug at a concentration of 30 µM and as appropriate Fe(III) at a concentration of 50 µM. A stock solution of $FeCl_3$ in DMSO was used as source of Fe(III) and ligands were added from a 5 mM stock solution in DMSO. All samples contained a final concentration of <0.2% DMSO.

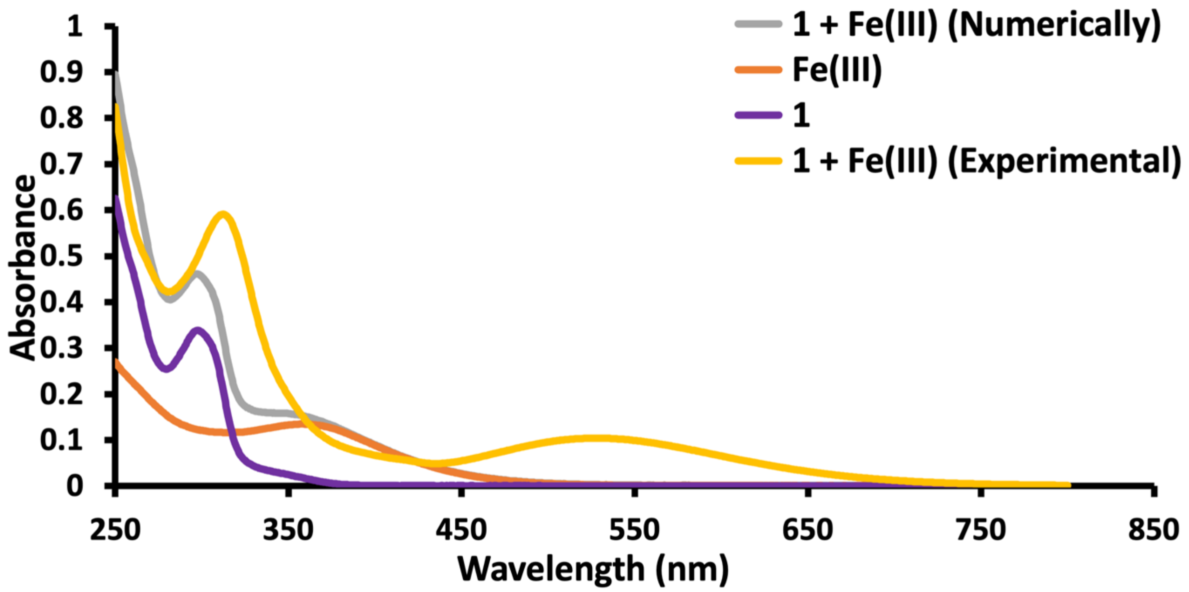

Figure S7: UV-vis absorption spectra of **1, Fe(III),** and **1 + Fe(III)**. The graph for **1 + Fe(III) (Numerically)** was constructed by numerically adding the respective signals of **Fe(III)** and **1** to show what graph could be expected if the two species were in the same solution without interacting.

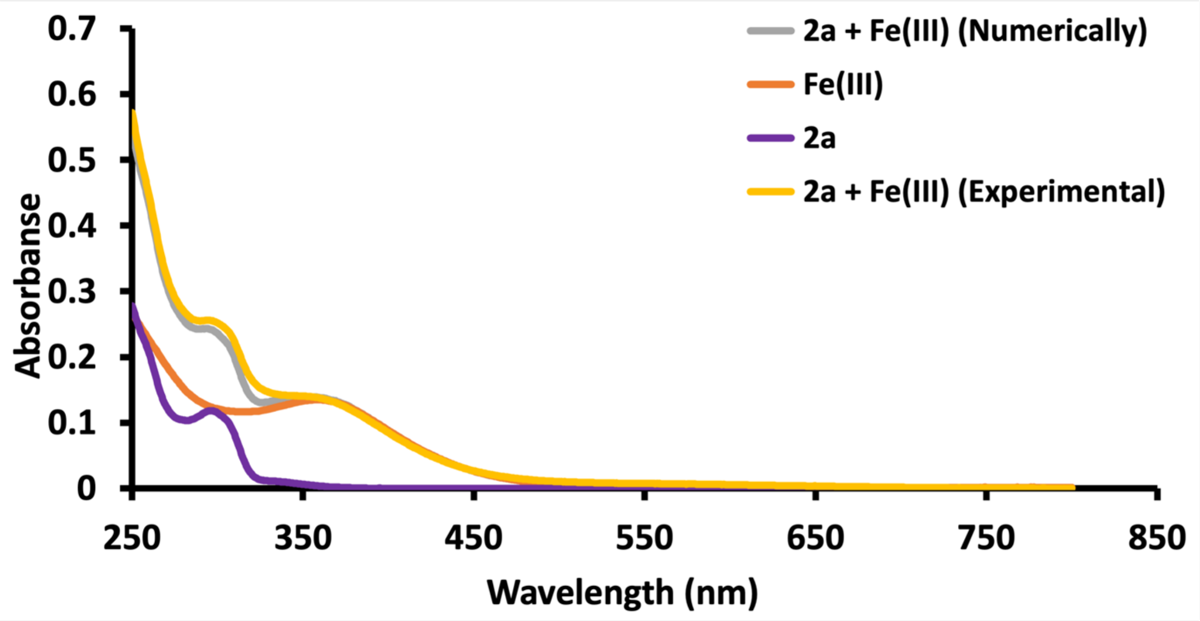

Figure S8: UV-vis absorption spectra of **2a, Fe(III),** and **2a + Fe(III)**. The graph for **2a + Fe(III) (Numerically)** was constructed by numerically adding the respective signals of **Fe(III)** and **2a** to show what graph could be expected if the two species were in the same solution without interacting.

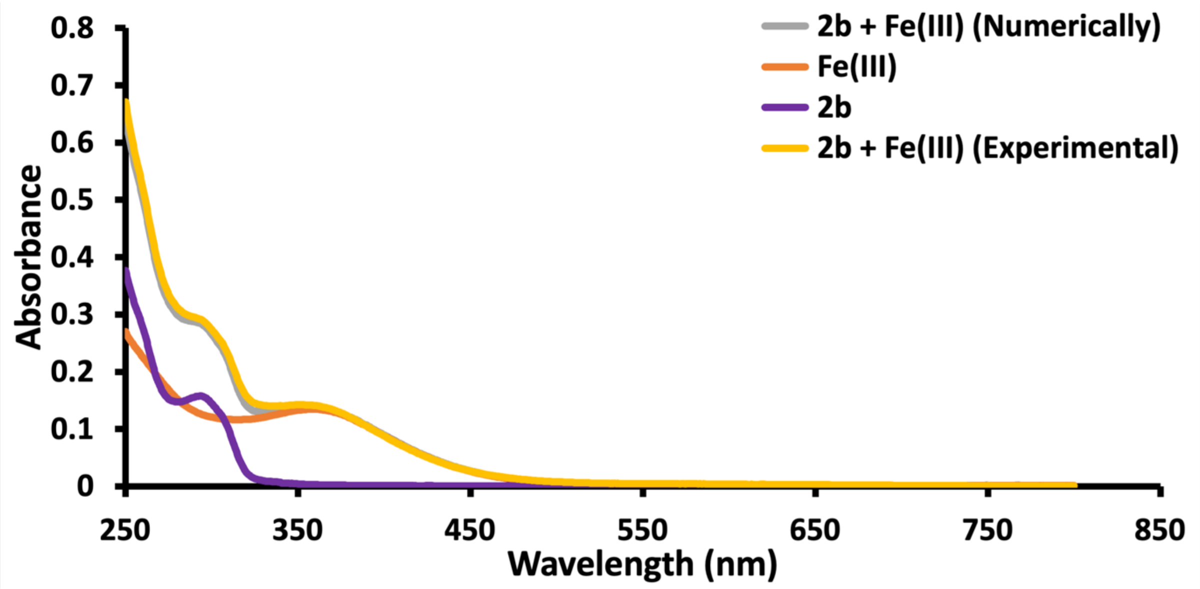

Figure S9: UV-vis absorption spectra of **2b, Fe(III),** and **2b + Fe(III)**. The graph for **2b + Fe(III) (Numerically)** was constructed by numerically adding the respective signals of **Fe(III)** and **2b** to show what graph could be expected if the two species were in the same solution without interacting.

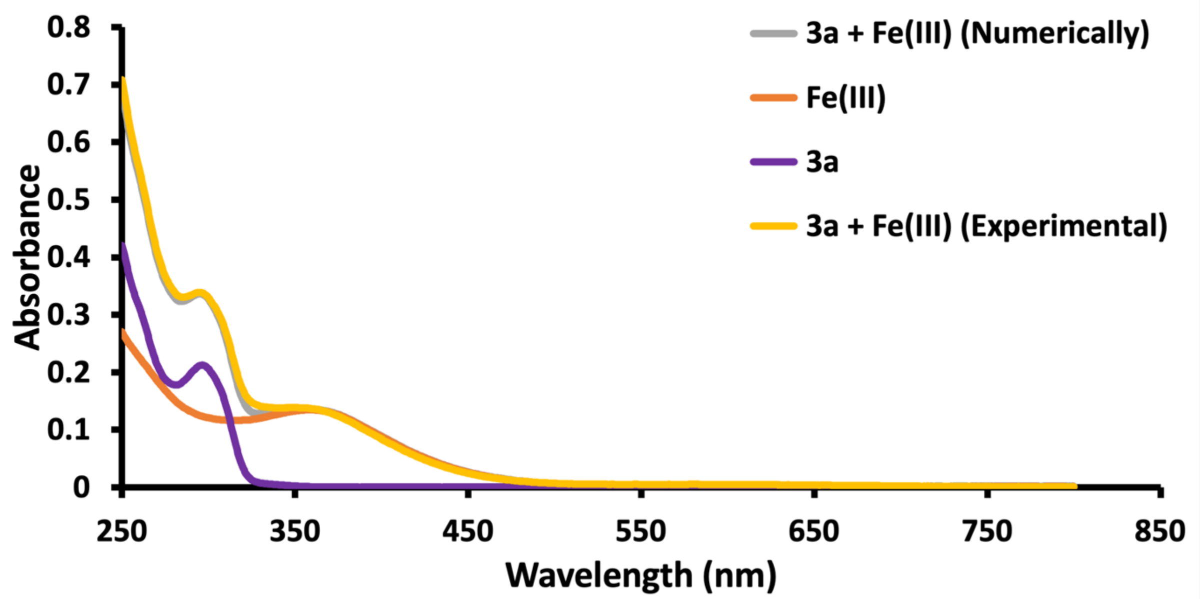

Figure S10: UV-vis absorption spectra of **3a, Fe(III),** and **3a + Fe(III)**. The graph for **3a + Fe(III) (Numerically)** was constructed by numerically adding the respective signals of **Fe(III)** and **3a** to show what graph could be expected if the two species were in the same solution without interacting

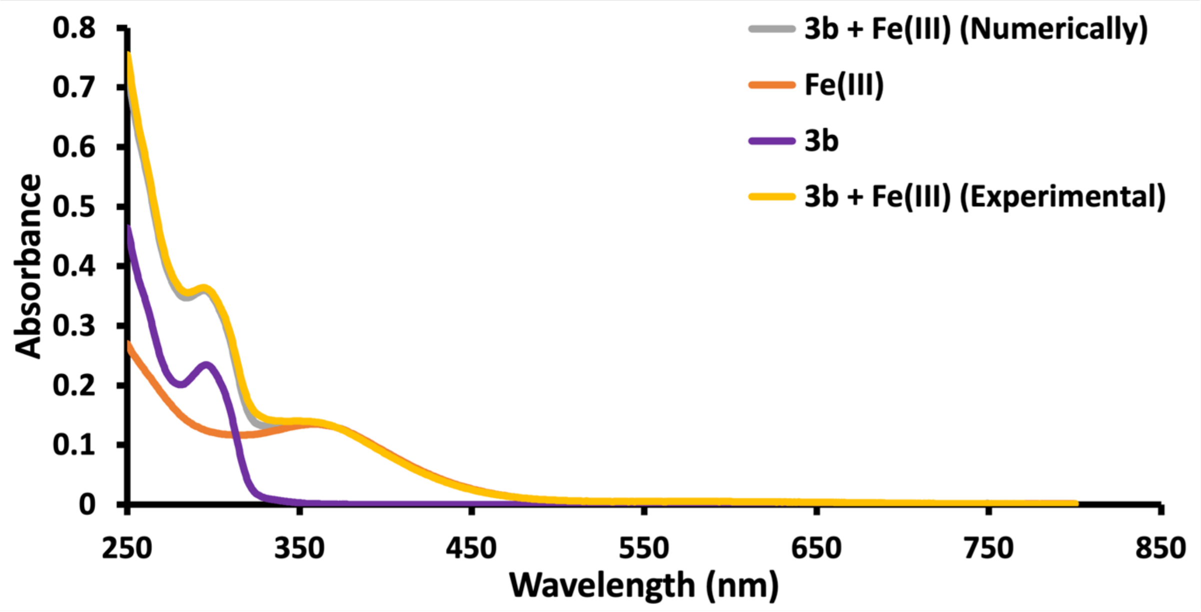

Figure S11: UV-vis absorption spectra of **3b, Fe(III),** and **3b + Fe(III).** The graph for **3b + Fe(III) (Numerically)** was constructed by numerically adding the respective signals of **Fe(III)** and **3b** to show what graph could be expected if the two species were in the same solution without interacting

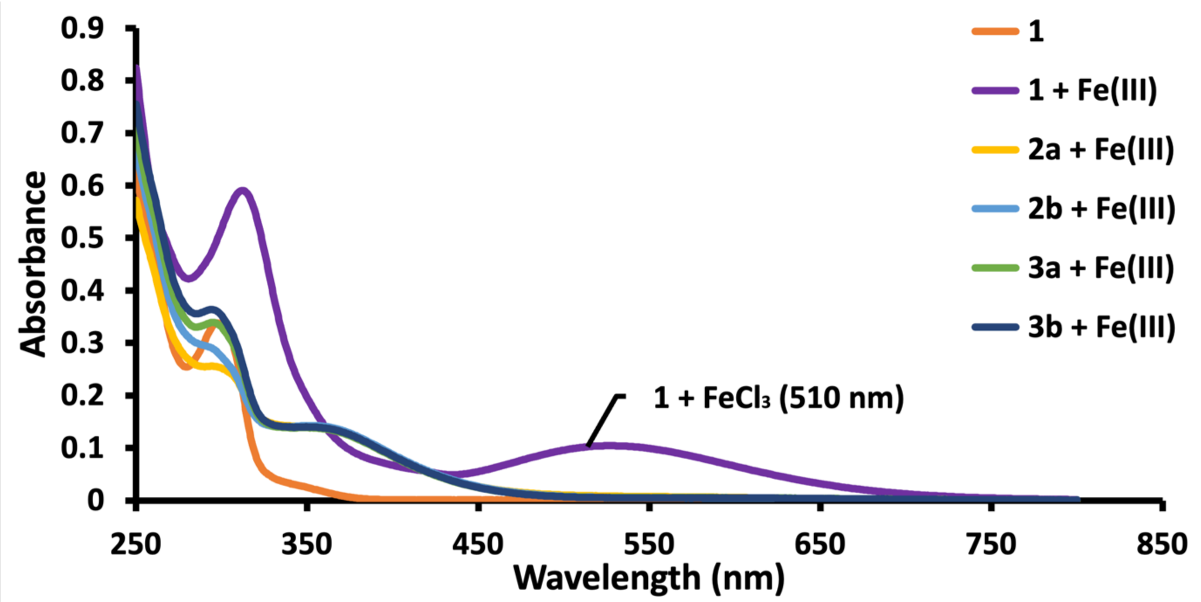

Figure S12: UV-vis absorption spectra of **1, 1 + Fe(III), 2a + Fe(III), 2b + Fe(III), 3a + Fe(III),** and **3b + Fe(III)**. Only the combination of the free chelator **1** and Fe(III) produced the characteristic absorption band around 510 nm that is indicative of complex formation between the ligand scaffold and Fe(III).

6. Enzyme Study (HPLC)

The three following enzymes were employed in this study:
(1) esterase from porcine liver sourced from Sigma Aldrich as dry powder with an activity of 229.4 units/mg protein as indicated by the supplier for the received lot;
(2) β-glucosidase from almonds sourced from Sigma Aldrich as dry powder with an activity of 11.7 U/mg as indicated by the supplier for the received lot
(3) β-galactosidase from *e.coli* overproducer sourced from Roche as prepared aqueous solution with an activity of 1500 units/mL protein as indicated by the supplier for the received lot.

As indicated for each experiment below, the respective enzyme was dissolved in PBS buffer and diluted as appropriate to produce the desired concentration.

25 mM stock solutions in DMSO were prepared for **2a**, **2b**, **3a**, and **3b**. From these stock solutions, 4 µL was added to 1 mL of PBS buffer, respectively, in separate glass vials, achieving a final concentration of 100 µM. The vials were then assigned to one of three experimental groups: control, negative, and positive.

In the control group, no enzyme was introduced. The negative group received 2 units of esterase enzyme, while the positive group received 2 units of the target enzyme as indicated, either β-galactosidase or β-glucosidase. All vials were incubated in a 37°C water bath for 48 hours. Aliquots were extracted from each vial after 24 and 48 hours, respectively, and analyzed as described below.

The extracted aliquots were analyzed via high-performance liquid chromatography (HPLC) with ultraviolet (UV) detection at a wavelength of 254 nm. Chromatograms were plotted with an offset of 1 along the y-axis to allow clear intercomparison of the data across the plotted data sets.

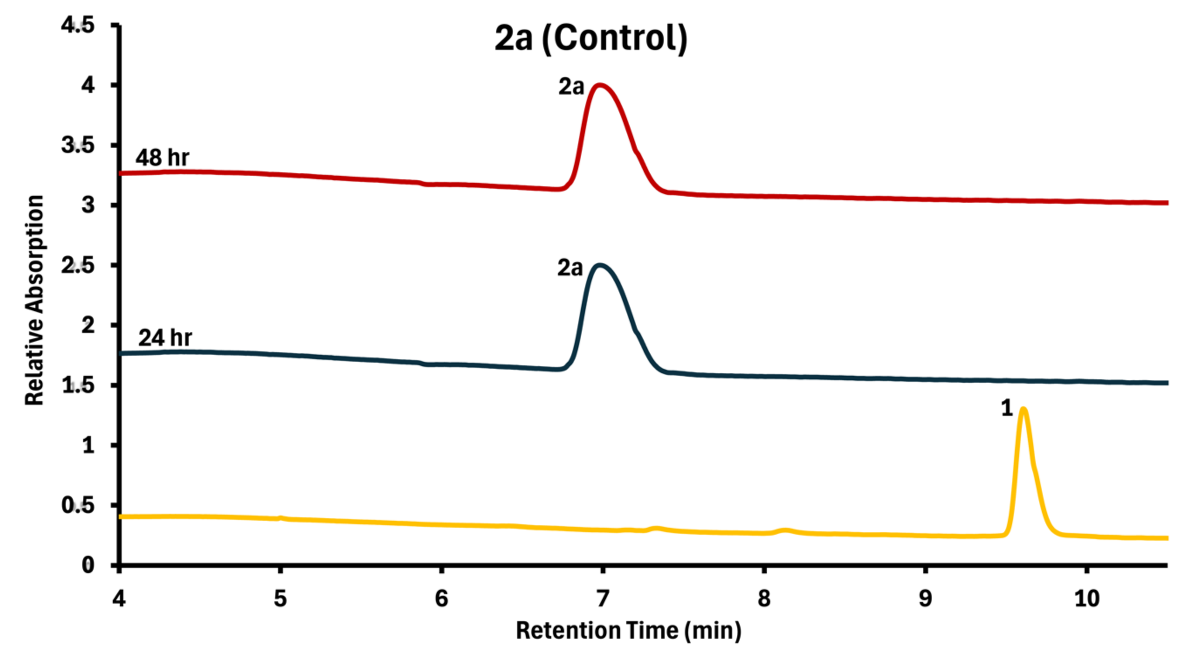

Figure S13: HPLC chromatograms for **2a** in the control group at 24 and 48 hours compared with **1**.

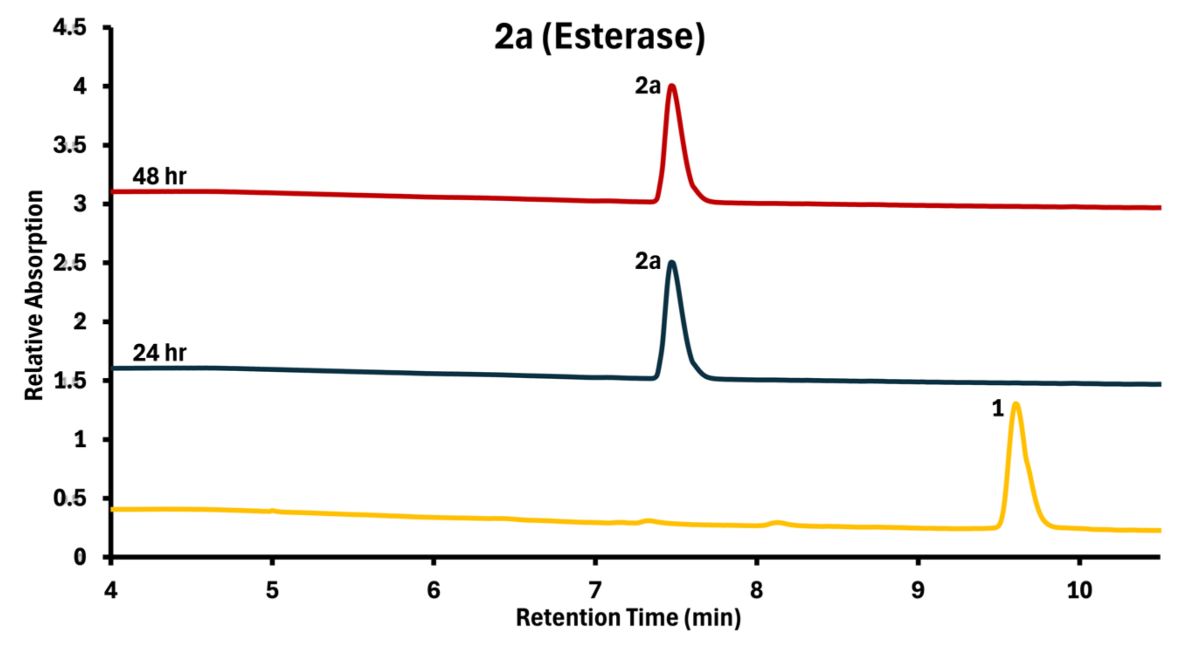

Figure S14: HPLC chromatograms for **2a** in the negative group at 24 and 48 hours compared with **1**.

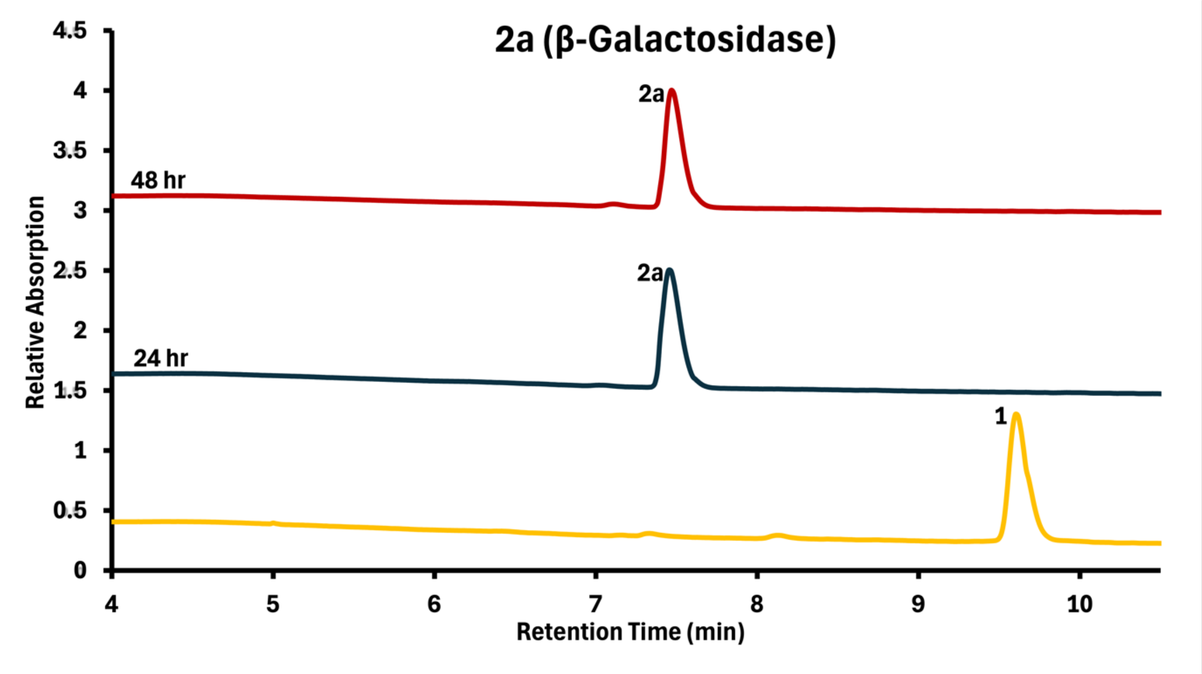

Figure S15: HPLC chromatograms for **2a** in the positive group at 24 and 48 hours compared with **1**.

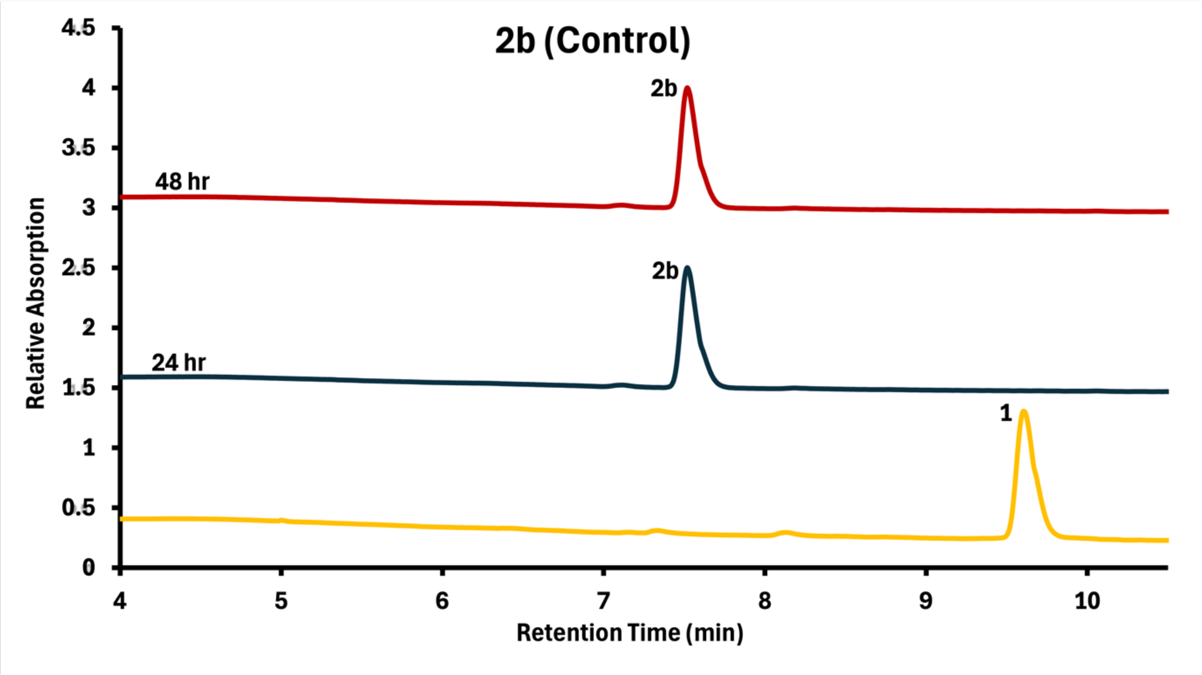

Figure S16: HPLC chromatograms for **2b** in the control group at 24 and 48 hours compared with **1.**

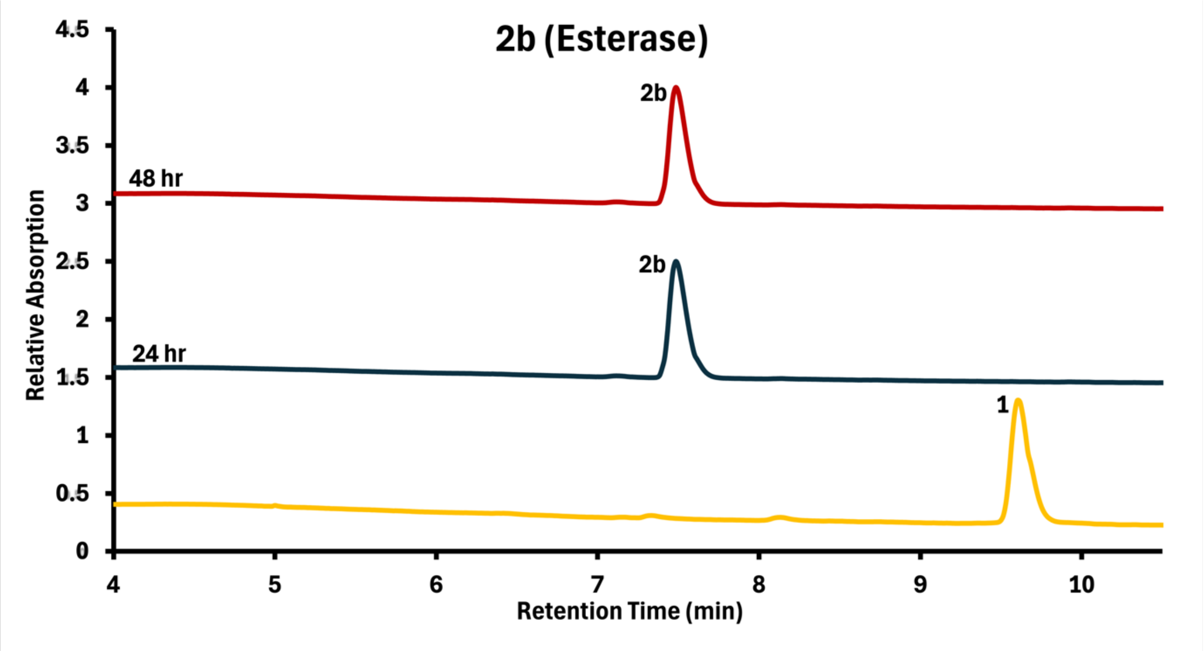

Figure S17: HPLC chromatograms for **2b** in the negative group at 24 and 48 hours compared with **1**.

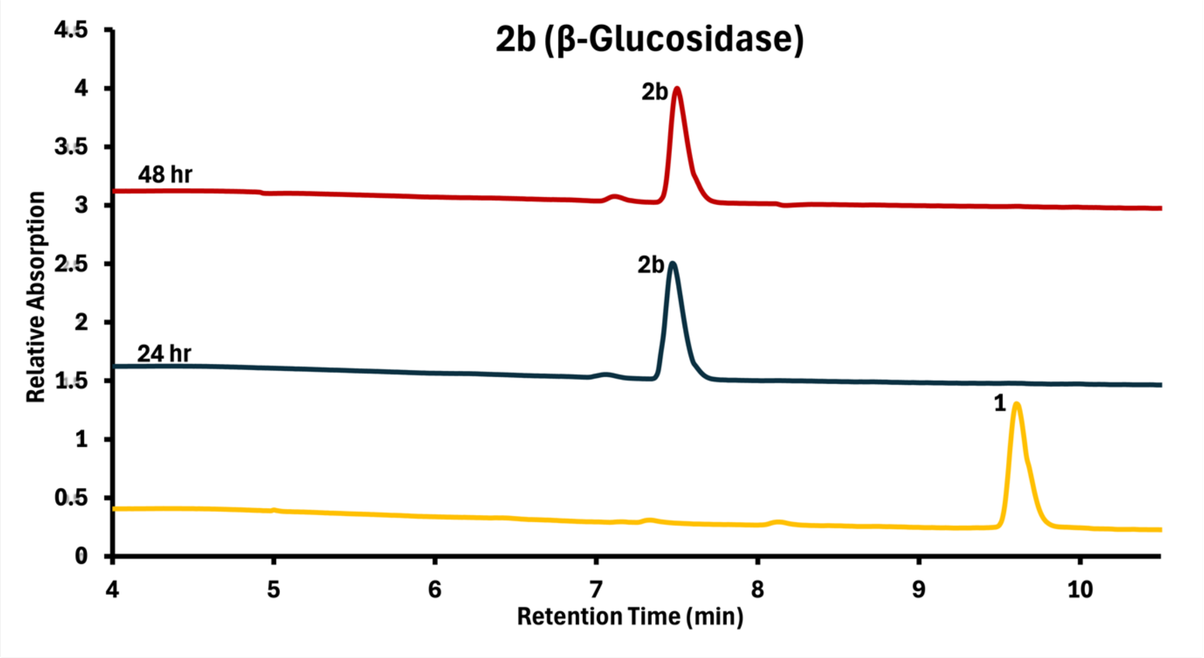

Figure S18: HPLC chromatograms for **2b** in the positive group at 24 and 48 hours compared with **1**.

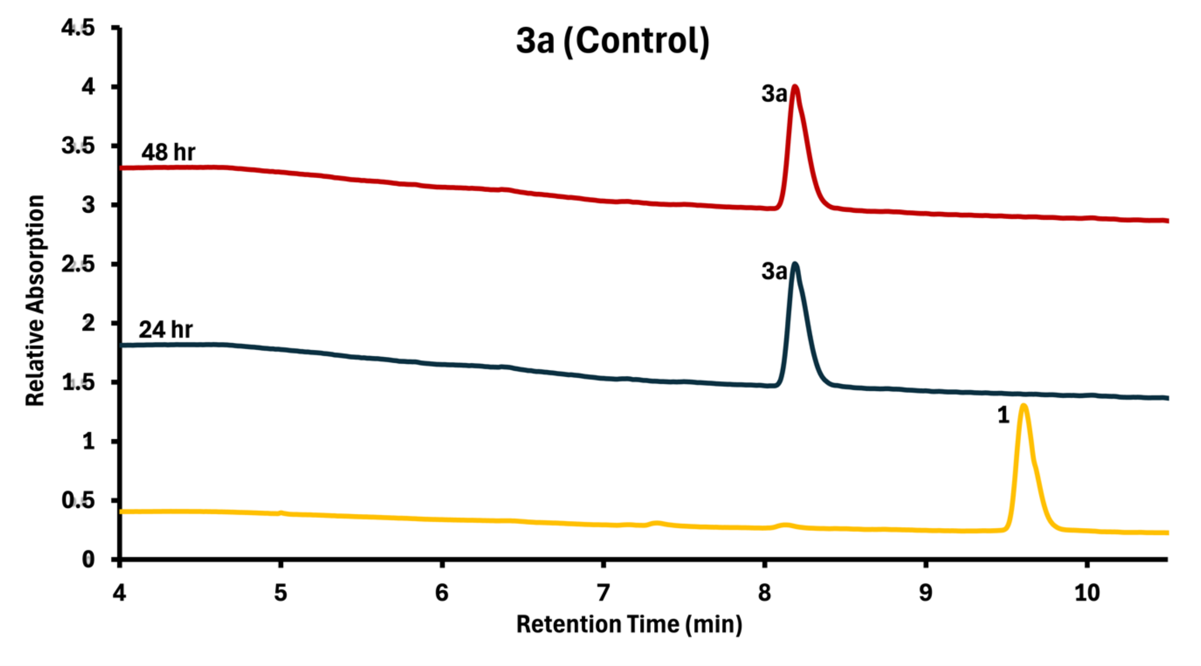

Figure S19: HPLC chromatograms for **3a** in the control group at 24 and 48 hours compared with **1**.

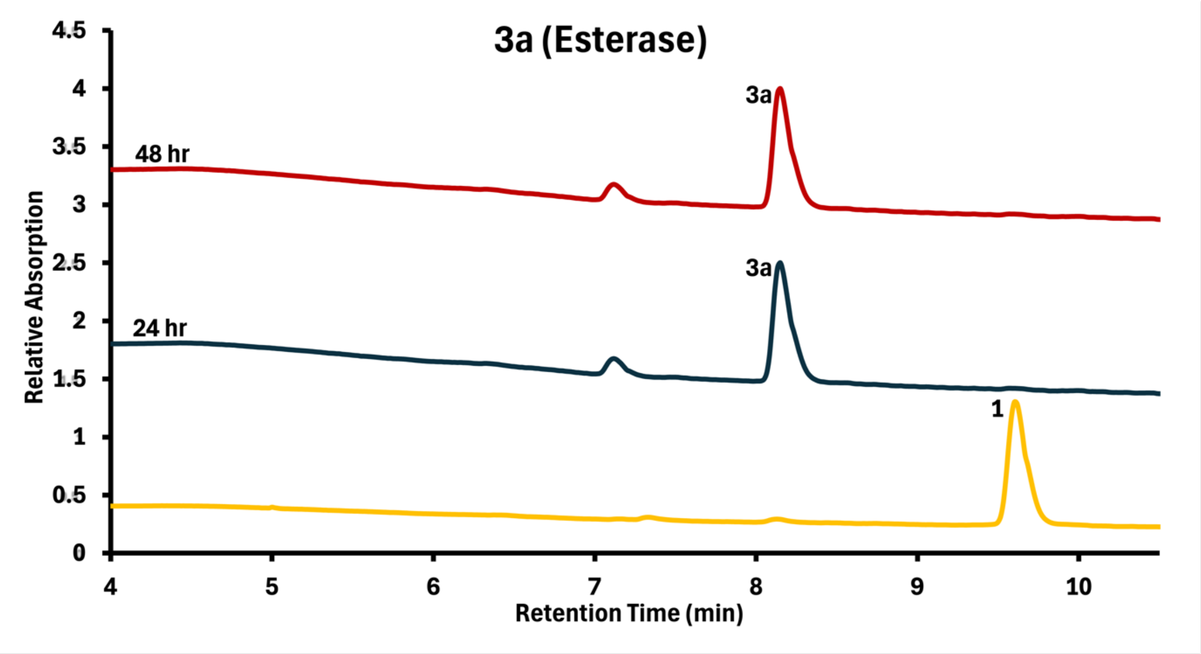

Figure S20: HPLC chromatograms for **3a** in the negative group at 24 and 48 hours compared with **1**.

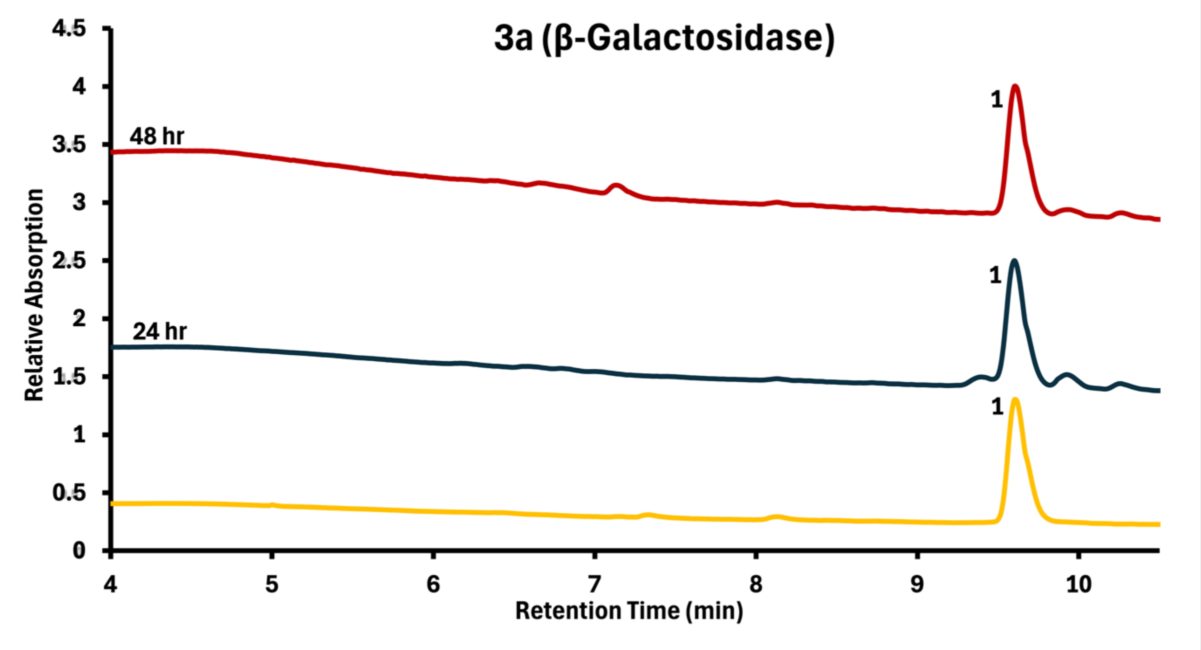

Figure S21: HPLC chromatograms for **3a** in the positive group at 24 and 48 hours compared with **1**.

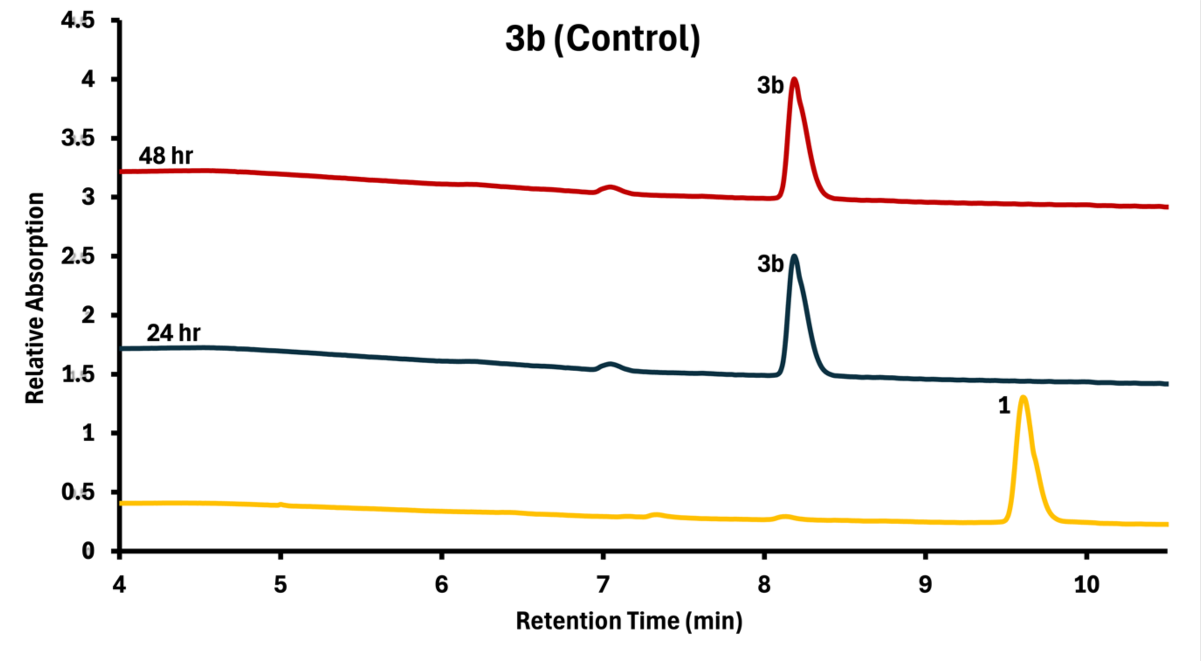

Figure S22: HPLC chromatograms for **3b** in the control group at 24 and 48 hours compared with **1**.

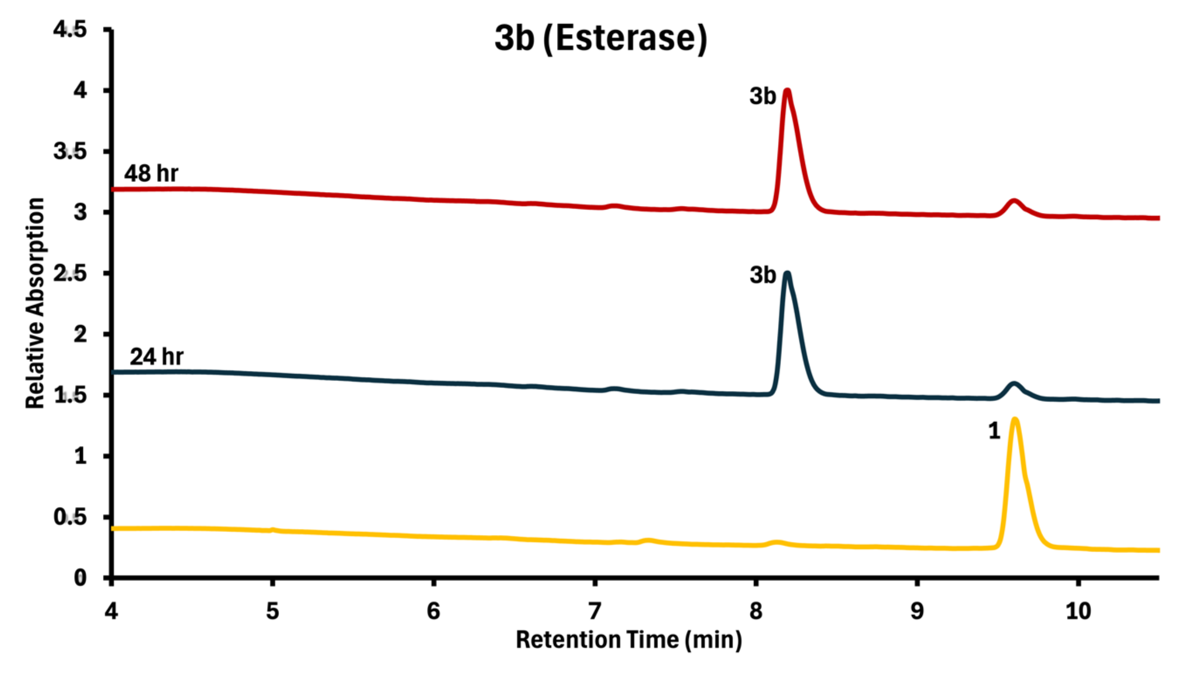

Figure S23: HPLC chromatograms for **3b** in the negative group at 24 and 48 hours compared with **1**.

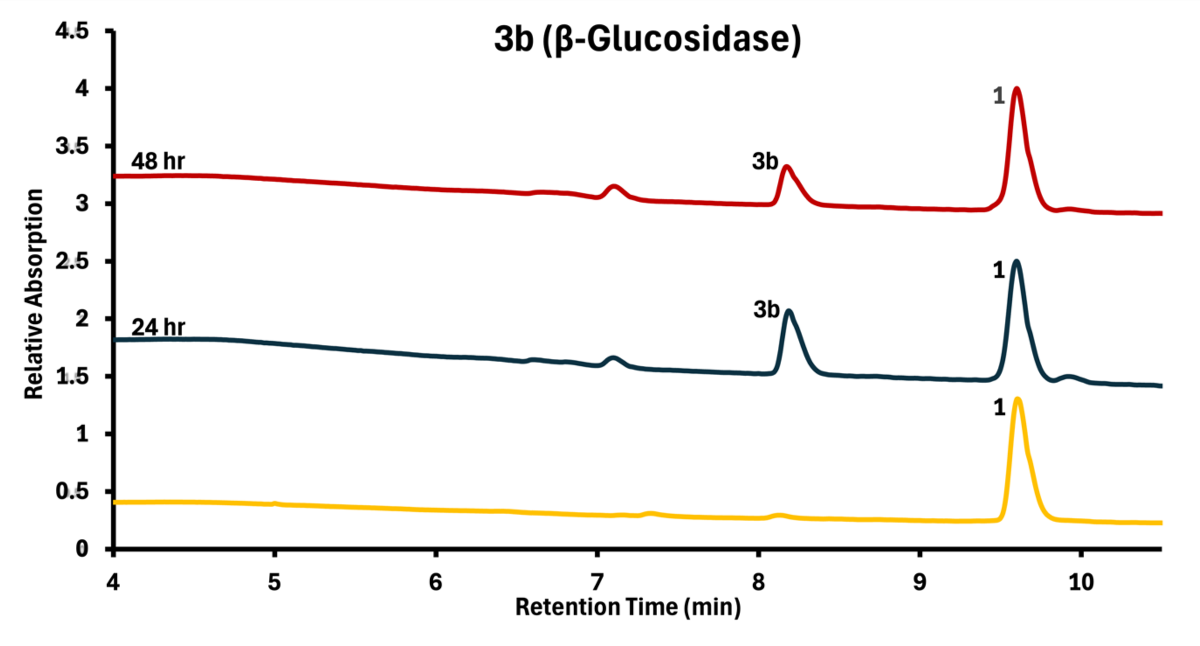

Figure S24: HPLC chromatograms for **3b** in the positive group at 24 and 48 hours compared with **1**.

7. Fluorescence Study

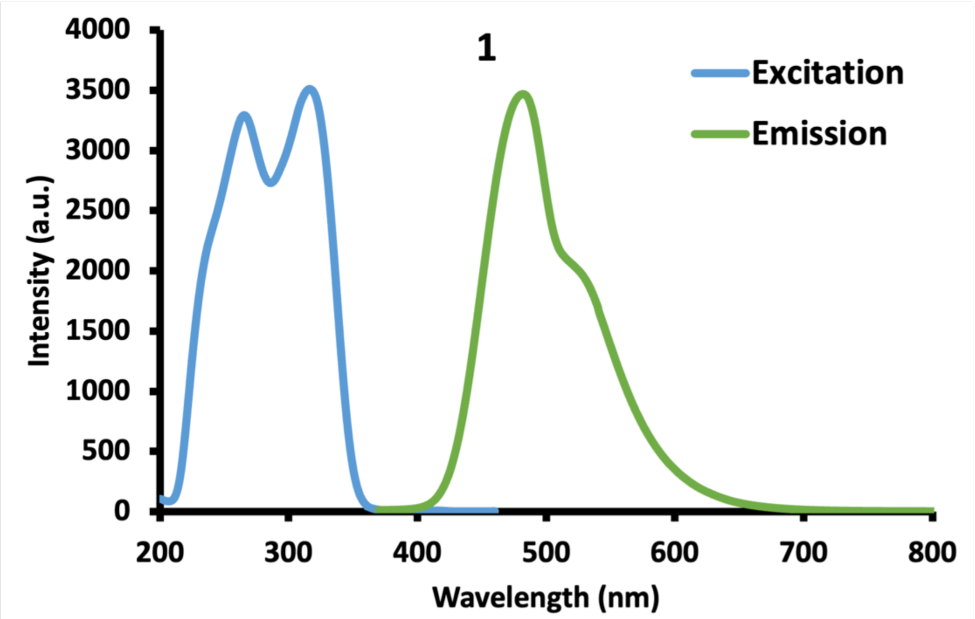

Figure S25: Fluorescence spectra for excitation (blue) and emission (green) of **1** at a concentration of 20 µM in water.

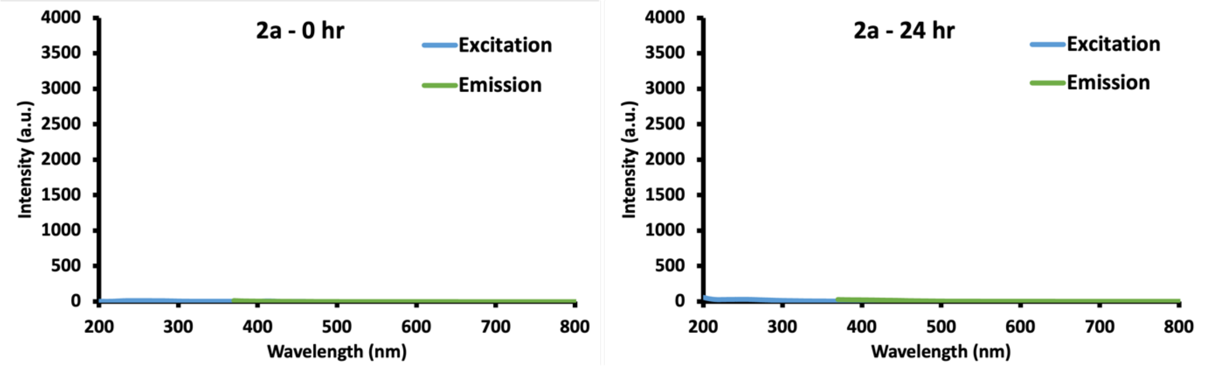

Figure S26: Fluorescence spectra for excitation (blue) and emission (green) of **2a** at a concentration of 20 µM in water before (left) and after (right) incubation with 3 units of β-galactosidase for 24 h.

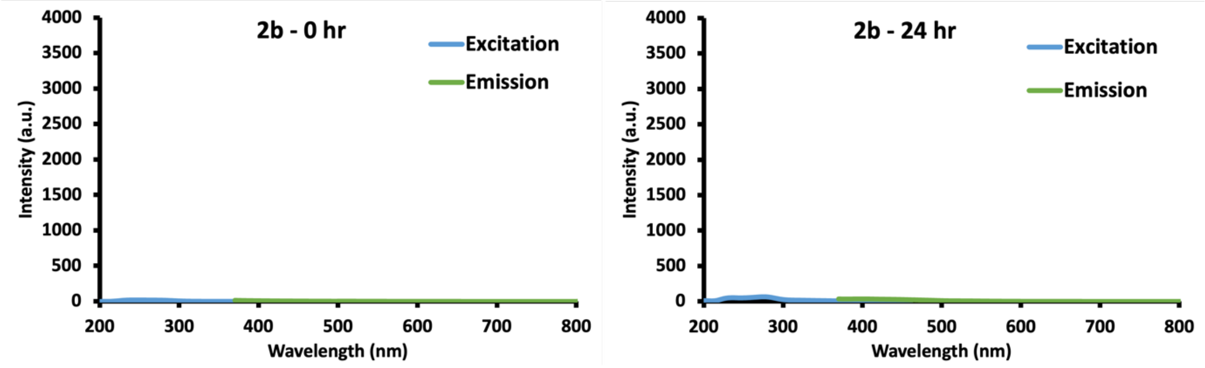

Figure S27: Fluorescence spectra for excitation (blue) and emission (green) of **2b** at a concentration of 20 µM in water before (left) and after (right) incubation with 3 units of β-glucosidase for 24 h.

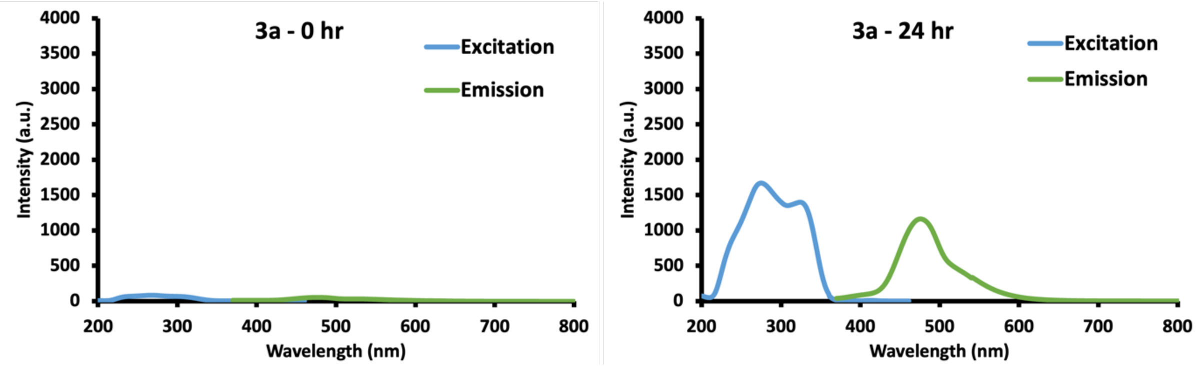

Figure S28: Fluorescence spectra for excitation (blue) and emission (green) of **3a** at a concentration of 20 µM in water before (left) and after (right) incubation with 3 units of β-galactosidase for 24 h.

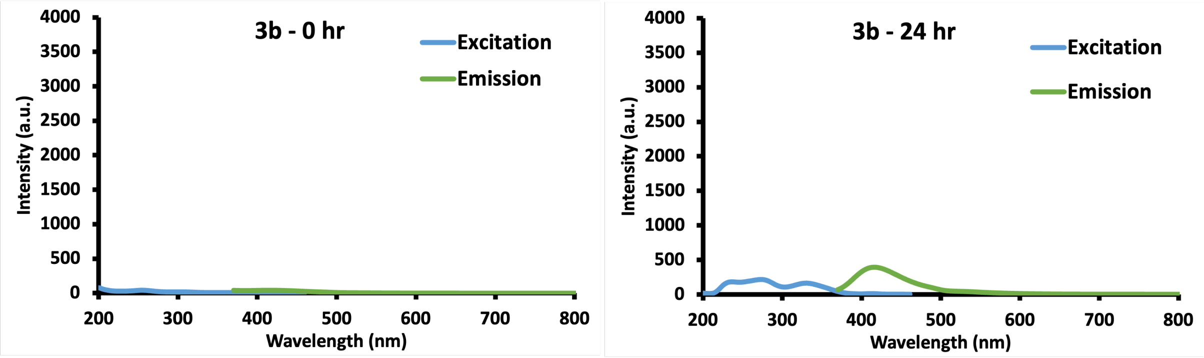

Figure S29: Fluorescence spectra for excitation (blue) and emission (green) of **3b** at a concentration of 20 µM in water before (left) and after (right) incubation with 3 units of β-glucosidase for 24 h.

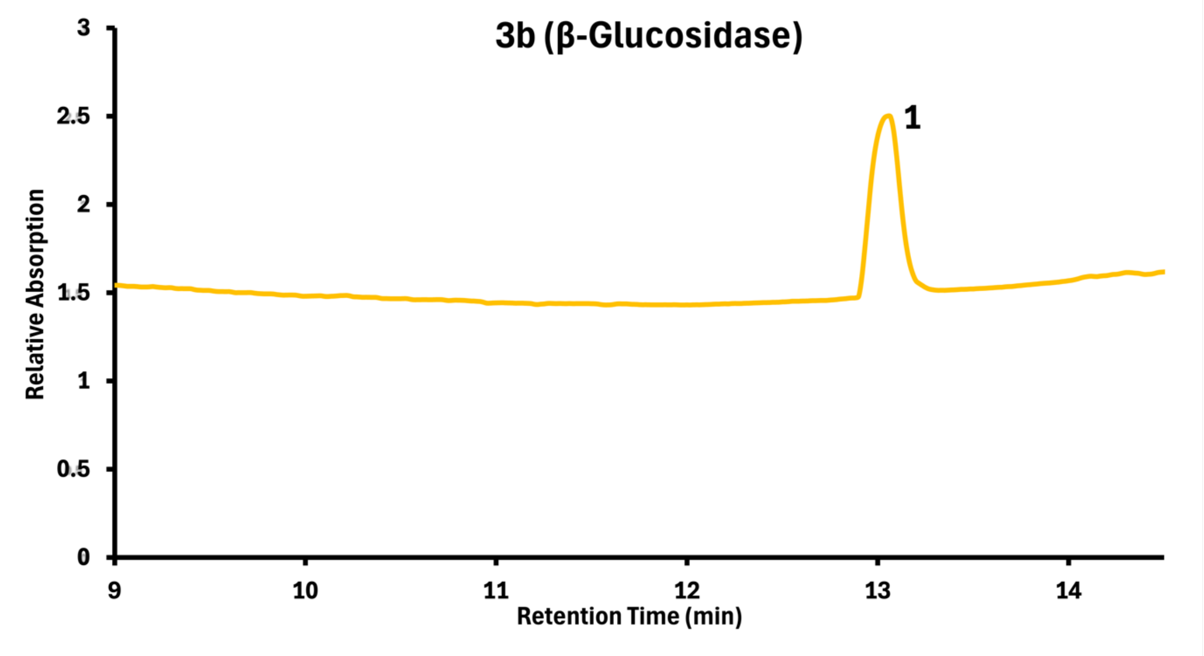

Figure S30: HPLC chromatograms after 24 h incubation of **3b** with 3 enzyme units of β-Glucosidase. This chromatogram was generated from the solution analyzed for fluorescence emission as illustrated in **Figure S28** and serves as confirmation for the successful cleavage of the protecting sugar by the enzyme.

8. Reference

[1] S. Steinhauser, U. Heinz, M. Bartholomä, T. Weyliermüller, H. Nick, K. Hegetschweiler, *Eur J Inorg Chem* **2004**, 4177–4192.
[2] T. Doura, K. Takahashi, Y. Ogra, N. Suzuki, *ACS Med Chem Lett* **2017**, *8*, 211–214.
[3] A. J. Burt, J. D. Hantho, A. E. Nielsen, R. J. Mancini, *Biochemistry* **2018**, *57*, 2184–2188.
